# A tetraazanaphthalene radical-bridged dysprosium single-molecule magnet with a large coercive field

**DOI:** 10.1039/d5sc05358g

**Published:** 2025-10-06

**Authors:** Florian Benner, Saroshan Deshapriya, Selvan Demir

**Affiliations:** a Department of Chemistry, Michigan State University East Lansing Michigan 48824 USA sdemir@chemistry.msu.edu

## Abstract

Generating strong magnetic coupling poses a fundamental challenge in the design of multinuclear lanthanide complexes. The inherently contracted nature of the valence 4f orbitals precludes the lanthanides from engaging in covalent bonding with closed-shell ligands. The employment of open-shell bridging ligands instead allows efficient interaction of the diffuse radical spin orbitals with the 4f shell of the lanthanides. Herein, we introduce the azaacene ligand, 1,4,5,8-tetraazanaphthalene (tan), into rare earth chemistry: first, we synthesized [(Cp*_2_Dy)_2_(μ-tan)] (1, Cp* = pentamethylcyclopentadienyl) containing a diamagnetic tan^2−^ bridge from a salt metathesis reaction of Cp*_2_DyBPh_4_ and K_2_(tan). Second, we chemically oxidised 1 to [(Cp*_2_Dy)_2_(μ-tan˙)][BArF_20_] (2) comprising a tan^1−^˙ radical bridge. 2 is a rare radical-bridged single-molecule magnet (SMM) with open hysteresis loops below 3.75 K with a maximum coercive field (*H*_C_) of 1.373 T at 1.8 K, which represents a notable record as *H*_C_ is approximately doubled compared to all known dinuclear lanthanide SMMs innate to organic radical bridges. A close match of the tan^1−^˙/tan^2−^ and Dy^III^/Dy^II^ redox potentials may be the origin for the impressive hysteresis loops at low temperatures, while the magnetic behaviour at higher temperatures is likely impacted from spin–phonon coupling. The outlined design strategy of matching reduction potentials of the ligand with the metal ions to amplify magnetic coupling, was proposed *via* prior computations, but is within this study for the first time experimentally confirmed. In sum, highly-tunable azaacene radicals have immense potential not only for radical-containing SMMs but for high-performance magnetic materials at large.

## Abstract 2

Strong magnetic exchange coupling in a tetraazanaphthalene radical-bridged dinuclear dysprosium complex enables magnetic memory with a coercive field of 1.373 T, more than twice that of any other organic radical-bridged single-molecule magnet (SMM).

## Graphical abstract

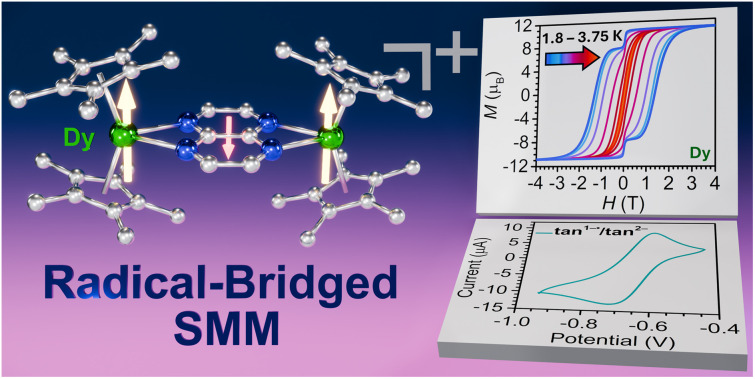

## Introduction

The rapid advancements in artificial intelligence and cloud computing exert significant pressure on existing permanent storage media for digital data.^1,2^ The development of new functional magnetic materials is essential to ensure that magnetic storage capacity continues to evolve in parallel with increasing data storage demands. To replace bulk magnetic materials like SmCo_5_ and Nd_2_Fe_14_B,^3–5^ one of the holy grails of quantum information science has revolved around the storage and manipulating of information in spin orientations on bits of the smallest conceivable unit – a single molecule.^6,7^ However, to date molecular materials that retain magnetic information at high, ideally room temperature have remained elusive.

To this end, lanthanide ions play a crucial role, since their large unquenched orbital momentum and large spin–orbit coupling generate huge single-ion magnetic anisotropies when placed in a suitable crystal field, a circumstance in fact unparalleled in the periodic table. Trivalent dysprosium is highly anisotropic and possesses a doubly degenerate ground state due to its Kramers' ion electronic structure, rendering it particularly relevant for single-molecule magnet (SMM) design.^8,9^ A synthetic challenge to overcome is to couple strongly dysprosium ions to one another which due to the deeply buried 4f shell necessitates to place an unpaired electron with diffuse spin density in between. This can either be achieved by a metal–metal bond approach as shown for [(Cp^iPr5^)_2_Dy_2_I_3_] (Cp^iPr5^ = pentaisopropylcyclopentadienyl) featuring an unpaired electron in a diffuse σ-bonding orbital,^10^ or through the implementation of radical bridging ligands.^11,12^

Importantly, strong magnetic coupling of multiple lanthanide ions through radical ligands suppresses undesirable fast magnetic relaxation pathways such as quantum tunnelling of the magnetisation (QTM).^13,14^

**Fig. 1:**
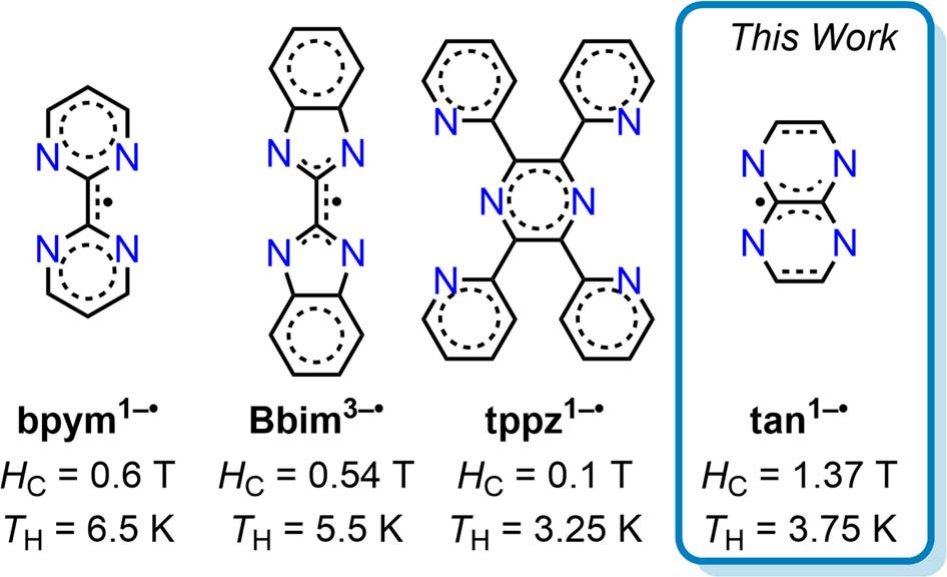
Selected radical ligands used to construct dinuclear dysprosium single-molecule magnets [(Cp*_2_Dy)_2_(μ-L)]X with coercive fields (*H*_C_) and open magnetic hysteresis loops at or below this temperature (*T*_H_) under conventional sweep rates. Ligands L are: bpym = 2,2′-bipyrimidine (X = BPh_4_^−^), Bbim = 2,2′-bisbenzimidazole (X = [K(crypt-222)]^+^, crypt-222 = 2.2.2-cryptand), tppz = 2,3,5,6-tetra(2-pyridyl)pyrazine (X = BPh_4_^−^), tan = 1,4,5,8-tetraazanaphthalene (X = BArF_20_^−^).

Especially, organic radical ligands are highly modifiable through chemical substitution allowing direct tuning of the magnetic exchange coupling and are suitable for the generation of larger molecular clusters. Despite the auspicious prospect, the number of radical-bridged Ln SMMs is small,^15–24^ which is a consequence of the synthetic challenge to tame reactive radicals in between metal centres (Fig. 1).^25–27^

Maximizing magnetic exchange coupling *J* has been considered for long as the primary factor to better performing lanthanide radical-bridged SMMs, however, no clear concepts have emerged to systematically strengthen the magnetic coupling strength or to augment the magnetic hysteresis temperature. This necessitates the scrutiny of new radical bridging ligands ligated to Ln metals in various crystal fields.

Recently, a computational approach was developed to describe the radical–metal interaction, revolving around the Hubbard Model (HM).^28,29^ The HM introduces two tuneable parameters that enter the dominant kinetic exchange interaction: first, the transfer integral, *t*, which refers to the readiness of an electron to move between two magnetic sites. Second, the electronic repulsion, *U*, describing the energy needed to pair electrons on a single site.^30^ These parameters enter the dominant, antiferromagnetic kinetic exchange contribution Δ*J*^A–B^_KE_ as Δ*J*^A–B^_KE_ = −*t*^2^/*U* − *t*′^2^/*U*′, where *t*/*U* denotes Hubbard parameters of one site, and *t*′/*U*′ denotes Hubbard parameters of a second site.^29^

This alludes to a synthesis-guided property design where magnetic coupling may be strengthened by (A) boosting *t e.g.* increasing covalency and orbital overlap with the Dy 4f orbitals, or minimizing *U* (B) by adjusting the radical redox potential to the quite negative redox potential (−2.96 V)^31^ for the Dy^III^/Dy^II^ couple. Redox-active organic molecules innate to negative redox potentials, capable of binding to metals, are rare. To this end, a promising class of molecules with highly negative reduction potentials for use in SMM design are azaacenes which are nitrogen-substituted heteroaromatic carbocycles.

Azaacenes emerged as promising candidates for n-type organic field effect transistors (OFETs), active elements in organic photovoltaic devices (OPVs), and organic light-emitting diodes (OLEDs).^32–34^ The lone electron pairs of the sp^2^ hybridised nitrogen atoms allow azaacenes to coordinate to metal ions,^35,36^ or strongly interact with anionic molecules.^37,38^ While branched, rigid variants such as hexaazatrinaphthalene (han) found entry into coordination chemistry,^17,39,40^ linear azaacene ligands are largely unexplored, especially within the realm of rare earth chemistry. Notably, the redox potentials of linear azaacenes hinge on the number of annulated rings which allows rational tuning.^33,34,41^ Recently, we introduced 5,6,11,12-tetraazanaphthacene (aka fluoflavine) into rare earth chemistry, where first, [(Cp*_2_Y)_2_(μ-flv)] bearing a flv^2−^ ion was isolated which served as a profitable foundation to yield the first radical-bridged complexes containing flv^1−^˙ and flv^3−^˙ radicals through chemical oxidation and reduction, respectively.^42^

The smallest annulated tetraazaacene, 1,4,5,8-tetraazanaphthalene (tan),^43^ represents formally a flv entity contracted by the peripheral phenyl groups, has so far been underutilised in materials design. While tan radicals had been detected,^44–46^ for instance by reacting tan with alkaline earth metals in dimethoxyethane (DME) solution followed by electron paramagnetic resonance (EPR) analysis,^45^ we delivered the first structural evidence for any tan radical.^47^ Our study involved the treatment of tan with potassium graphite in the presence of various chelating agents, leading to isolable compounds innate to differing semiconducting properties.^47^ Herein, we present the first implementation of tan as bridging ligand in any metal complex. To this end, tan^0^ was doubly reduced with KC_8_ to give K_2_tan which was subsequently used in a salt metathesis reaction with Cp*_2_Dy(BPh_4_) to yield [(Cp*_2_Dy)_2_(μ-tan)] (1). 1 allowed access to [(Cp*_2_Dy)_2_(μ-tan˙)][BArF_20_] (2) by one-electron oxidation involving thianthrenium tetrakis(pentafluorophenyl)borate ([Thian˙][BArF_20_]). Excitingly, 2 corresponds to the first tan^1−^˙ radical-bridged complex for any metal ion. Both complexes were fully characterised *via* single-crystal XRD, IR and UV-vis spectroscopy, and cyclic voltammetry. SQUID magnetometry measurements uncovered 2 to be a remarkable SMM with real magnetic memory below 3.75 K. The magnetic hysteresis loop at 1.8 K exhibits a record coercive field *H*_C_ of 1.373 T, surpassing all reported *H*_C_ for dinuclear lanthanide SMMs containing organic radicals by at least a factor of two. The introduction of tan in SMM layout with an amplified magnetic coercivity paves the way for the design of future high-performance magnetic materials.

## Experimental materials and methods

All manipulations were performed under inert conditions using either standard Schlenk techniques employing nitrogen atmosphere or an argon-filled glovebox. House nitrogen was purified through a MBraun HP-500-MO-OX gas purifier. ^*n*^Hexane and dichloromethane (DCM) were purified by refluxing over CaH_2_, toluene and THF were purified by refluxing over potassium and subsequent distillation. THF was subsequently stirred over NaK for at least a day and distilled a second time prior to use. In all cases except for DCM, the solvents were tested for the presence of water and oxygen in the glovebox by the addition of one drop of potassium benzophenone radical solution to 2 mL of the solvent of interest.

The chemicals pentamethylcyclopentadiene (Cp*H), allylmagnesium chloride (2.0 M in THF), 2,3-diaminopyrazine, antimony pentachloride, thianthrene (thian), and anhydrous DyCl_3_ were purchased from Sigma-Aldrich and used as received. Potassium tetrakis(perfluorophenyl)borate (K[BArF_20_]) was purchased from Fischer Scientific and used as received. Potassium bis(trimethylsilyl)amide (KN(Si(CH_3_)_3_)_2_) and 2.2.2-cryptand (crypt-222) were purchased from Sigma Aldrich and were recrystallised from toluene (KN(Si(CH_3_)_3_)_2_) and ^*n*^hexane (crypt-222), respectively. KCp*,^48^ [HNEt_3_][BPh_4_],^49^ Cp*_2_Dy(BPh_4_),^48^ and KC_8_ ^50^ were synthesised according to literature procedures. 1,4,5,8-tetraazanaphthalene (tan^0^) was synthesised *via* condensation of 2,3-diaminopyrazine with glyoxal in H_2_O according to literature procedures,^43,45^ and the yellow precipitate was subsequently extracted *via* a Soxhlet apparatus with acetone overnight. Upon cooling to room temperature, tan^0^ crystallised as yellow needles and, after drying under vacuum for several hours, was used without further purification. [Thian˙][BArF_20_] was quantitatively obtained *via* a salt metathesis reaction of K[BArF_20_] with [Thian˙][SbCl_6_]^51,52^ in DCM in analogy to a procedure reported for [Thian˙][Al{OC(CF_3_)_3_}_4_], and isolated through precipitation in ^*n*^hexane and used without further purification.^53^

Single-crystal XRD data on 1 and 2 were collected on a XtaLAB Synergy DualflexHyPix four-circle diffractometer, equipped with a HyPix Hybrid Pixel Array Detector. The crystals were suspended in ^*n*^paratone oil and mounted on a nylon loop. The temperature during data collection was controlled *via* an Oxford Cryosystems low-temperature device and kept at 100 K during the measurements for all compounds. Data were measured using *ω* scans using CuK_α_ radiation (microfocus sealed X-ray tube, 50 kV, 1 mA). The CrysAlisPro software package^54^ was used to determine the total number of runs and images, to retrieve and refine the cell parameters, and for data reduction. A numerical absorption correction based on Gaussian integration over a multifaceted crystal model empirical absorption correction employing spherical harmonics was accomplished using the SCALE3 ABSPACK^55^ scaling algorithm (spherical harmonics and frame scaling). The structures were solved with the ShelXT^56^ structure solution program using intrinsic phasing and refined with version 2018/3 of ShelXL^57^ using least squares minimisation as implemented in Olex2.^58^ All non-hydrogen atoms are refined anisotropically. Hydrogen atoms were calculated by geometrical methods and refined as a riding model. The crystals used for the diffraction study showed no decomposition during data collection. Crystal data and structure refinement for 1 and 2 are depicted in Table S1.

IR spectra were taken with an Agilent Cary 630 ATR spectrometer residing in a nitrogen-filled glove bag.

UV-vis spectra were collected in an argon-filled glovebox using 1 cm cuvettes with an Agilent Cary 60 spectrometer, equipped with QP600-1-SR fibre optics and a Square One cuvette holder from Ocean Insight. Solvents and concentrations were 32 μmol L^−1^ in DCM (1) and 140/40 μmol L^−1^ in DCM (2).

Cyclic voltammetry experiments were carried out in an argon-filled glovebox deploying a PGSTAT204 potentiostat from Metrohm. A three-electrode setup involving a glassy carbon working electrode, platinum spring counter electrode, and silver wire reference electrode was used. All measurements were performed cycling the solvent range four-fold at a 100 mV s^−1^ scan rate and *E*_1/2_ averaged, where all voltammograms displayed in the main text constitute the second scan. Due to chemical incompatibility of the organometallic complexes with ferrocene, the measurements were externally referenced to ferrocene solutions with identical supporting electrolyte concentrations and electrode setup. Compounds 1 and 2 were measured as 3 mmol L^−1^ DCM solutions. For all measurements, 220 mmol L^−1^ electrolyte concentrations of (^*n*^Bu_4_N)PF_6_ were used.

Elemental analysis was carried out with a PerkinElmer 2400 Series II CHNS/O analyser. The crystalline compounds of all samples (∼1–3 mg) were weighed into tin sample holders and folded multiple times to ensure proper sealing from the surrounding atmosphere. The samples were then transferred to the instrument under exclusion of air in a sealed container.

Magnetic susceptibility data were collected on a Quantum Design MPMS3 Superconducting Quantum Interference Device (SQUID) magnetometer. The magnetic samples of [(Cp*_2_Dy)_2_(μ-tan)] (1) and [(Cp*_2_Dy)_2_(μ-tan˙)][BArF_20_] (2) were prepared by saturating and covering dried, crushed crystalline solids (14.2 mg (1), 21.6 mg (2)) with molten eicosane (26.2 mg (1), 35.1 mg (2)) at 50 °C to prevent crystallite torquing and to provide good thermal contact between the sample and the bath. The samples were sealed airtight and transferred to the magnetometer. The core diamagnetism for both samples were estimated using Pascal's constants.^59^

AC relaxation data were fit using the CCFIT2 program.^60^ Dc relaxation data were fit to a stretched exponential according to eqn (1) using the Origin 9.0.0 b45 software:

1 *M*(*t*) = *M*_eq_ + (*M*_0_ − *M*_eq_) exp(−(*t*/*τ*)^*β*^)

where *M*_0_ is the initial magnetisation, *M*_eq_ is the last fit point (0.01*M*_0_), *β* the stretch factor and *τ* the relaxation time.

Crystal coordinates of 1 and 2 were geometry optimised using TPSSh functional at def2-TZVP theory level.^61^ For Dy^III^ ions, the 4f-in-core potential Stuttgart–Cologne pseudopotential ECP55MWB and the associated ECP55MWB-II basis set were used.^62–65^ Optimised coordinates were used for calculating TD-DFT transitions using the TPSS0 functional and CPCM DCM solvent model. Predicted transitions were shifted by 0.34 eV and 0.42 eV for 1 and 2, respectively, to better match with their experimental UV-vis spectra. For the calculation of estimated exchange coupling, unrestricted density functional theory (DFT) calculations were carried out for a model system of 2 as described below. Crystal coordinates of the heavy atoms for all three molecular units (edge-centred unit and the two disorder parts of the face-centred unit) were used, Dy substituted for diamagnetic Lu, and H atom positions optimised on the DKH-def2-SVP level (SARC2-DKH-QZVP for Lu) using the TPSSh functional with D3BJ dispersion correction.^66–71^ Subsequently, Lu atoms were substituted with Gd for broken-symmetry calculations. These model systems were employed to approximate the exchange coupling strength using the flipspin feature of the ORCA 5.0.4 software.^72–74^ The spin lying on the tan bridge was flipped and the calculations were conducted using the TPSS0 functional at the def2-TZVP theory level.^68,69^ The DKH-def2-TZVP basis set^75^ was used for all atoms and segmented all-electron relativistically contracted (SARC) basis set with quadruple-zeta quality (SARC2-DKH-QZVP) was employed for Gd,^67^ together with the Douglas–Kroll–Hess (DKH) Hamiltonian to account for scalar relativistic effects. The resolution of identity (RI) approximation was used for the Coulomb integrals with the SARC/J auxiliary basis set, while the exchange integrals were treated with the chain-of-spheres approximation (COSX). Grimme's dispersion correction with Becke–Johnson damping (D3BJ) was applied for all calculations.^70,71^ The generation of the spin density and molecular orbital distributions was accomplished employing the orca_plot module and the VMD program was used for orbital visualisations.^76^

### Synthesis of [(Cp*_2_Dy)_2_(μ-tan)] (1)

In a 7 mL scintillation vial, KC_8_ (25.7 mg, 0.1901 mmol) was added to a THF (3 mL) suspension of tan^0^ (12.5 mg, 0.0946 mmol), resulting in an immediate colour change from yellow to black, and the mixture was stirred for 30 min. The reduced tan species, presumably of a dianionic nature, was transferred *via* glass pipette into a 20 mL scintillation vial containing a stirring THF solution (∼5 mL) of Cp*_2_Dy(BPh_4_) (142.5 mg, 0.1894 mmol). Upon addition of K_2_tan, an immediate colour change from pale yellow to intense dark red was observed. The mixture was diluted with THF (resulting in a ∼12 mL solution in total), and, upon stirring at room temperature for 1 h, the colour gradually turned to a lighter red. After stirring for further 15 h, the mixture was filtered through a Celite plug, to remove grey insoluble solids, presumably graphite and KBPh_4_. The solids were washed twice with THF (∼2 mL) and the washings were filtered and combined with the main filtrate. The sum filtrate was evaporated to dryness, affording a red amorphous solid which was extracted with portions of toluene (∼12 mL in total), filtered through a Celite plug, and the clear red filtrate was evaporated to dryness. The resulting red solid was redissolved in ∼6 mL of hot toluene (60 °C), filtered hot through a Celite plug, and once cooled to room temperature, the clear, red filtrate was further cooled in the freezer for crystallisation. Dark red crystals of 1 suitable for single-crystal X-ray diffraction analysis were grown at −35 °C over the course of four days. The crystals were separated from the mother liquor, washed twice with cold toluene (∼2 mL in total), and dried under vacuum for 2 h, yielding a dark red crystalline material of 1 (39.1 mg, 0.0392 mmol, 41%). Dried crystals of 1 are stable at room temperature under an inert Ar atmosphere for several months but quickly degrade under ambient atmosphere. Anal. Calcd for C_46_H_64_Dy_2_N_4_ (1): C, 55.95; H, 6.53; N, 5.67; found: C, 55.83; H, 6.26; N, 5.77. IR (ATR, cm^−1^): 2963 (vw), 2902 (m), 2853 (m), 1769 (vw), 1739 (vw), 1541 (w), 1491 (vw), 1429 (vs), 1409 (vs), 1377 (vs), 1360 (m), 1217 (vw), 1202 (vw), 1169 (vs), 1094 (vw), 1060 (vw), 1019 (w), 880 (vw), 800 (vw), 751 (s), 729 (vw). *λ*_max_ (nm, *ε* in 10^4^ L mol^−1^ cm^−1^): 288 (1.98), 295 (1.97), 487 (0.42), 520 (0.58), 561 (0.48).

### Synthesis of [(Cp*_2_Dy)_2_(μ-tan˙)][BArF_20_] (2)

In a 20 mL scintillation vial, a solution of [Thian˙][BArF_20_] (40.0 mg, 0.0445 mmol, 1 mL DCM) was added to a stirred solution of 1 (40.2 mg, 0.0403 mmol) in DCM (3 mL), resulting in an immediate colour change from red to black. The mixture was slightly diluted with DCM (final volume: 5 mL), and then stirred at room temperature for 20 min. The reaction mixture was evaporated to dryness under stirring to yield a black solid which was extracted three times with toluene (by using ∼8 mL in total) and removed an unidentified orange byproduct. The toluene insoluble solids were dried under vacuum for 2 h, dissolved in DCM (∼2 mL) and then layered with ^*n*^hexane (∼1 : 1 DCM/^*n*^hexane ratio) and cooled to −35 °C for crystallisation. Black crystals of 2 suitable for single-crystal X-ray diffraction analysis were grown at −35 °C over the course of 3 days. After removing the weakly coloured mother liquor, the crystals were washed twice with a 1 : 1 DCM/^*n*^hexane mixture (∼0.5 mL each) and then dried under vacuum for 2 h, yielding black crystalline material of 2 (53.3 mg, 0.0318 mmol, 79%). Dried crystals of 2 are stable at −35 °C under an inert Ar atmosphere for several months. Anal. Calcd for C_70_H_64_BDy_2_F_20_N_4_ (2): C, 50.13; H, 3.85; N, 3.34; found: C, 49.93; H, 3.81; N, 3.27. IR (ATR, cm^−1^): 2913 (vw), 2905 (vw), 2864 (vw), 1642 (vw), 1511 (m), 1487 (w), 1461 (vs), 1392 (w), 1379 (m), 1275 (w), 1269 (w), 1195 (m), 1085 (s), 1019 (vw), 977 (vs), 913 (vw), 898 (vw), 822 (m), 773 (m), 755 (s), 740 (vw), 725 (vw), 682 (m), 660 (s). *λ*_max_ (nm, *ε* in 10^4^ L mol^−1^ cm^−1^): 355 (1.34), 649 (0.14), 682 (0.15), 716 (0.17), 797 (0.16).

## Results and discussion

### Synthesis, structural and spectroscopic characterisation

The synthesis of tan radical-bridged lanthanide complexes was inspired by our deployed strategy to gain access to a flv^1−^˙ radical-bridged metal complex, by generating first a neutral molecule composed of a diamagnetic flv^2−^ bridge followed by chemical oxidation.^42^ In contrast to flv, tan lacks NH protons and thus the diamagnetic tan^2−^ was pursued *via* chemical reduction.

**Fig. 2:**
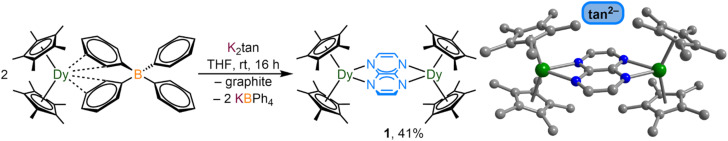
Synthesis of [(Cp*_2_Dy)_2_(μ-tan)] (1) *via* salt metathesis of Cp*_2_Dy(BPh_4_) with K_2_tan (left). Structure of the tan^2−^ bridged complex 1, obtained through single-crystal X-ray diffraction analysis (right). Green, blue, and grey spheres represent dysprosium, nitrogen, and carbon atoms, respectively. All hydrogen atoms and solvent molecules are omitted for clarity.

First, a THF suspension of tan^0^ was treated with two equivalents of KC_8_ to yield a black suspension which was used *in situ* by a quantitative transfer onto a stirring pale yellow THF solution comprising two equivalents of Cp*_2_Dy(BPh_4_) (Fig. 2). An immediate colour change to dark red was observed, which gradually turned to a lighter red while stirring over the course of 1 h. After 16 h of reaction time, first workup of the mixture and then crystallisation followed from a hot concentrated toluene solution, allowing isolation of [(Cp*_2_Dy)_2_(μ-tan)] (1) as dark red crystals in 41% yield.

1 constitutes the first crystallographic evidence of a tan^2−^-containing complex and simultaneously the first introduction of tan into rare earth metal chemistry. 1 can be crystallised from various solvents, including concentrated DCM or THF solutions, however, hot toluene crystallisations gave reproducibly the highest yields.

Complex 1 comprises two dysprosium(iii) ions, each η^5^-ligated by two Cp* ligands, and a tetradentate tan^2−^-ligand that is coordinated to both metal centres slightly asymmetrically through two nitrogen atoms on each site, ultimately acting as a bridge (Fig. 2). The asymmetric unit of 1 consists of one dysprosocenium moiety ligated by half a tan^2−^ unit due to a molecule-inherent inversion centre. The intramolecular Dy⋯Dy distance of 6.978(1) Å is considerably shorter than the closest intermolecular Dy⋯Dy distance of 8.477(1) Å (Table 1). A comparison of the intra-tan^2−^ distances to our 2,2′-bisbenzimidazole (Bbim) bridged dysprosium complex^42^ offers an appealing opportunity to assess the structural impact of different bridging ligands.

**Table 1:** Selected interatomic distances (Å) and angles (deg) of the tan-bridged Dy complexes, [(Cp*_2_Dy)_2_(μ-tan)] (1) and [(Cp*_2_Dy)_2_(μ-tan˙)][BArF_20_] (2)

	1	2 (M1)^a^	2 (M2)^b^
Tan oxidation state	2−	1−·	1−·
Dy–N	2.377(2); 2.365(2)	2.445(3); 2.436(3)	2.448(4); 2.453(4)
Dy–C (central)	2.771(3)	2.852(3)	2.878(4)
C–C (central)	1.443(6)	1.436(6)	1.411(7)
Dy⋯Dy	6.978(1)	7.139(1)	7.165(1)
Dy–C (avg.)	2.650(8); 2.656(11)	2.629(3)	2.644(7); 2.611(8)
Cnt^c^–Dy	2.406; 2.325	2.333; 2.335	2.335; 2.327
	2.388; 2.334		2.374 : 2.297
Cnt–Cnt_PhF5_^d^	—	4.047	—
Cnt–Cnt^e^	—	—	4.394; 4.077
Cnt–Dy–Cnt	143.9; 139.5	143.9	144.2; 144.2
Dy–N–N′–Dy′	26.4(4)	5.2(5)	7.3(7)
Pz_Plane1_–Pz_Plane2_^f^	0.1(1)	0.1(1)	0.1(1)
tan_plane_–tan_plane_^g^	84.8(1)	7.4(2)	
tan_line_–tan_line_^h^	84.8	48.1	

a Edge-centred molecule.

b Face-centred molecule.

c Cnt = centroid of the pentamethylcyclopentadienyl ring.

d Distance between adjacent Cp* and BArF centroids.

e Distance between adjacent Cp* centroids.

f Intramolecular angle between pyrazine (pyz) rings.

g Intermolecular angle between tan planes.

h Intermolecular angle between lines bisecting the tan ligands.

Comparing 1 to [(Cp*_2_Dy)_2_(μ-Bbim)], the central C–C bond of the tan ligand is almost identical with 1.443(6) Å (deviation *Δ* = 0.012 Å).^18^ The average metal–nitrogen distance is considerably contracted by 0.052 Å from 2.423(4) Å in the Bbim complex to 2.371(3) Å in 1. Most prominently, the Dy⋯Dy distance is substantially contracted by 0.775 Å in 1, which stems from reduced steric demand of the tan *versus* the Bbim ligand. This also gives rise to a more pronounced out-of-plane displacement of the Dy centres *versus* the bridging ligand as suggested by the larger Dy–N–N′–Dy′ angle of 26.4(4)° in 1 relative to 11.8(3)° in [(Cp*_2_Dy)_2_(μ-Bbim)].

Cyclic voltammetry experiments hinted at the electrochemical accessibility of the tan^1−^˙ oxidation state (see below), which prompted the synthesis of the tan^1−^˙ radical-bridged Dy complex [(Cp*_2_Dy)_2_(μ-tan˙)]^+^ (2) *via* chemical oxidation of 1. A DCM solution of 1 was exposed to the oxidant thianthrenium tetrakis(pentafluorophenyl)borate [Thian˙][BArF_20_] affording a rapid colour change from intense red to black, indicative of the formation of a tan^1−^˙ radical-containing compound (Fig. 4). 2 was crystallised from ^*n*^hexane layering of a concentrated DCM solution at −35 °C as black blocks in 79% yield over three days. Excitingly, 2 constitutes the first example of a crystallographically characterised coordination complex bearing a tan radical.

SCXRD analysis of 2 confirmed the topological retention of the tan-bridged complex after oxidation and the presence of the [BArF_20_]^−^ counter anion in the crystal lattice which proves electron transfer onto the cationic metal complex indicating an oxidation state change of the bridging tan ligand (Fig. 4). This is reflected in the elongation of the Dy–N distances by ∼0.08 Å and in the Dy⋯Dy distances by 0.16–0.19 Å, in accordance with a smaller charge of the tan bridge in 2. Similar to 1, the Dy–N distances in 2 are slightly asymmetric (Table 1). Relative to the bpym^1−^˙-containing Dy complex [(Cp*_2_Dy)_2_(μ-bpym˙)][BPh_4_] (bpym = 2,2′-bipyrimidine), these distances are slightly elongated with an average Dy–N distance of 2.42(1) Å compared to 2.446(4) Å in 2.^24^ The average Dy⋯Dy distance is significantly longer by *Δ* = 0.727 Å relative to the bpym^1−^˙-containing Dy complex, which correlates with a decreased Dy–N–N′–Dy′ angle by 10.6° due to reduced steric demand of the smaller tan ligand compared to the bpym ligand. Furthermore, these trends highlight the primarily ionic bonding prevalent in this series of complexes.

2 crystallises in the triclinic space group *P*1̄, featuring two inversion-symmetric half molecules in the asymmetric unit. One of these units is positioned on the cell edges, while the second resides face-centred on the crystallographic b-phase (Fig. S4). The closest intermolecular Dy⋯Dy distances of 8.594(1) Å are found between the face-centred molecules and are considerably longer than the intramolecular Dy⋯Dy distance. Hence, the magnetic properties are expected to be dominated by strong Dy-radical magnetic coupling.

### Spectroscopy

Infrared spectra were collected for 1 and 2 (Fig. S7), which exhibit signs of successful oxidation from 1 to 2. Particularly the emergence of two well-isolated strong bands at 1085 cm^−1^ and 977 cm^−1^ are diagnostic, and their frequencies align well with symmetric and antisymmetric C–F stretches of other compounds (Fig. S7).^77^ Similar bands were found for Li[BArF_20_] at 1087 cm^−1^ and 980 cm^−1^,^78^ and for organometallic complexes containing the BArF_20_ counterion such as [Cp^iPr5^DyCp*][BArF_20_] and [Cp^ttt^_2_Dy][BArF_20_], which show bands at 1084/978 cm^−1^ and 1084/977 cm^−1^, respectively, indicative of the counterion.^79,80^ A third strong band at 1461 cm^−1^ overlaps with several weaker bands and is also attributed to a symmetric C–F stretching vibration, which interpretation is supported by the peaks relative intensity matching the approximately 1 : 0.8 : 1 intensity pattern observed in mononuclear dysprosium complexes with [BArF_20_]^−^ counter ions.^77,78,81^

UV-vis spectra were taken for 1 and 2 and deliver also signatures for a change in oxidation state of the tan bridge (Fig. 5, S8 and S9). As suggested by the red colour of the crystalline material, 1 forms an intense red solution when dissolved in DCM and exhibits strong absorptions in the visible region at 487, 520, 561 nm, and in the UV-region at 288 and 295 nm. The oxidation of 1 with [Thian˙][BArF_20_] is accompanied by an immediate colour change from red to black. The UV-vis spectrum of 2 is vastly different from that of 1 and features broad absorption across the entire visible region with weak maxima at 649, 682, 716 and 797 nm in addition to one UV transition at 350 nm. The fact that all visible transitions assigned in 1 vanished after oxidation of the compound to 2 further alludes to the ligand-based nature of these transitions.

To further scrutinise the differences in the UV-vis spectra of the two compounds, TD-DFT calculations were performed on the geometry optimised structures of 1 and 2, employing a DCM solvent model. The most intense transition for 1 is predicted to be at 499.6 nm (2.00 × 10^4^ cm^−1^) and arises due to a transition from a tan-based highest occupied molecular orbital (HOMO) to primarily Cp* and tan-based lowest unoccupied molecular orbital (LUMO)+12. The second strongest absorption originates primarily from an excitation at 550.8 nm (1.82 × 10^4^ cm^−1^) owing to a Cp* ligand-based HOMO−3 to tan-based LUMO.

In contrast to 1, the TD-DFT calculation of 2 predicts multiple transitions throughout the visible region, consistent with the black colour. Several prominent transitions occur at around ∼500 nm and stem from excitations primarily from Cp*-based occupied orbitals to the tan-based singly occupied molecular orbital (SOMO) and the LUMO. A transition at 487.1 nm (2.05 × 10^4^ cm^−1^) is due to a HOMO to LUMO+2 transition where the virtual MO is metal-based. The next set of most intense transitions are positioned at approximately ∼400 nm and are all ligand-to-ligand charge transfers from tan-based occupied MOs to tan-based virtual MOs.

In addition, several TD-DFT transitions in 2 are predicted towards lower wavelengths. Several ligand-to-ligand charge transfers arising from the Cp*-based MOs to the LUMO generate the prominent transition at 565.1 nm (1.77 × 10^4^ cm^−1^). Another intense transition at 693.1 nm (1.44 × 10^4^ cm^−1^) is due to a Cp*-based HOMO−6 to SOMO transition. Thus, regardless of the structural similarities, the electronic absorption spectra of 1 and 2 significantly differ from each other owing to the excitations mainly being tan-centred. Tables S5 and S6 in the SI provide more details regarding the individual TD-DFT transitions.

### Cyclic voltammetry measurements

**Fig. 3:**
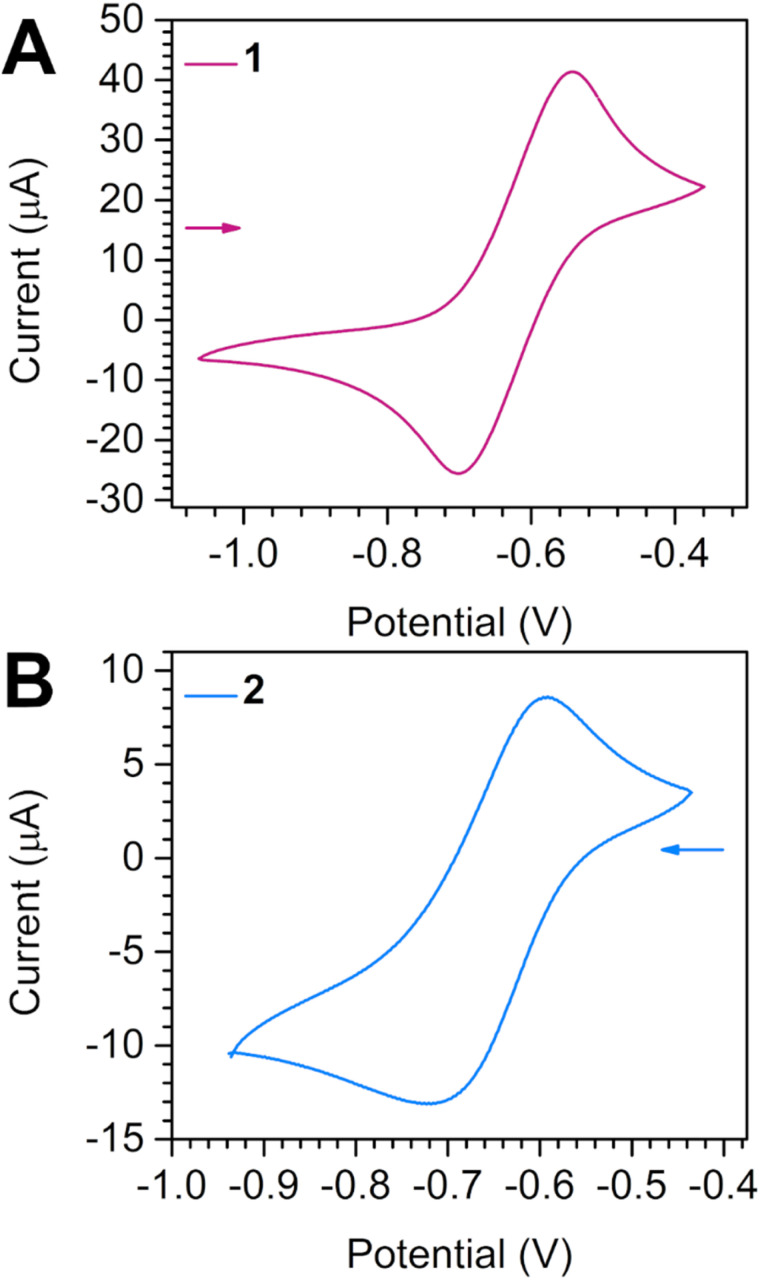
Cyclic voltammograms of [(Cp*_2_Dy)_2_(μ-tan)] (1) (A) and [(Cp*_2_Dy)_2_(μ-tan˙)][BArF_20_] (2) (B), measured in DCM at 300 K with 0.22 mmol L^−1^ [^*n*^Bu_4_N][PF_6_] supporting electrolyte and 3 mmol L^−1^ analyte concentration against a Ag wire pseudo reference electrode with a 100 mV s^−1^ scan rate.

Cyclic voltammograms of 1 and 2 were taken in DCM solutions in the presence of [^*n*^Bu_4_N][PF_6_] supporting electrolyte (Fig. 3, S10, S11 and Table 2). For 1, through scanning from strongly negative potentials towards positive potentials, two quasi-reversible features at −0.62(2) V and +0.34(4) V were observed, and an irreversible oxidation event was monitored at ∼+0.5 V (*versus* Fc^+^/Fc). The quasi-reversible peaks were assigned to a tan^1−^˙/tan^2−^ and a tan^0^/tan^1−^˙ redox event, respectively. By contrast, the irreversible oxidation may be associated with the decomposition of 1 through tan^0^ precipitation and/or deposition on the electrode surface. Especially the moderately negative potential of the tan^1−^˙/tan^2−^ redox couple suggests facile electron removal from the diamagnetic tan^2−^ bridge.

**Fig. 4:**
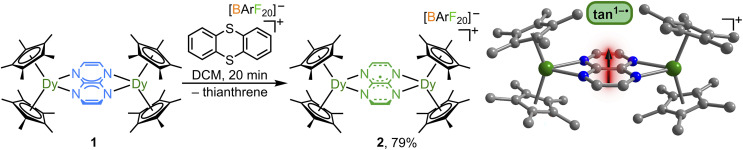
Synthesis of [(Cp*_2_Dy)_2_(μ-tan˙)][BArF_20_] (2) through oxidation of [(Cp*_2_Dy)_2_(μ-tan)] (1) with [Thian˙][BArF_20_] (left). Structure of 2, obtained through single-crystal X-ray diffraction analysis (right). Green, blue, and grey spheres represent dysprosium, nitrogen, and carbon atoms, respectively. All hydrogen atoms and the counter ion [BArF_20_]^−^ are omitted for clarity.

**Fig. 5:**
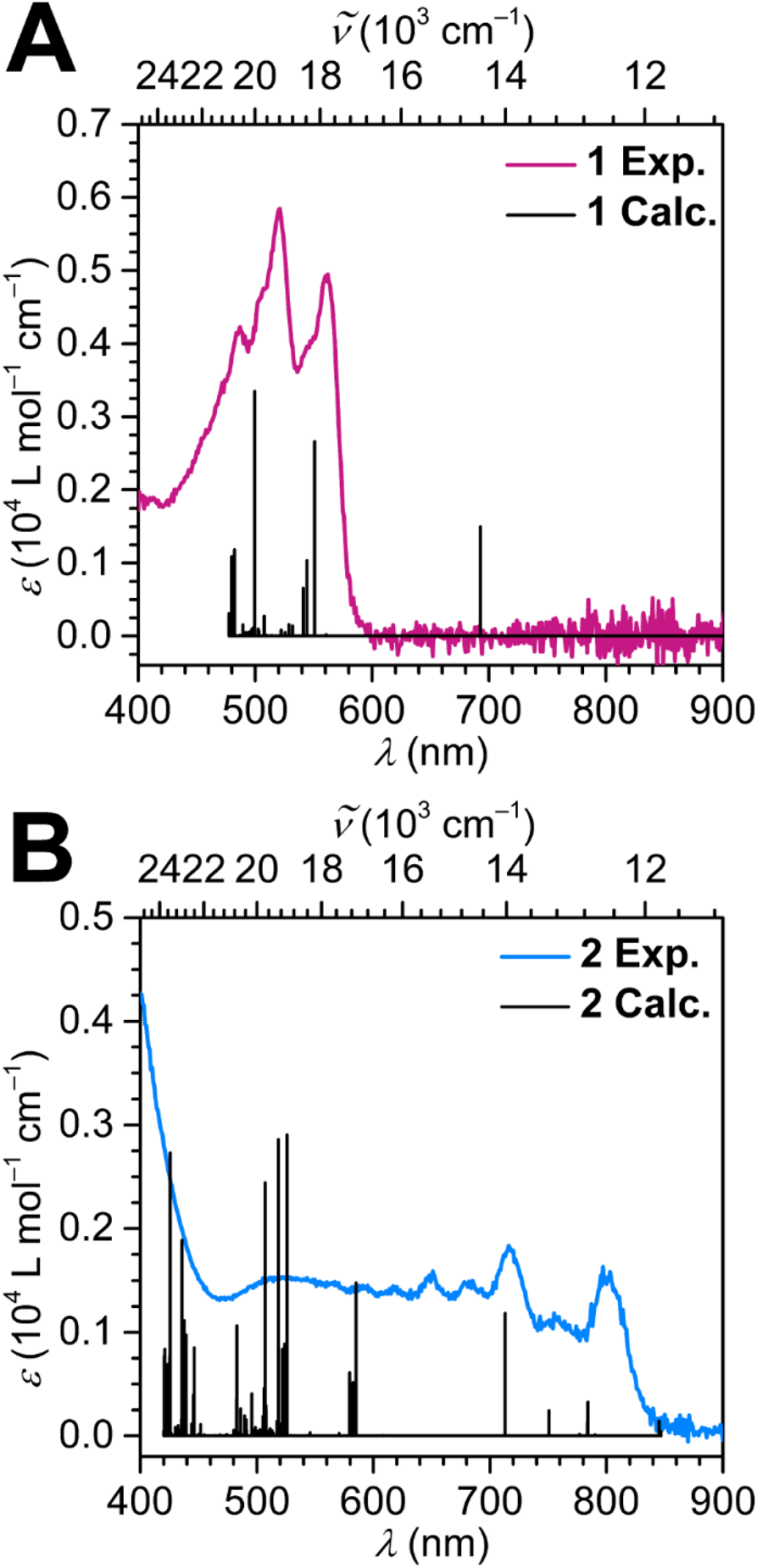
UV-vis spectra of (A) [(Cp*_2_Dy)_2_(μ-tan)] (1) (pink line), and of (B) [(Cp*_2_Dy)_2_(μ-tan˙)][BArF_20_] (2) (blue line), recorded at 32 μmol L^−1^ (1) and 140 μmol L^−1^ (2) concentrations in DCM at room temperature. Black vertical lines represent calculated TD-DFT transitions.

**Table 2:** Redox potentials of compounds containing flv^1−^˙ and tan^1−^˙ ligands *versus* the Fc^+^/Fc^0^ couple

Compound	*E*_1/2_ (1) (V) L^0^/L^1−^˙	*E*_1/2_ (2) (V) L^1−^˙/L^2−^	Reference
[K(crypt-222)][flv˙]	−0.902(4)	−1.608(3)	42
[K(crypt-222)][tan˙]	−1.04	−1.96	47
[(Cp*_2_Y)_2_(μ-flv˙)][Al(OC{CF_3_}_3_)_4_]	—	−0.935(2)	42
[(Cp*_2_Dy)_2_(μ-tan˙)][BArF_20_] (2)	—	−0.65(2)	This work
[(Cp*_2_Dy)_2_(μ-tppz˙)][BPh_4_]	−0.64	−1.36	16

Subjecting a 3 mmol L^−1^ solution of 2 in DCM to −2 and +0.5 V applied potentials (*versus* Fc^+^/Fc) revealed one quasi-reversible feature at −0.65(2) V which is in excellent agreement with the tan^1−^˙/tan^2−^ potential determined for 1. In contrast, 2 exhibits two additional irreversible oxidation features at ∼−0.15 V and ∼+0.06 V. In the same region, 1 exhibits one quasi-reversible feature for the tan^0^/tan^1−^˙ process at −0.62(2) V. Redox couples alike to one another were also observed in a series of uranium complexes [(({Me_3_Si}_2_N)_3_U)_2_(μ-bpym)]X (bpym = 2,2′-bipyrimidine, X = BPh_4_, 0, [K(crypt-222)] and [K(crypt-222)]_2_), containing the bpym bridging ligand in various oxidation states (1−·, 2−, 3−· and 4−).^82^ In this example, *E*_1/2_ for the bpym^1−^˙/bpym^2−^ redox event were monitored at −0.95 V and −0.93 V, respectively, for the corresponding complexes bearing bpym^1−^˙ and bpym^2−^ bridges. Similar electrochemical trends also occurred in a series of dysprosium tetraoxolene complexes [((HBpz_3_)_2_Dy)_2_(μ-ba)] and [Cp_2_Co][((HBpz_3_)_2_Dy)_2_(μ-ba˙)] (HBpz_3_^−^ = hydrotris(pyrazolyl)borate; ba = bromanilate), bearing ba in the 2− and 3−· oxidation states.^83^

Compared to our reference complex [(Cp*_2_Y)_2_(μ-flv˙)][Al(OC{CF_3_}_3_)_4_], the *E*_1/2_ for the flv^1−^˙/flv^2−^ process appears at −0.935(2) V (*versus* Fc^+^/Fc in difluorobenzene), which is significantly shifted by 0.285 V towards negative potentials compared to 2.^42^ At first glance, this is counterintuitive as in linear azaacenes, the HOMO and LUMO energies strongly hinge on the number of rings and substituents in the molecule.

Namely the LUMO energy declines with rising number of rings, while the HOMO energy increases resulting in a net decrease in the HOMO–LUMO gap through the ring addition.^84,85^ Likewise, chemical substitution with electron-accepting groups is expected to shrink the HOMO–LUMO gap.^86^ However, these trends have been established considering closed-shell azaacenes, and reduced open-shell molecules are largely unexplored, let alone when bound to metal ions. In a rare case study, we discovered that both the HOMO–LUMO gap and flv^1−^˙/flv^2−^ redox potential of the free flv^1−^˙ radical shrink upon coordination to Lewis-acidic yttrium ions.^42^ Hence, the net decrease in *E*_1/2_ observed for 2*vs.* the flv^1−^˙ reference complex probably originates from a combination of compensatory influences due to the formal contraction of the flv ligand by two Ph rings and double dysprosium coordination.

Recently, some of us reported the electrochemical properties of the free tan^1−^˙ radical in the form of [K(crypt-222)][tan˙] and [K(18-c-6)][tan˙].^47^ For [K(crypt-222)][tan˙], one quasi-reversible feature corresponding to the tan^1−^˙/tan^2−^ process was found at −1.96 V, and a second quasi-reversible feature for the tan^0^/tan^1−^˙ process was observed at −1.04 V (*versus* Fc^+^/Fc). Compared to [K(crypt-222)]flv˙, the *E*_1/2_ for the flv^1−^˙/flv^2−^ (−1.608(3) V) and the flv^0^/flv^1−^˙ processes (−0.902(4) V) are considerably shifted towards negative potentials due to formal removal of the peripheral Ph rings from flv.^42^

### Static magnetic susceptibility measurements

**Fig. 6:**
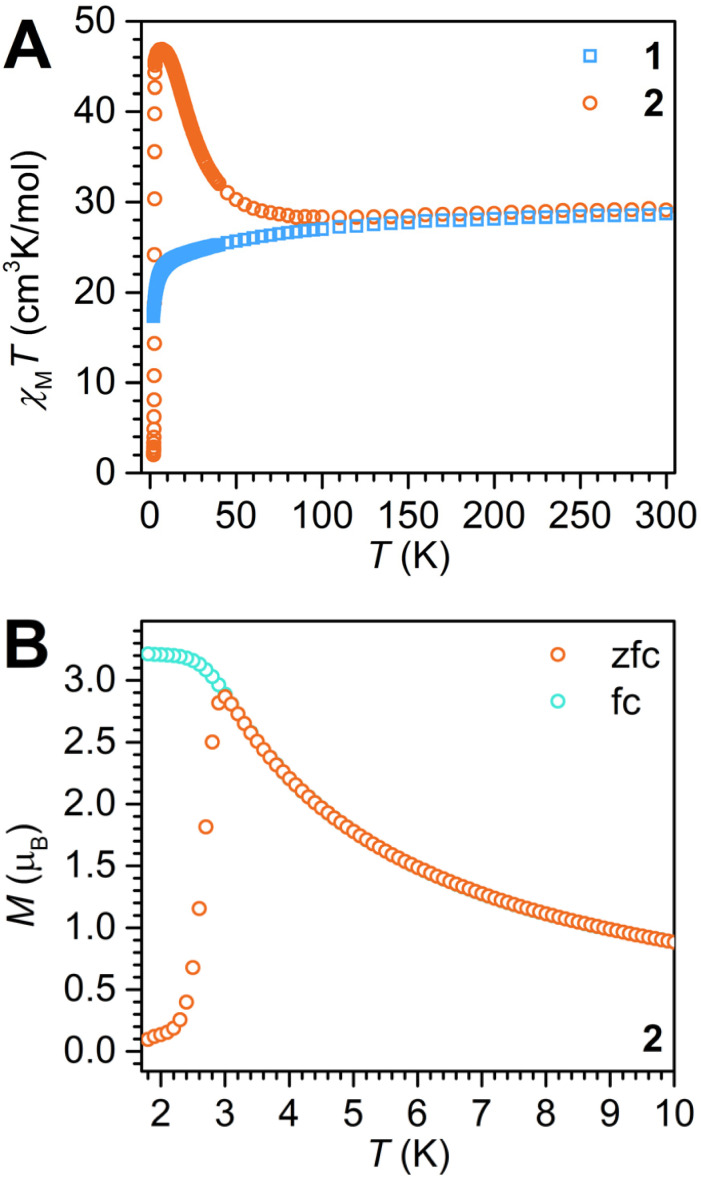
(A) Variable-temperature dc magnetic susceptibility data for restrained polycrystalline samples of 1 (blue squares) and 2 (orange circles), respectively, collected under a 0.1 T applied dc field. (B) Plot of magnetisation *vs.* temperature for 2 during field-cooled (turquoise circles) and zero-field-cooled (orange circles) measurements displaying the thermoremanent magnetisation. 1.0 T data are displayed in Fig. S12 and S13.

The magnetic exchange coupling between the Dy^III^ ions and the tan^1−^˙ radical-bridge was first canvassed through the measurement of the temperature dependence of the product of magnetic susceptibility and temperature (*χ*_M_*T vs. T*). Measurements were carried out on a polycrystalline sample of 2 at 0.1 T and 1.0 T applied direct current (dc) fields between 2 and 300 K (Fig. 6 and S12). In the following, the data collected under a 0.1 T applied dc field will be discussed. The acquired data under a 1.0 T field is available in the SI.

At 300 K, the *χ*_M_*T* value of 29.12 cm^3^ K mol^−1^ is in good agreement with the expected value of 28.71 cm^3^ K mol^−1^ for two magnetically isolated Dy^III^ ions (*J* = 15/2 and *g*_J_ = 4/3) and a radical spin (*S* = ½ and *g* = 2.00). As the temperature is lowered, the *χ*_M_*T* product gradually declines to a minimum value of 28.31 cm^3^ K mol^−1^ at 95 K. With further decreasing temperature, a pronounced rise in *χ*_M_*T* occurs culminating in a maximum value of 46.88 cm^3^ K mol^−1^ at 7 K, before displaying a steep drop to 2.03 cm^3^ K mol^−1^ at 2 K.

The progression of *χ*_M_*T vs. T*, in particular the occurrence of a shallow minimum at 95 K in *χ*_M_*T*, and the pronounced maximum reached at 7 K, suggests the presence of strong antiferromagnetic coupling between the tan^1−^˙ radical and the Dy^III^ centres, giving rise to the formation of a high-angular momentum. The steep drop at the lowest temperatures is attributed to magnetic blocking which refers to a situation where the orientation of the magnetic moment is pinned by the strong magnetic anisotropy, rendering it incapable to follow the external field. This is further corroborated by the sharp divergence of zero-field-cooled (zfc) and field-cooled (fc) *χ*_M_*T* data at 3 K. A magnetic blocking event is exciting as it implies that at those temperatures the molecule could retain information.

Dc magnetic susceptibility measurements were also taken on a polycrystalline sample of 1 under 0.1 T and 1.0 T (Fig. 6 and S12). Under 0.1 T and 300 K, the *χ*_M_*T* value is 28.67 cm^3^ K mol^−1^ which is in good accordance with the anticipated value of 28.33 cm^3^ K mol^−1^ for two magnetically uncoupled Dy^III^ ions. Upon lowering the temperature, *χ*_M_*T* gradually decreases until ∼17 K, after which a more significant downturn occurs which is ascribed to thermal depopulation of low-lying excited states and/or antiferromagnetic coupling. Relative to 2, magnetic blocking features are absent, as expected.

### Dynamic magnetic susceptibility measurements

**Fig. 7:**
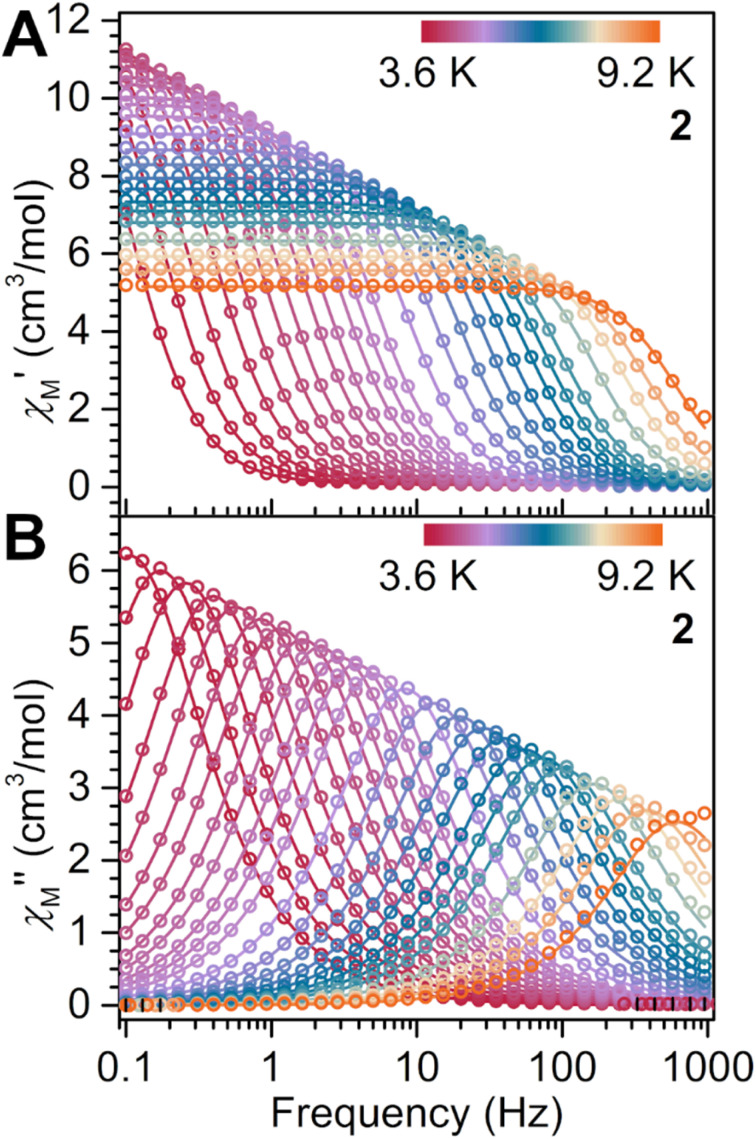
(A) Variable-temperature, variable-frequency in-phase (*χ*_M_′) and (B) out-of-phase (*χ*_M_′′) ac magnetic susceptibility data collected under a zero Oe applied dc field for 2, from 3.6 to 9.2 K. Solid lines indicate fits to the generalised Debye model.

**Fig. 8:**
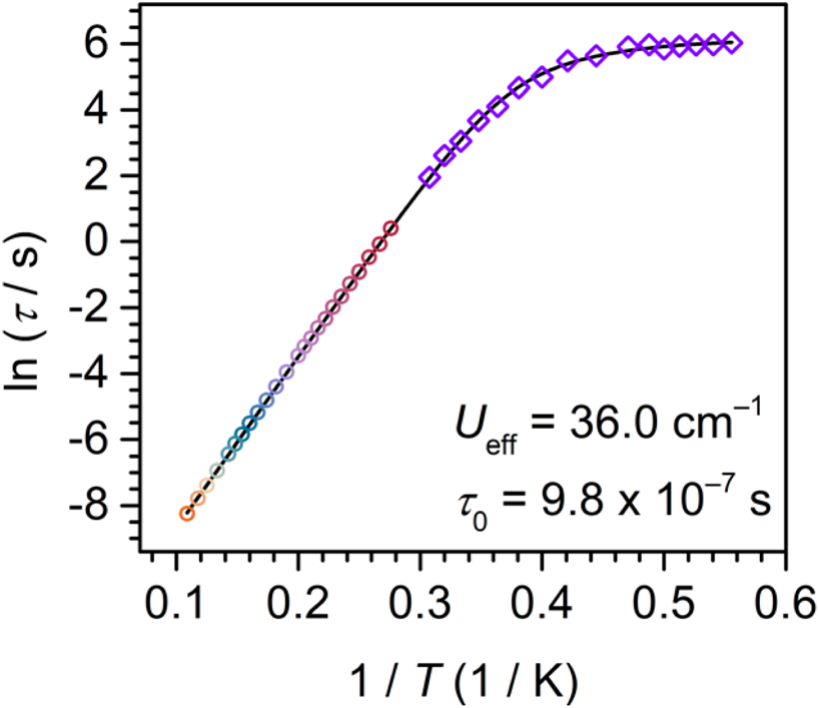
Plot of natural log of the relaxation time *versus* the inverse temperature for 2 (temperature range 1.8 to 9.2 K). Red to orange circles represent data extracted from ac magnetic susceptibility measurements (temperature range 3.6 to 9.2 K), and purple diamonds represent data extracted from dc relaxation experiments (temperature range 1.8 to 3.25 K). The black line represents a fit to an Orbach, a Raman, and a quantum tunnelling process. Individual contributions of the multiple magnetic relaxation pathways are shown in Fig. S30.

The steep drop in *χ*_M_*T* at low temperatures, and the divergence in zfc-/fc-cooled data revealed the presence of magnetic blocking in 2. To decipher the prevalent relaxation mechanisms, variable-frequency, variable-temperature in-phase (*χ*_M_′) and out-of-phase (*χ*_M_′′) alternating current (ac) magnetic susceptibility measurements with an oscillating field of 3 Oe were conducted. Peaks were monitored in *χ*_M_′′ for 2, suggestive of long magnetic relaxation times (Fig. 7). The extraction of magnetic relaxation times, *τ*, proceeded through generating *χ*_M_′′ *vs. χ*_M_′ plots for each temperature and fitting these Cole–Cole (Argand) plots to a generalised Debye function (Fig. S17). The relaxation times were employed in the construction of Arrhenius plots (Fig. 8, S29 and S30), where these allow the analysis of the temperature dependence of *τ*, uncovering what type of magnetic relaxation pathways are operational. Specifically, the presence of an activation barrier in terms of moment reversal translates into the system needing to exchange energy with the lattice (as phonons) to climb to the pinnacle of the barrier before relaxation can set in. This type of a relaxation mechanism is known as an Orbach process,^87,88^ and gives an exponential dependence of *τ* upon temperature.

**Fig. 9:**
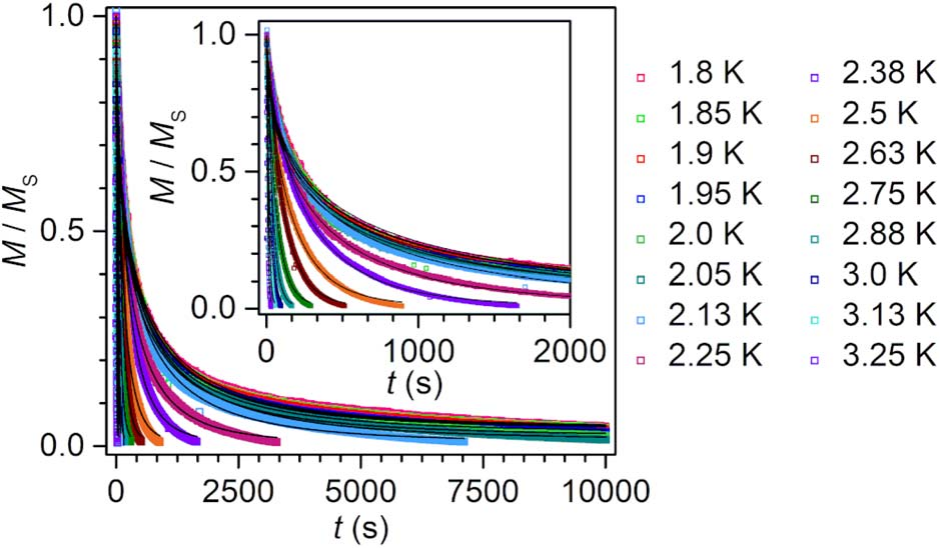
Plot of magnetisation (normalised) *vs.* time used to derive relaxation times for 2 at different temperatures from 1.8 to 3.25 K (inset: 2.38 to 3.25 K, shown separately for clarity). The temperatures were rounded to the second digit for improved readability. The data were fit (black line) to a function of the form *M*(*t*) = *M*_eq_ + (*M*_0_ − *M*_eq_) exp(−(*t*/*τ*)^*β*^) where *β* is a stretch factor. Decay of the magnetisation *vs.* time for 2 was obtained by applying a magnetic field of 7 T to the sample at a given temperature for 5 min and then by quickly removing the magnetic field. The full data (unnormalised) recorded at each temperature are shown in Fig. S21–S28.

Specifically, subjecting 2 to ac frequencies of 0.1 to 1000 Hz at temperatures from 3.6 to 9.2 K, and under the absence of a dc field, the *χ*_M_′′ signal maximum changed frequency over the entire investigated temperature range. The relaxation times are fully temperature dependent over the entire temperature regime, suggesting an operative thermally activated relaxation process, which is reflected in the perfect linearity of the Arrhenius plot (ln(*τ*) *vs.* 1/*T*) (Fig. S19). A fit to the Arrhenius expression, namely a single Orbach process according to *τ*^−1^ = *τ*_0_^−1^ exp(−*U*_eff_/*k*_B_*T*), afforded an effective energy barrier to spin relaxation *U*_eff_ of 36.00(8) cm^−1^ and attempt time *τ*_0_ of 9.8(2) × 10^−7^ s (Table 3). The low-temperature regime below 3.6 K is innate to very long relaxation times, and thus, is inaccessible through ac magnetic susceptibility methods. To gain insight into the magnetisation dynamics at those low temperatures, dc magnetic relaxation experiments were carried out (Fig. 9). In this sophisticated technique, the sample is first magnetically saturated by the application of a high, 7 T dc field at high temperature, then cooled under field towards the temperature at which the measurement should take place. Second, after proper thermalisation, the dc field is quickly removed, and the time-dependent decay of the magnetisation is recorded. The relaxation will follow an exponential dependence. For 2, dc relaxation experiments were performed between 1.80 and 3.25 K, and the relaxation times were obtained through fitting the decay curves to a stretched exponential function (Fig. S21–S28).

**Table 3:** Best-fit parameters for the Arrhenius plots of 2 under a 0 Oe applied dc field

Temperature (K)	3.6–9.2	1.8–9.2^a^	1.8–9.2^b^
*U*_eff_ (cm^−1^)	36.00(8)	36.2(1)	36.00
*τ*_0_ (s)	9.8(2) × 10^−7^	1.00(5) × 10^−6^	9.8 × 10^−7^
*C* (s^−1^ K^−*n*^)	—	8(4) × 10^−7^	4(2) × 10^−6^
*n*	—	8.9(4)	7.2(5)
*τ*_QTM_ (s)	—	447(10)	479(33)

a Orbach term freely refined.

b Orbach parameters fixed to values derived from ac magnetic susceptibility data.

The relaxation times obtained from both dc relaxation experiments and ac magnetic susceptibility measurements were used to construct the Arrhenius plot in Fig. 8. Taking into account all relaxation times, a clear deviation from linearity is observed at low temperatures, pointing at additional relaxation pathways at play. A satisfactory fit to all *τ* was ultimately attained by considering an Orbach, a Raman and a quantum tunnelling mechanism (QTM) according to eqn (2):

2 *τ*^−1^ = *τ*_0_^−1^ exp(−*U*_eff_/*k*_B_*T*) + *CT*^*n*^ + *τ*_QTM_^−1^

where the first term corresponds to an Orbach process, the second term to a Raman process with *C* and *n* as the Raman coefficients, and the third term reflects the QTM process with *τ*_QTM_^−1^ as the QTM rate. Thus, the fit of all *τ* yielded a spin-reversal barrier *U*_eff_ of 36.2(1) cm^−1^ with a pre-exponential factor *τ*_0_ of 1.00(5) × 10^−6^ s, Raman parameters of *C* = 8(4) × 10^−7^ s^−1^ K^−*n*^ and *n* = 8.9(4), and *τ*_QTM_ of 447(10) s (Fig. S29 and Table 3). QTM typically impacts the low temperature regime, however, the magnitude of *τ*_QTM_ implies the efficacy of Ln-radical coupling as a measure to attenuate QTM and thus, a slowdown of the magnetic relaxation. Notably, the *n* magnitude is rather large compared to other radical-bridged dilanthanide SMMs. Thus, we probed the possibility of using the values obtained from ac measurements to reduce over parameterisation (Fig. S30). This alternate fit afforded *C* = 4(2) × 10^−6^ s^−1^ K^−*n*^ and *n* = 7.2(5), and *τ*_QTM_ of 479(33) s which is more similar to values to other radical-bridged SMMs such as [(Cp*_2_Dy)_2_(μ-dmeotz˙)(THF)][BPh_4_] with *C* = 9.62 × 10^−3^ s^−1^ K^−*n*^ and *n* = 6.88 or [K(thf)_6_][(Cp*_2_Ln)_2_(μ-ind˙)] with *C* = 2.27 × 10^−3^ s^−1^ K^−*n*^ and *n* = 8.2 (dmeotz = 3,6-dimethoxy-1,2,4,5-tetrazine; ind = indigo).^19,24,89^

Taken together, the dynamic magnetic measurements provide evidence for the SMM behaviour of 2 with magnetic blocking occurring below 3.6 K.

For comparison, the dynamic magnetic behaviour for 1 was also explored (Fig. S15 and S16). Under frequencies ranging from 0.1 to 1000 Hz at temperatures from 5.5 to 21 K, the single *χ*_M_′′ peak changed frequency over the entire probed temperature range, suggestive of a thermally activated process.

The extracted relaxation times were fit to a Raman process, leading to an *C* = 3.3(5) × 10^−4^ s^−1^ K^−*n*^ and *n* = 5.34(6) (Fig. S18). The additional inclusion of an Orbach process did not afford a satisfactory fit.

### Field-dependent magnetisation measurements

**Fig. 10:**
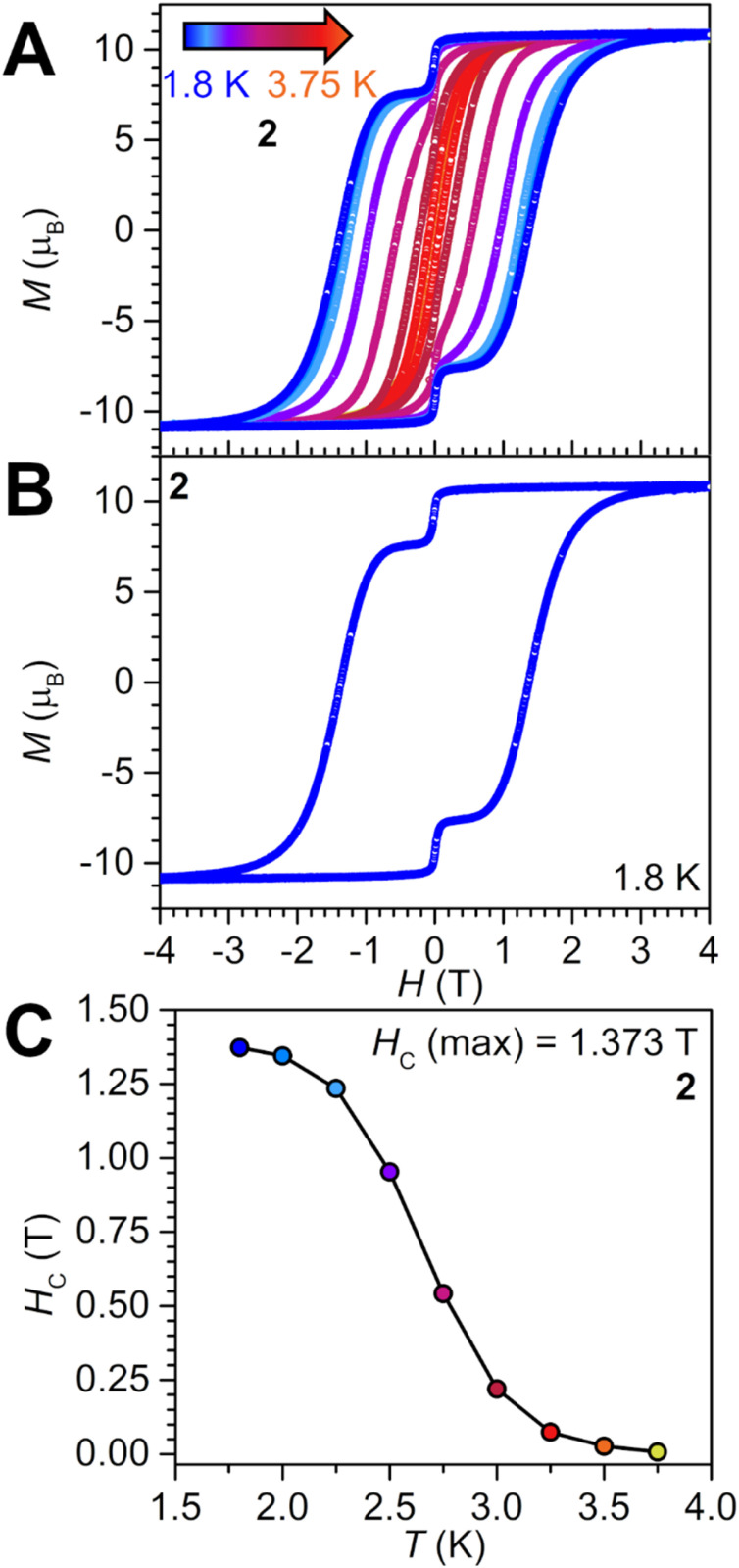
Plot of magnetisation (*M*) *vs.* dc magnetic field (*H*) at an average sweep rate of 0.01 T s^−1^ for 2: (A) from 1.8 to 3.75 K, and (B) at 1.8 K. (C) Plot of coercive field *vs.* temperature for 2, where solid line is a guide for the eye.

The employment of SMMs in storage applications requires a non-zero magnetisation under zero applied field. To probe the utility of 2 to this end, variable-field magnetisation measurements on a polycrystalline sample were carried out between ±7 T and from 1.8 to 3.75 K with an average sweep rate of 0.01 T s^−1^ (Fig. 10 and S33). The collected hysteresis loops are open below 3.75 K with a maximum coercive field *H*_C_ of 1.373 T at 1.8 K. This represents the highest *H*_C_ value found for any organic radical-bridged dinuclear SMM reported to date, where *H*_C_ has been at least doubled to tripled when compared to reported systems, taking into account conventional and somewhat akin sweep rates (Table 4): *H*_C_ is 0.6 T in [(Cp*_2_Dy)_2_(μ-bpym˙)][BPh_4_], 0.54 T in [K(crypt-222)][(Cp*_2_Dy)_2_(μ-Bbim˙)], 0.5 T in [(Cp*_2_Dy)_2_(μ-pyz˙)(THF)_2_][BPh_4_], and 0.6 T in [(Cp*_2_Dy)_2_(μ-dmtz˙)(THF)_2_][BPh_4_] (bpym = bipyrimidine, Bbim = 2,2′-bisbenzimidazole, pyz = pyrazine, dmtz = 3,6-dimethyl-1,2,4,5-tetrazine).^21,24,89^ Remarkably, the *H*_C_ value of 2 exceeds even that of SMMs that bear an inorganic radical, most notably an N_2_^3−^˙, such as in [K(crypt-222)][(Cp^Me_4_H^_2_Dy)_2_(μ-N_2_˙)] with an *H*_C_ of 1 T at 2 K (*H*_C_ is also 1 T at 5.5 K).^90^

**Table 4:** Selected examples of dinuclear radical-bridged dysprosium SMMs with coercive field (*H*_C_), hysteresis temperature (*T*_H_) and magnetic exchange coupling (*J*)

Compound	*H*_C_ (T)	*T*_H_ a (K)	*J* (cm^−1^)	Reference
[(Cp*_2_Dy)_2_(μ-bpym˙)][BPh_4_]	0.6	6.5	−10.0^b^	24
[K(crypt-222)][(Cp*_2_Dy)_2_(μ-Bbim˙)]	0.54	5.5	−1.96^b^	18
[(Cp*_2_Dy)_2_(μ-tppz˙)][BPh_4_]	0.1	3.25	−6.91(4)^b^	16
[(Cp*_2_Dy)_2_(μ-pyz˙)(THF)_2_][BPh_4_]	0.5	8	−22.2^b^	21
[(Cp*_2_Dy)_2_(μ-dmtz˙)(THF)_2_][BPh_4_]	0.6	3.4	−11.7^b^	89
[K(crypt-222)][(Cp^Me_4_H^_2_Dy)_2_(μ-N_2_˙)]	1	8	−20.0^b^	90
[(Cp*_2_Dy)_2_(μ-tan˙)][BArF_20_] (2)	1.373	3.75	∼ −24^c^	This work

a Signifies open magnetic hysteresis loops at or below this temperature.

b Determined from fitting of the dc susceptibility data of the corresponding Gd complexes.

c Determined from broken-symmetry DFT calculations. Abbreviations: bpym = 2,2′-bipyrimidine, Bbim = 2,2′-bisbenzimidazole, tppz = 2,3,5,6-tetra(2-pyridyl)pyrazine, pyz = pyrazine, dmtz = 3,6-dimethyl-1,2,4,5-tetrazine, tan = 1,4,5,8-tetraazanaphthalene. Employed sweep rates were conventional and varied between 20 and 100 Oe for the selected examples.

With rising temperatures in the variable-field magnetisation measurements of 2, *H*_C_ gradually decreases to first 0.953 T at 2.5 K, and then rapidly to 0.026 T at 3.5 K. Above the latter temperature, *H*_C_ further decreases and approaches the scan rate. Relative to the literature examples above, the hysteresis loops in 2 close at lower temperatures than for the bpym˙ (6.5 K), Bbim˙ (5.5 K) and pyz˙ (8 K) radical-bridged complexes, but close at a slightly higher temperature than the loops for the dmtz˙ complex (3.4 K).^21,89^

The hysteresis loops of 2 exhibit a prominent step at *H* = 0 T, hinting at ground state quantum tunnelling of the magnetisation. This is in line with the pronounced curvature of the Arrhenius plot below 3.25 K, Fig. 8.

Noteworthy, analogous variable-field magnetisation measurements conducted for 1 using the same sweep rate, produced hysteresis loops from 1.8 to 5 K, Fig. S31, which visual appearance is vastly different relative to 2. In fact, the loops are barely open, with the coercive field reading only 590 Oe at 1.8 K. The pronounced quantum tunnelling in 1 on the timescale of the hysteresis measurements, is attributed to the presence of two noninteracting Dy^III^ ions. The result also demonstrates the power of implementing a radical as an exchange medium in between lanthanide ions, such as in 2, to afford magnetic memory effect.

Isothermal field-dependent magnetisation measurements (*M vs. H*) for 2 were performed between 0 and 7 T and from 2 to 10 K (Fig. S35). At 2 K, the magnetisation curve exhibits a pronounced S-shape, associated with large magnetic anisotropy and magnetic blocking. Under an applied external magnetic field, *M* first raises to 0.17*μ*_B_ at 0.126 T, then plateaus at ∼0.700 T, before rapidly boosting to 10.07*μ*_B_ at ∼2.480 T. Subsequently, the ascent of *M* is slower until the maximum *M*_max_ value of 10.99*μ*_B_ at 7 T, albeit not reaching full magnetic saturation. This *M*_max_ is in excellent agreement with other radical-bridged dinuclear complexes such as 10.97*μ*_B_ for [K(thf)_6_][(Cp*_2_Dy)_2_(μ-ind˙)]·THF (where ind = indigo) and 11.25*μ*_B_ for [K(crypt-222)][(Cp*_2_Dy)_2_(μ-Bbim˙)], respectively.^18,19^ At higher temperatures, the S-shape disappears, and the *M vs. H* curves exhibit a continuous shape akin to paramagnetic compounds with negligible magnetic anisotropy. The reduced magnetisation curves (*H*/*T vs. M*) are non-superimposable at low temperatures but increasingly overlap at 6 K and above (Fig. S35). This is yet another indication for low-lying excited states originating from magnetic coupling between Dy^III^ and radical centres and/or crystal field splitting.

The occurrence of such a large coercive field while simultaneously possessing a modest hysteresis temperature appears counterintuitive at first, as one would assume a correlation of the two parameters. Although extremely rare, such observations were made for larger systems containing four metals such as [(Cp*_2_Dy)_4_(μ-pyz˙)_4_]·10THF with an *H*_c_ of 6.5 T, where the hysteresis closes at 9 K, which is on par with dinuclear systems.^21^ Other multimetallic comparative examples include [(Cp*_2_Dy)_3_(μ_3_-HAN˙)] (HAN = hexaazatrinaphthylene) with *H*_C_ = 0.8 T and open hysteresis loops below 3.5 K.^17^

Considering the canvassed bridging ligands and coordination geometries so far, there is no trend in the magnitude of *H*_C_ and hysteresis temperature detectable. This underlines the importance of exploring new radical systems accompanied by magneto-structural correlations towards a substantial improvement in performance of polynuclear lanthanide SMMs.

### Cauchy probability distribution function (CPDF) analysis

**Fig. 11:**
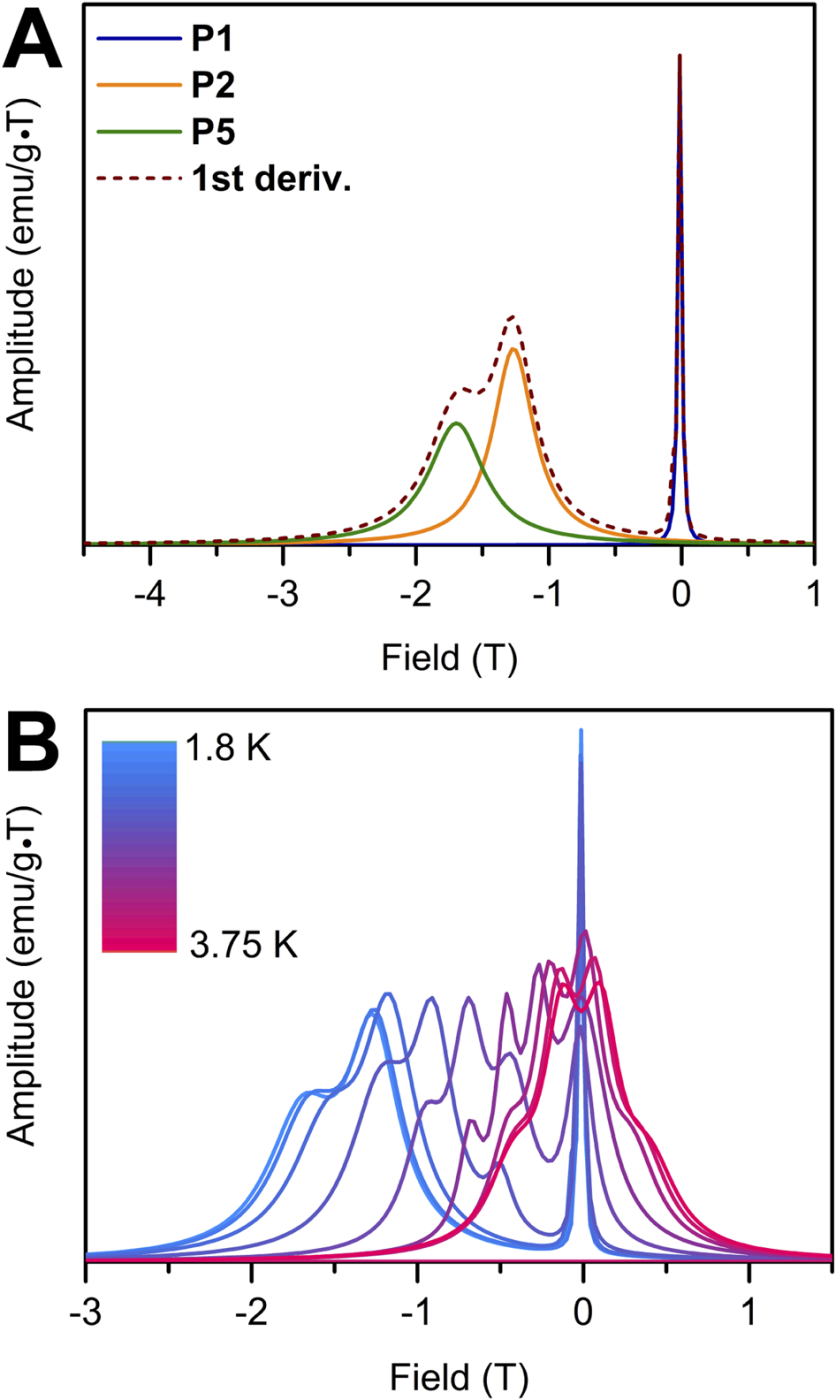
(A) First derivative of the reverse sweep of the magnetic hysteresis loop of 2 at 1.8 K is shown with a dashed line. P1, P2 and P5 are distinct demagnetisation processes uncovered through the Cauchy probability distribution function analysis. P3 and P4 processes are not observed at temperatures below 2.25 K. (B) First derivatives of the reverse sweep of the magnetic hysteresis loops of 2 collected from 1.8 K to 3.75 K.

Cauchy probability distribution function (CPDF) analysis^91^ was carried out on the reverse sweep of the magnetic hysteresis data of 2 to unveil traceable demagnetisation processes. A magnetic hysteresis loop can be reproduced through a summation of arctangent functions.^92^ The magnetic hysteresis loop data of 2 from 1.8 K to 3.75 K were fit using arctangent functions and the first derivative of each fit was attained, Fig. 11. The inflection points in the first derivative correspond to different demagnetisation processes that are buried within the magnetic hysteresis data. Since the first derivative of an arctangent function follows a Lorentzian distribution, it can be analysed using CPDF. This analysis provides information about the half-width at half maximum (*γ*), the field position, and the peak amplitude for each demagnetisation process.

Five different demagnetisation events (P1, P2, P3, P4 and P5) are observed for the hysteresis data of 2 from 1.8 K to 3.75 K, Fig. 11. Out of these, P1 is the only process observed throughout the entire temperature regime. P1 is mainly located at ∼0 T field and is very narrow (*γ* ∼0.02 T) up until 2.5 K (Table S3). As the temperature increases, P1 becomes broader (*γ* ∼ 0.2 T) and shifts towards positive field positions (0.12 T) at 3.75 K. Due to the narrowness and its position at 0 T, P1 is attributed to a QTM process contributing to magnetic relaxation. With increasing temperature, the percent contribution of P1 remains constant until 2.5 K (Fig. S37). Above this temperature, the percent contribution fluctuates. Since the P1 peak broadens and features temperature-dependent behaviour above 2.5 K, it can be deduced that at higher temperatures P1 does not constitute of a pure QTM process and thermally activated relaxation processes are coalescing to produce a unified demagnetisation process.

P2 is a broader process occurring at around −1.26 T at 1.8 K, which gradually shifts towards more positive field values. Beyond 2.25 K, P2 disappears, and two new processes (P3 and P4) originate. These are also broad, and they shift toward positive field values with higher temperatures (Fig. S36 and Table S3). P3 appears at 2.5 K and exhibits an upward trend in percent contribution until 3.75 K and P4 is only observed at temperatures from 2.5 K to 3.0 K. P5 is the demagnetisation process that occurs at the most negative field position of −1.69 T at 1.8 K and also gradually moves towards positive field values with rising temperature. The percent contribution from P5 gradually declines with increasing temperatures and reaches zero at 3.25 K, beyond which it is not observable.

In sum, the evaluation of the magnetic hysteresis data by means of CPDF, points at different processes contributing towards the demagnetisation of 2. At low temperatures, a QTM process is clearly identified at approximately 0 T field, whereas raising the temperature induces the origination of additional demagnetisation processes owing to thermally activated relaxation processes. At high temperatures, pure QTM processes are not observed, and different demagnetisation processes coalesce resulting in two processes.

### Derivation of magnetic coupling from density functional theory

The multiconfigurational nature of Dy^III^ ions renders commonly employed fitting methods of *χ*_M_*T vs. T* data as tools to derive magnetic coupling constants, *J*, inaccessible as the required crystal field parameters are usually unknown.

Computational methods such as broken-symmetry density functional theory are useful to derive *J* for isotropic systems such as Gd^III^ ions and organic radicals.^93^ Hence, the coupling in 2 can be approximated by calculating model complexes, 2^Gd^, with Dy substituted for Gd, and subsequent scaling of the obtained values to account for the larger magnetic moment of Dy.

To analyse the intramolecular coupling in the different conformers of 2, broken-symmetry DFT calculations were carried out on the atomic coordinates of 2 with Dy being substituted by Gd. These calculations were performed on the edge-centred molecular unit (M1) and the two disorder parts of the face-centred molecular unit (M2A and M2B). The exchange coupling constant (*J*) was determined through the Heisenberg–Dirac–van Vleck spin Hamiltonian (*Ĥ* = −2*J*·*S*_Dy_·*S*_Rad_) and *J* was calculated by *J* = −(*E*_HS_ − *E*_BS_)/(〈*S*^2^〉_HS_ − 〈*S*^2^〉_BS_) formalism. Here, *E*_HS_ and *E*_BS_ are the energies of the high spin and the broken-symmetry states, respectively, and 〈*S*^2^〉_HS_ and 〈*S*^2^〉_BS_ represent the spin expectation values of the high spin and broken-symmetry states.^93^

As anticipated for an organic radical-bridged complex, the spin density primarily resides on the Gd centres and on the bridging tan ligand (Fig. S38 and Table S4). All three molecular units exhibit similar, huge antiferromagnetic exchange coupling as evidenced by the exchange coupling constant values (*J*_Gd-rad_) obtained through DFT. The *J*_Gd-rad_ values are −17.7 cm^−1^, −16.6 cm^−1^, and −16.7 cm^−1^ for M1, M2A, and M2B, respectively, confirming an approximately 1 cm^−1^ difference.

The derived magnetic exchange coupling constants hint at extremely strong coupling in 2, even relative to other radical-bridged dinuclear Gd complexes such as *J*_Gd-rad_ = −10.8 cm^−1^ obtained for [(Cp*_2_Gd)_2_(μ-5,5′-F_2_bpym˙)][BPh_4_], or *J*_Gd-rad_ = −11.7 cm^−1^ for [(Cp*_2_Gd)_2_(μ-dmtz˙)(THF)_2_][BPh_4_]·THF.^89,94^ The average −17.0 cm^−1^ for 2^Gd^ is only surpassed by −22.2 cm^−1^ determined for the pyrazinyl-bridged [(Cp*_2_Gd)_2_(μ-pyz˙)(THF)_2_][BPh_4_],^21^ and highlights the immense potential of linear azaacenes to promote strong magnetic coupling.

The determined *J*_Gd-rad_ values for 2^Gd^ were rescaled to obtain the approximate *J*_Dy-rad_ value for the Dy complex 2. *J*_Dy-rad_ can be attained by multiplying *J*_Gd_ by 1.4.^15^ This results in *J*_Dy-rad_ values of ∼–25 cm^−1^ for M1 and ∼–23 cm^−1^ M2A, and M2B. These are DFT approximations of the real exchange coupling values, however, both sign and magnitude are in excellent agreement with the experimental finding of strong antiferromagnetic coupling, giving rise to a “giant spin” state and accompanied magnetic blocking.

## Conclusions

The dianionic ligand 1,4,5,8-tetraazanaphthalene (tan) was used to generate the first dinuclear lanthanide complex containing the tan^2−^ bridging ligand, [(Cp*_2_Dy)_2_(μ-tan)] (1). Complex 1 was chemically oxidised to yield [(Cp*_2_Dy)_2_(μ-tan˙)][BArF_20_] (2), consisting of the radical bridging ligand, tan^1−^˙. Notably, complex 2 represents the first coordination compound containing a tan radical with any metal ion. Both 1 and 2 show slow magnetic relaxation under zero applied dc field. Specifically, the magnetic characterisation of 2 revealed that the tan^1−^˙ ligand promotes strong antiferromagnetic exchange coupling with the highly anisotropic dysprosium ions. The arising “giant-spin” leads to SMM behaviour with open hysteresis loops. In fact, a maximum coercive field of 1.373 T is observed which sets a record for organic radical-bridged dinuclear SMMs. The hysteresis loops are open below 3.75 K, thus exceeding most dinuclear SMMs reported to date. The magnetic exchange coupling strength was estimated *via* broken-symmetry DFT calculations, revealing a massive coupling of ∼−24 cm^−1^, ranging among the highest determined for radical-bridged lanthanide complexes.

These foregoing results demonstrate that rational chemical design of the bridging ligand to adjust the ligand's reduction potential to the high negative Dy^III^/Dy^II^ redox potential pose an auspicious avenue for performance improvements of radical-bridged SMMs. The design of bridging ligands with L/L^−^˙ redox processes at even lower potentials are expected to drastically boost temperatures. Moreover, the construction of higher-nuclearity lanthanide-radical systems employing tan may amplify hard magnet properties further and lead to powerful magnetic materials.

## Author contributions

Florian Benner synthesised the complexes, conducted SCXRD, spectroscopy and magnetic measurements including interpretation. Saroshan Deshapriya conducted and interpreted TDDFT and broken-symmetry DFT calculations, and CPDF fits. Selvan Demir wrote the manuscript with the input from all authors, led the project and provided resources and support.

## Conflicts of interest

There are no conflicts to declare.

## Supplementary Material

SC-016-D5SC05358G-s001

SC-016-D5SC05358G-s002

## Data Availability

CCDC 2455536 (1) and 2455829 (2) contain the supplementary crystallographic data for this paper.^95*a*,*b*^ Supplementary information: all computational data, Python scripts for CPDF analysis, spectroscopic data, SI figures and tables, and detailed crystallographic information. See DOI: https://doi.org/10.1039/d5sc05358g.
