# Supplementary material for: A tetraazanaphthalene radical-bridged dysprosium single-molecule magnet with a large coercive field
Source: Chem Sci. 2025 Oct 6;16(44):20806–22. doi: 10.1039/d5sc05358g (PMC12498250; doi:10.1039/d5sc05358g)
Supplement: SC-016-D5SC05358G-s001 [file SC-016-D5SC05358G-s001.pdf]

Supplementary Information (SI) for Chemical Science.
This journal is © The Royal Society of Chemistry 2025

Supplementary Information

for

A tetraazanaphthalene radical-bridged dysprosium single-molecule magnet with a large coercive field

Florian Benner, Saroshan Deshapriya and Selvan Demir*

Department of Chemistry, Michigan State University, East Lansing, Michigan 48824, United States

*Email: sdemir@chemistry.msu.edu (S.D.)

Table of Contents

1 Single Crystal X-ray Diffraction **S4**

Table S1. Crystallographic data and structural refinement of [(Cp*$_2$Dy)$_2$(μ-tan)], **1**, and [(Cp*$_2$Dy)$_2$(μ-tan$^•$)][BArF$_{20}$], **2**. S4
Figure S1. Unit cell of [(Cp*$_2$Dy)$_2$(μ-tan)], **1**, with highlighted π-π-stacking interactions. S5
Figure S2. Excerpt of the unit cell of [(Cp*$_2$Dy)$_2$(μ-tan)], **1**, with selected highlighted π-stacking interactions. S6
Figure S3. Excerpt of the unit cell of [(Cp*$_2$Dy)$_2$(μ-tan)], **1**, with selected highlighted π-stacking interactions. S7
Figure S4. Unit cell of [(Cp*$_2$Dy)$_2$(μ-tan$^•$)][BArF$_{20}$], **2**, with highlighted π-π-stacking interactions. S8
Figure S5. Excerpt of the unit cell of [(Cp*$_2$Dy)$_2$(μ-tan$^•$)][BArF$_{20}$], **2**, containing edge-centred molecules of **2** only. S9
Figure S6. Excerpt of the unit cell of [(Cp*$_2$Dy)$_2$(μ-tan$^•$)][BArF$_{20}$], **2**, containing face-centred molecules of **2** only. S10

2 IR Spectroscopy **S11**

Figure S7. FTIR spectra of [(Cp*$_2$Dy)$_2$(μ-tan)], **1**, and [(Cp*$_2$Dy)$_2$(μ-tan$^•$)][BArF$_{20}$], **2**. S11

3 UV-Vis Spectroscopy **S12**

Figure S8. UV-Vis spectrum of [(Cp*$_2$Dy)$_2$(μ-tan)], **1**. S12
Figure S9. UV-Vis spectra of [(Cp*$_2$Dy)$_2$(μ-tan$^•$)][BArF$_{20}$], **2**. S12

4 Cyclic Voltammetry **S13**

Figure S10. Full cyclic voltammogram of [(Cp*$_2$Dy)$_2$(μ-tan)], **1**. S13
Figure S11. Full cyclic voltammogram of [(Cp*$_2$Dy)$_2$(μ-tan$^•$)][BArF$_{20}$], **2**. S13

5 Magnetic Data **S14**

Figure S12. Variable-temperature dc magnetic susceptibility data of **1**, collected under 0.1 T and 1.0 T applied dc fields. S14
Figure S13. Variable-temperature dc magnetic susceptibility data of **2** collected under 1.0 T applied dc field. S14
Figure S14. Variable-temperature dc magnetic susceptibility data **2**, collected under 0.1 T and 1.0 T applied dc fields. S15
Figure S15. Variable-temperature, variable-frequency in-phase (χ_M', top) and out-of-phase (χ_M'', bottom) ac magnetic susceptibility data collected under a zero Oe applied dc field for **1**. S15
Figure S16. Cole-Cole (Argand) plots for ac susceptibility collected under zero applied dc field for [(Cp*$_2$Dy)$_2$(μ-tan)], **1**. S16
Figure S17. Cole-Cole (Argand) plots for ac susceptibility collected under zero applied dc field for [(Cp*$_2$Dy)$_2$(μ-tan$^•$)][BArF$_{20}$], **2**. S17

Figure S18. Arrhenius plot of the natural log of the relaxation time, τ, versus the inverse temperature obtained from ac measurements, for [(Cp*$_2$Dy)$_2$(μ-tan)], **1**. S17
Figure S19. Arrhenius plot of the natural log of the relaxation time, τ, versus the inverse temperature obtained from ac measurements, for [(Cp*$_2$Dy)$_2$(μ-tan$^\bullet$)][BArF$_{20}$], **2**. S18
Figure S20. Arrhenius plot of the natural log of the relaxation time, τ, versus the inverse temperature obtained from ac measurements at zero field, for **1** and **2**. S18
Figure S21. Plot of magnetisation vs. time used to derive relaxation times for [(Cp*$_2$Dy)$_2$(μ-tan$^\bullet$)][BArF$_{20}$], **2**, at 1.8 K and 1.85 K. S19
Figure S22. Plot of magnetisation vs. time used to derive relaxation times for [(Cp*$_2$Dy)$_2$(μ-tan$^\bullet$)][BArF$_{20}$], **2**, at 1.9 K and 1.95 K. S19
Figure S23. Plot of magnetisation vs. time used to derive relaxation times for [(Cp*$_2$Dy)$_2$(μ-tan$^\bullet$)][BArF$_{20}$], **2**, at 2.0 K and 2.05 K. S20
Figure S24. Plot of magnetisation vs. time used to derive relaxation times for [(Cp*$_2$Dy)$_2$(μ-tan$^\bullet$)][BArF$_{20}$], **2**, at 2.125 K and 2.25 K. S20
Figure S25. Plot of magnetisation vs. time used to derive relaxation times for [(Cp*$_2$Dy)$_2$(μ-tan$^\bullet$)][BArF$_{20}$], **2**, at 2.375 K and 2.5 K. S21
Figure S26. Plot of magnetisation vs. time used to derive relaxation times for [(Cp*$_2$Dy)$_2$(μ-tan$^\bullet$)][BArF$_{20}$], **2**, at 2.625 K and 2.75 K. S21
Figure S27. Plot of magnetisation vs. time used to derive relaxation times for [(Cp*$_2$Dy)$_2$(μ-tan$^\bullet$)][BArF$_{20}$], **2**, at 2.875 K and 3.0 K. S22
Figure S28. Plot of magnetisation vs. time used to derive relaxation times for [(Cp*$_2$Dy)$_2$(μ-tan$^\bullet$)][BArF$_{20}$], **2**, at 3.125 K and 3.25 K. S22
Figure S29. Individual contributions of the multiple magnetic relaxation pathways to the Arrhenius plot of [(Cp*$_2$Dy)$_2$(μ-tan$^\bullet$)][BArF$_{20}$], **2**, with an Orbach, a Raman and a tunnelling process. S23
Figure S30. Individual contributions of the multiple magnetic relaxation pathways to the Arrhenius plot of [(Cp*$_2$Dy)$_2$(μ-tan$^\bullet$)][BArF$_{20}$], **2**, with an Orbach, a Raman and a tunnelling process. Orbach parameters were fixed to the values obtained from ac magnetic susceptibility. S24
Table S2. Relaxation times of **1** and **2** at various temperatures. S25
Figure S31. Plot of magnetisation (*M*) vs dc magnetic field (*H*) at an average sweep rate of 0.01 T/s for [(Cp*$_2$Dy)$_2$(μ-tan)], **1**, from 1.8 K to 5.0 K between ±0.45 T. S26
Figure S32. Plot of magnetisation (*M*) vs dc magnetic field (*H*) at an average sweep rate of 0.01 T/s for [(Cp*$_2$Dy)$_2$(μ-tan)], **1**, at 1.8 K between ±0.45 T. S26
Figure S33. Plot of magnetisation (*M*) vs dc magnetic field (*H*) at an average sweep rate of 0.01 T/s for [(Cp*$_2$Dy)$_2$(μ-tan$^\bullet$)][BArF$_{20}$], **2**, at 1.8 – 3.75 K between ±3 T. S27
Figure S34. Variable-temperature field-dependent magnetisation curves and reduced magnetisation data for **1**, collected from 0 to 7 T at 2, 4, 6, 8, and 10 K. S28

Figure S35. Variable-temperature field-dependent magnetisation curves and reduced magnetisation data for **2**, collected from 0 to 7 T at 2, 4, 6, 8, and 10 K. S28
Figure S36. Temperature dependence of the five demagnetisation processes identified in the first derivative of the magnetic hysteresis of $[(Cp^*{}_2Dy)_2(\mu\text{-tan}^{\bullet})][BArF_{20}]$, **2**. S29
Figure S37. Temperature dependence of the percent contributions of the five demagnetisation processes identified in the first derivative of the magnetic hysteresis of $[(Cp^*{}_2Dy)_2(\mu\text{-tan}^{\bullet})][BArF_{20}]$. S30
Table S3. Cauchy probability distribution function analysis data of the five demagnetisation processes determined in the first derivative of magnetic hysteresis of $[(Cp^*{}_2Dy)_2(\mu\text{-tan}^{\bullet})][BArF_{20}]$, **2**. S31

6 DFT Calculations S33

Table S4. Exchange coupling constant values obtained from the broken-symmetry DFT calculations performed on the model system of $[(Cp^*{}_2Gd)_2(\mu\text{-tan}^{\bullet})]^+$. S33
Figure S38. Spin density plot generated for the model system of $[(Cp^*{}_2Gd)_2(\mu\text{-tan}^{\bullet})]^+$ in $\mathbf{2}^{\mathbf{Gd}}$. S33
Table S5. TD-DFT calculated transition states of $[(Cp^*{}_2Dy)_2(\mu\text{-tan})]$, **1**. S34
Table S6. TD-DFT calculated transition states of $[(Cp^*{}_2Dy)_2(\mu\text{-tan}^{\bullet})][BArF_{20}]$, **2**. S36

7 Python script for fitting the first derivative of magnetic hysteresis data S38

8 Python script for the Cauchy probability distribution function analysis of magnetic hysteresis data S43

1 Single Crystal X-ray Diffraction

Table S1. Crystallographic data and structural refinement of [(Cp*$_2$Dy)$_2$(μ-tan)], **1**, and [(Cp*$_2$Dy)$_2$(μ-tan$^{•}$)][BArF$_{20}$], **2**.

Compound	**1**	**2**
CCDC no.	2455536	2455829
Empirical formula	$C_{46}H_{64}Dy_2N_4$	$C_{70}H_{64}BDy_2F_{20}N_4$
Formula weight	998.01	1677.06
Temperature/K	99.99(10)	99.99(10)
Crystal system	monoclinic	triclinic
Space group	$P2_1/c$	$P-1$
a (Å)	12.46040(10)	10.56700(10)
b (Å)	11.55780(10)	12.64630(10)
c (Å)	15.47030(10)	25.5268(2)
α (°)	90	77.5000(10)
β (°)	107.6150(10)	78.9540(10)
γ (°)	90	83.5720(10)
Volume (Å^3)	2123.49(3)	3259.82(5)
Z	2	2
ρ_{calc} (g/cm^3)	1.561	1.709
μ (mm^{-1})	18.862	13.033
F(000)	1000.0	1658.0
Crystal size (mm^3)	0.08 × 0.07 × 0.044	0.217 × 0.125 × 0.04
Radiation	CuK$_\alpha$ (λ = 1.54184)	CuK$_\alpha$ (λ = 1.54184)
2Θ range for data collection (°)	7.444 to 160.518	7.18 to 160.852
Index ranges	$-15 \leq h \leq 15$, $-14 \leq k \leq 13$, $-19 \leq l \leq 19$	$-13 \leq h \leq 13$, $-16 \leq k \leq 16$, $-28 \leq l \leq 32$
Reflections collected	40102	71904
Independent reflections	4631 [R_{int} = 0.0391, R_σ = 0.0245]	14063 [R_{int} = 0.0459, R_σ = 0.0340]
Data/restraints/ parameters	4631/489/437	14063/624/1086
Goodness-of-fit on F^2	1.097	1.058
Final R indexes (I > = 2 σ (I))	R_1 = 0.0270, wR_2 = 0.0635	R_1 = 0.0383, wR_2 = 0.0974
Final R indexes (all data)	R_1 = 0.0288, wR_2 = 0.0643	R_1 = 0.0410, wR_2 = 0.0991
Largest diff. peak/hole (e Å^{-3})	0.45/-1.43	1.48/-2.02

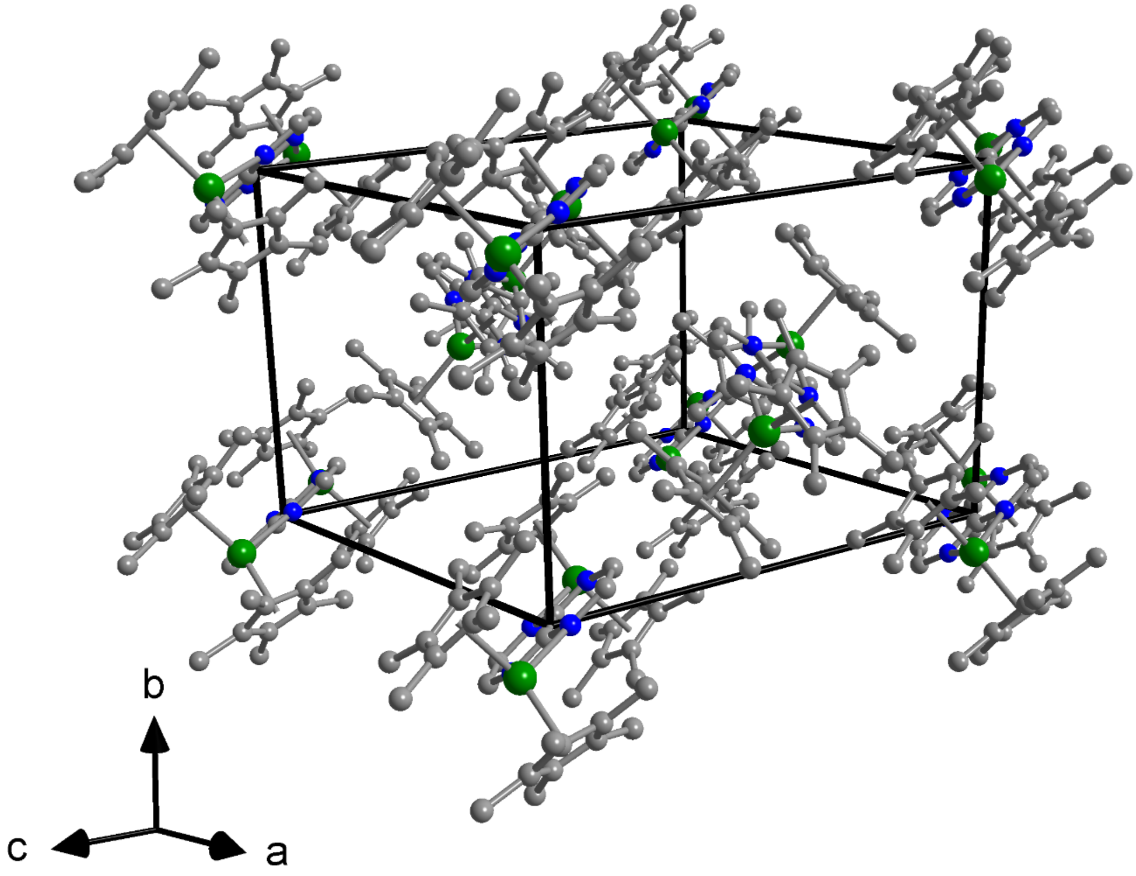

Figure S1. Unit cell of [(Cp*$_2$Dy)$_2$(μ-tan)], **1**. Dark green, blue, and grey spheres represent dysprosium, nitrogen, and carbon atoms, respectively. All hydrogen atoms are omitted for clarity.

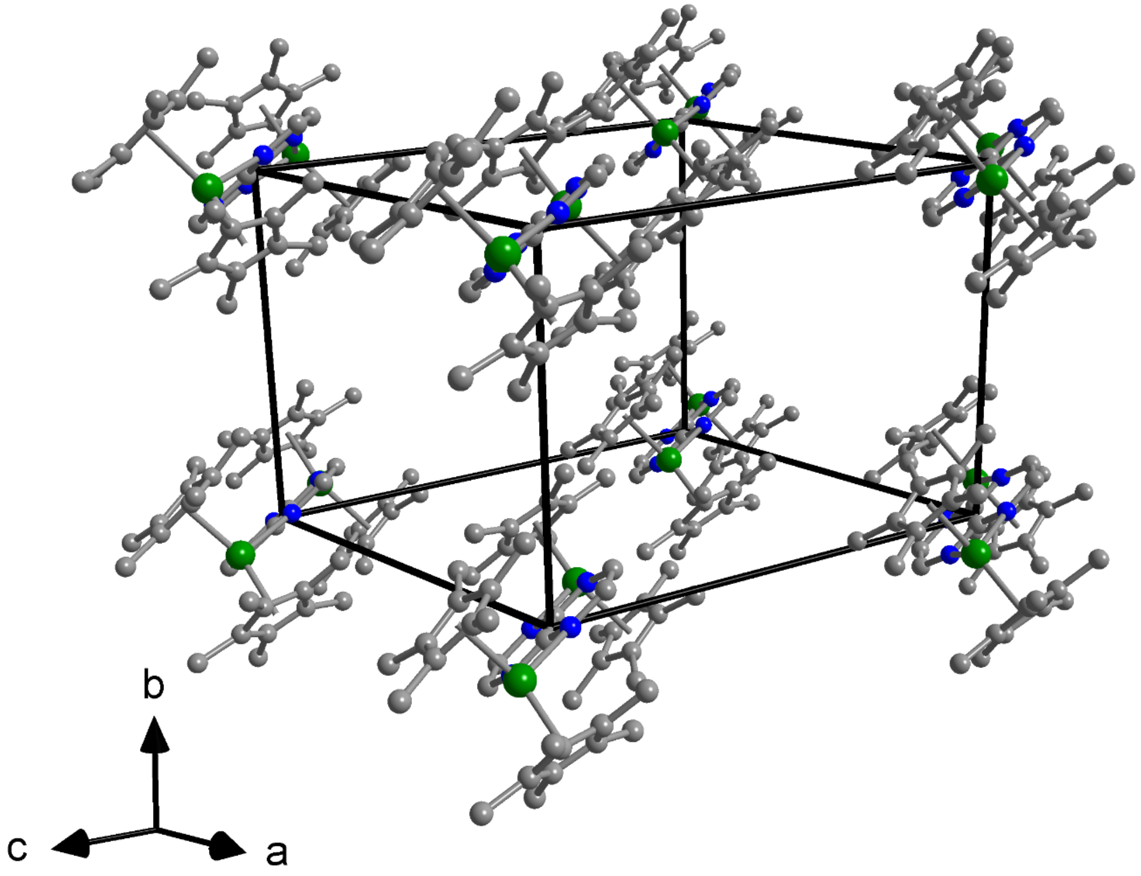

Figure S2. Excerpt of the unit cell of [(Cp*$_2$Dy)$_2$(μ-tan)], **1**, containing edge-centred molecules of **1** only. Dark green, blue, and grey spheres represent dysprosium, nitrogen, and carbon atoms, respectively. All hydrogen atoms and face-centred molecules were omitted for clarity.

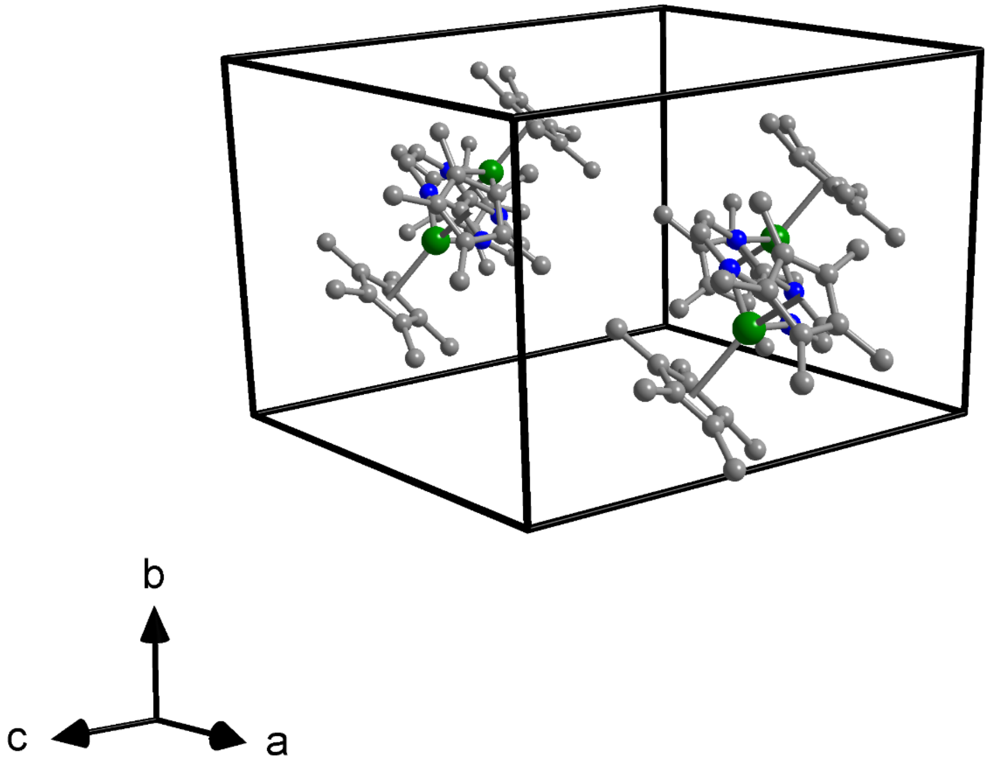

Figure S3. Excerpt of the unit cell of [(Cp*$_2$Dy)$_2$(μ-tan)], **1**, containing face-centred molecules of **1** only. Dark green, blue, and grey spheres represent dysprosium, nitrogen, and carbon atoms, respectively. All hydrogen atoms and edge-centred molecules were omitted for clarity.

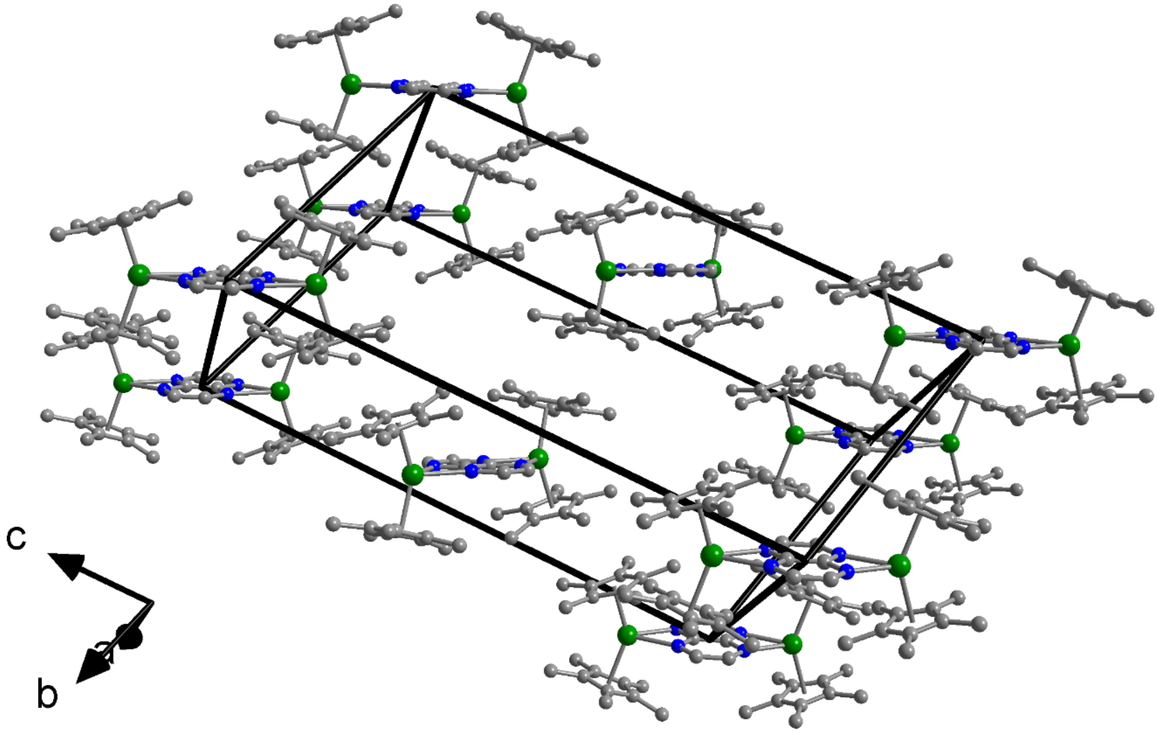

Figure S4. Unit cell of $[(Cp^*_2Dy)_2(\mu\text{-tan}^{\bullet})][BArF_{20}]$, **2**. Dark green, blue, and grey spheres represent dysprosium, nitrogen, and carbon atoms, respectively. All hydrogen atoms and $BArF^-$ counter anions are omitted for clarity.

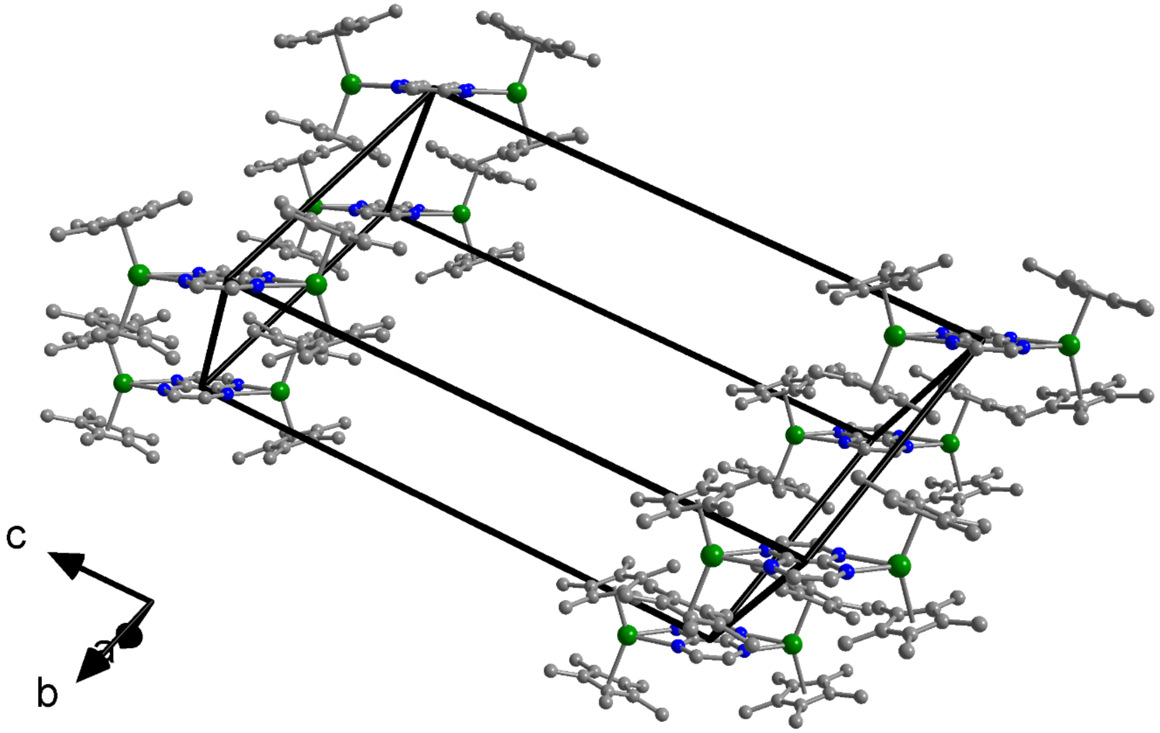

Figure S5. Excerpt of the unit cell of [(Cp*$_2$Dy)$_2$(μ-tan$^{•}$)][BArF$_{20}$], **2,** containing edge-centred molecules of **2** only (denoted M1 in the main text). Dark green, blue, and grey spheres represent dysprosium, nitrogen, and carbon atoms, respectively. All hydrogen atoms, BArF^{-} counter anions and face-centred molecules were omitted for clarity.

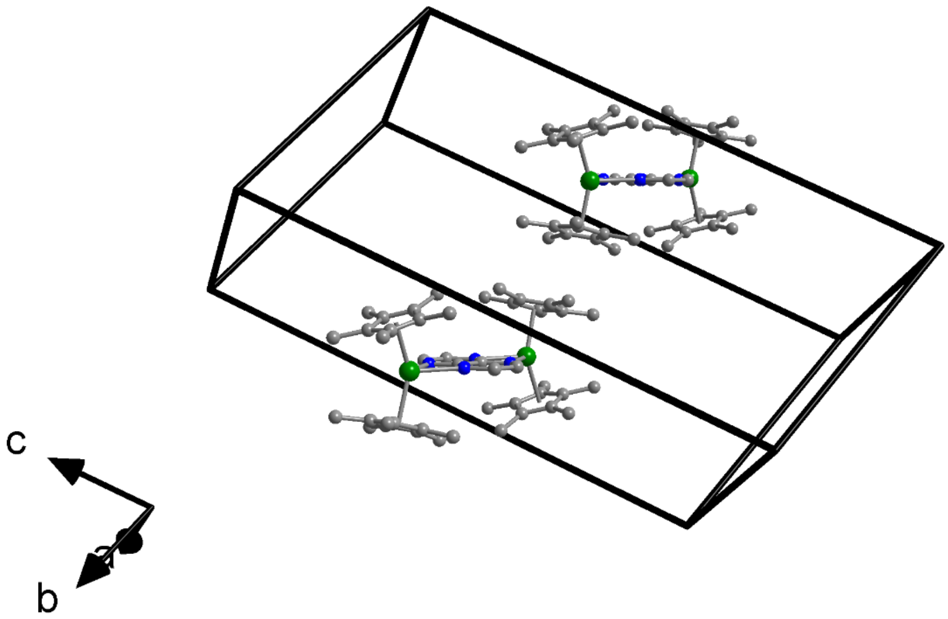

Figure S6. Excerpt of the unit cell of $[(Cp^*{}_2Dy)_2(\mu\text{-tan}^{\bullet})][BArF_{20}]$, **2**, containing face-centred molecules of **2** only (denoted M2 in the main text). Dark green, blue, and grey spheres represent dysprosium, nitrogen, and carbon atoms, respectively. All hydrogen atoms, $BArF^-$ counter anions and edge-centred molecules were omitted for clarity.

2 IR Spectroscopy

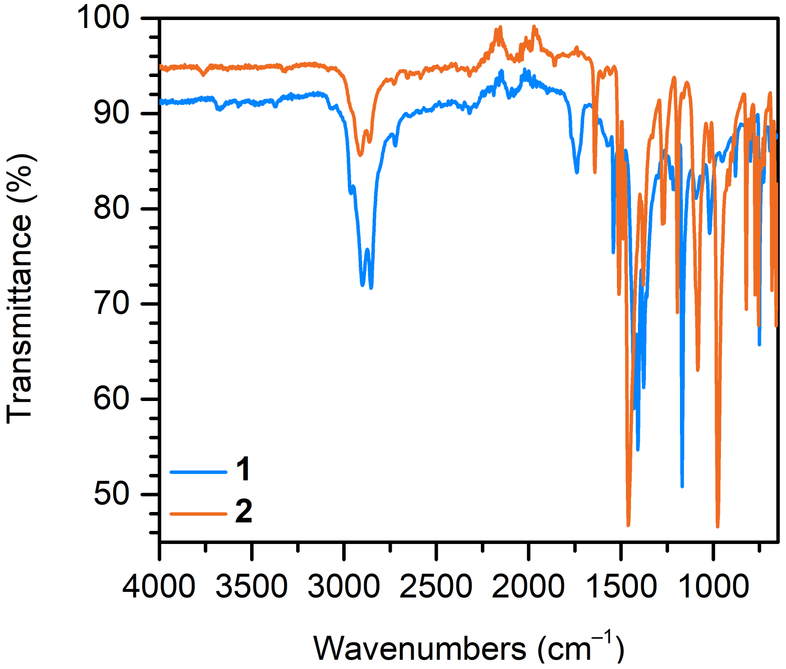

Figure S7. FTIR spectra of [(Cp*$_2$Dy)$_2$(μ-tan)], (**1**, blue line) and [(Cp*$_2$Dy)$_2$(μ-tan$^•$)][BArF$_{20}$], (**2**, orange line), collected on crushed crystalline solids under a nitrogen atmosphere.

3 UV-Vis Spectroscopy

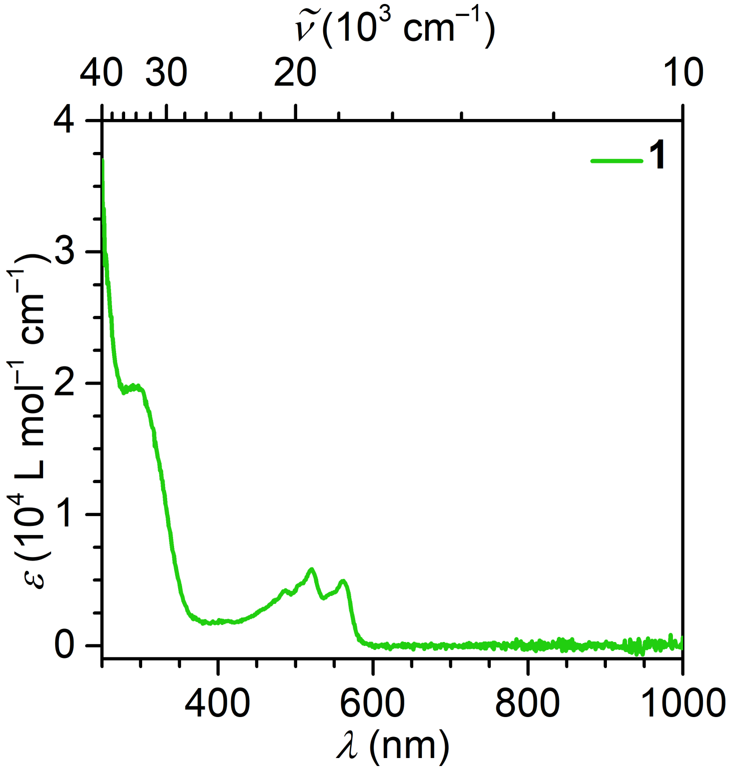

Figure S8. UV-Vis spectrum of [(Cp*$_2$Dy)$_2$(μ-tan)], (**1**), recorded at a 32 μmol/L concentration in DCM at room temperature.

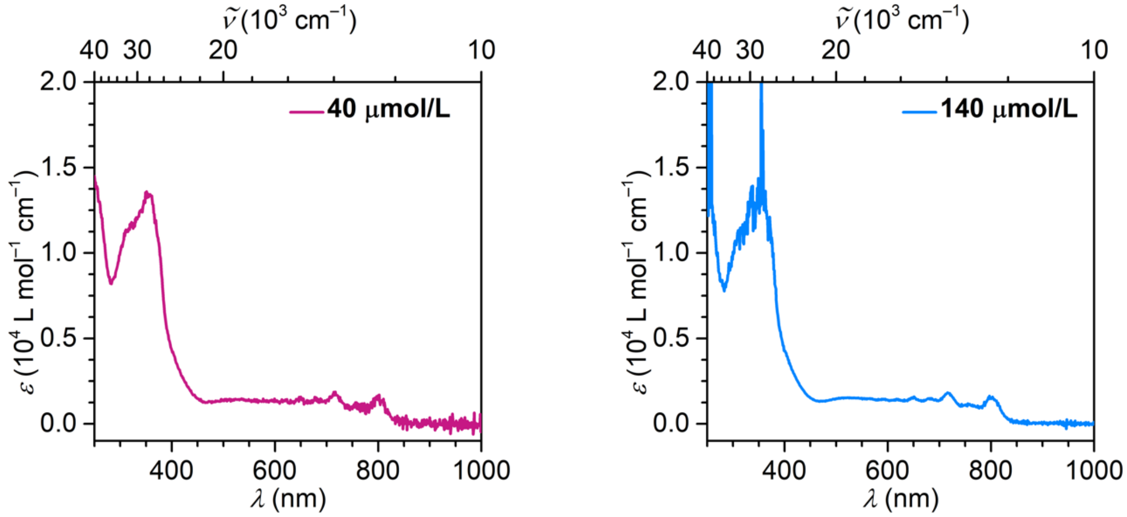

Figure S9. UV-Vis spectra of [(Cp*$_2$Dy)$_2$(μ-tan$^•$)][BArF$_{20}$], (**2**), recorded at 40 μmol/L (left), and 140 μmol/L (right) concentrations in DCM at room temperature.

4 Cyclic Voltammetry

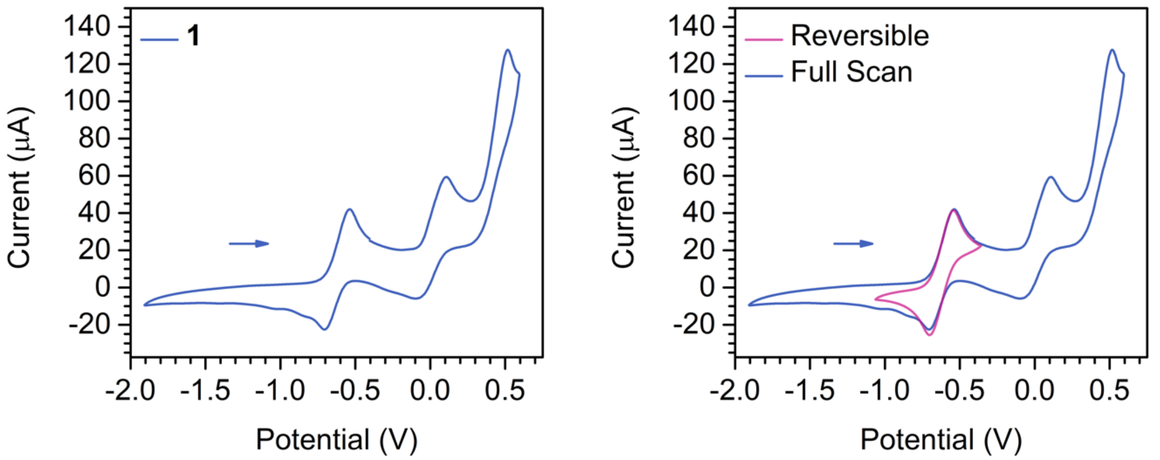

Figure S10. Full cyclic voltammogram of $[(Cp^*_2Dy)_2(\mu\text{-tan})]$, **1**, measured in dichloromethane (left). Full scan superimposed with reversible scan, shown in the main text (right).

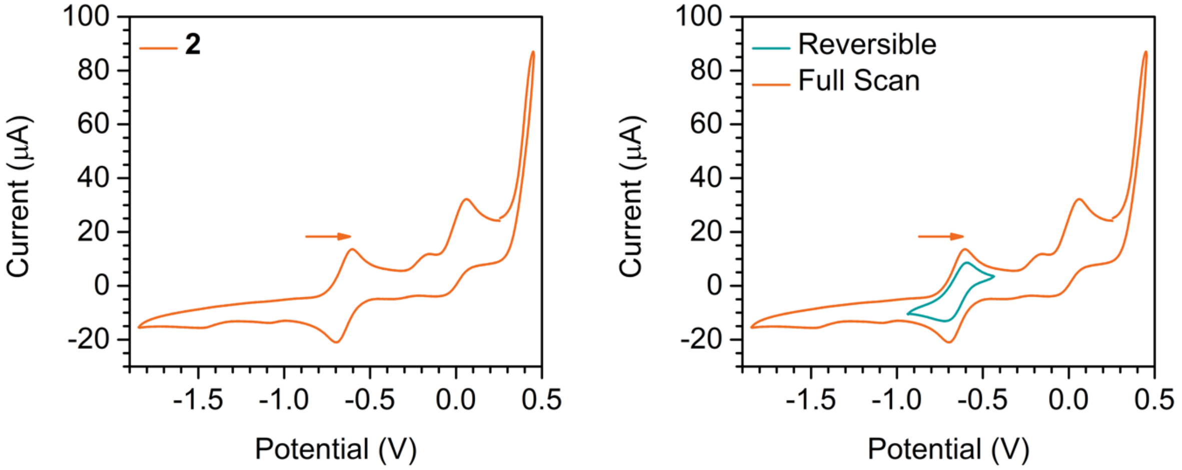

Figure S11. Full cyclic voltammogram of $[(Cp^*_2Dy)_2(\mu\text{-tan}^\bullet)][BArF_{20}]$, **2**, measured in dichloromethane (left). Full scan superimposed with reversible scan (teal), shown in the main text (right).

5 Magnetic Data

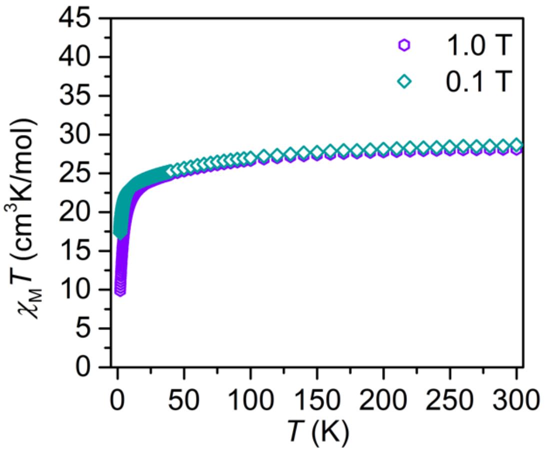

Figure S12. Variable-temperature dc magnetic susceptibility data for a restrained polycrystalline sample of $[(Cp^*{}_2Dy)_2(\mu\text{-tan})]$, **1**, collected under 0.1 T (turquoise hexagons), and 1.0 T (purple diamonds) applied dc fields.

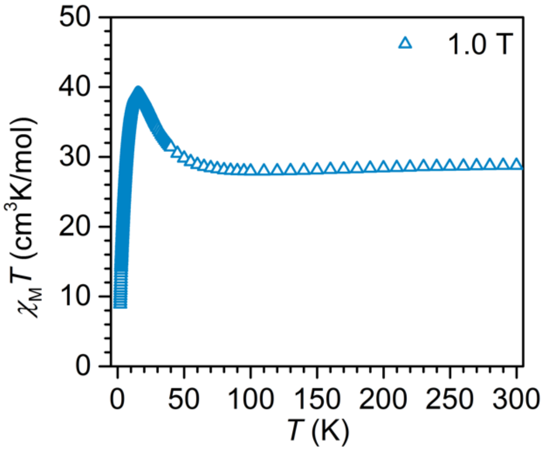

Figure S13. Variable-temperature dc magnetic susceptibility data for a restrained polycrystalline sample of $[(Cp^*{}_2Dy)_2(\mu\text{-tan}^{\bullet})][BArF_{20}]$, **2** collected under 1.0 T applied dc field.

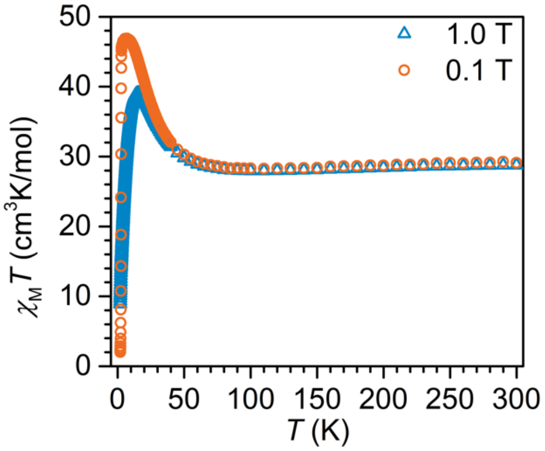

Figure S14. Variable-temperature dc magnetic susceptibility data for a restrained polycrystalline sample of [(Cp*$_2$Dy)$_2$(μ-tan$^\bullet$)][BArF$_{20}$], **2**, collected under 0.1 T (orange circles), and 1.0 T (blue triangles) applied dc fields.

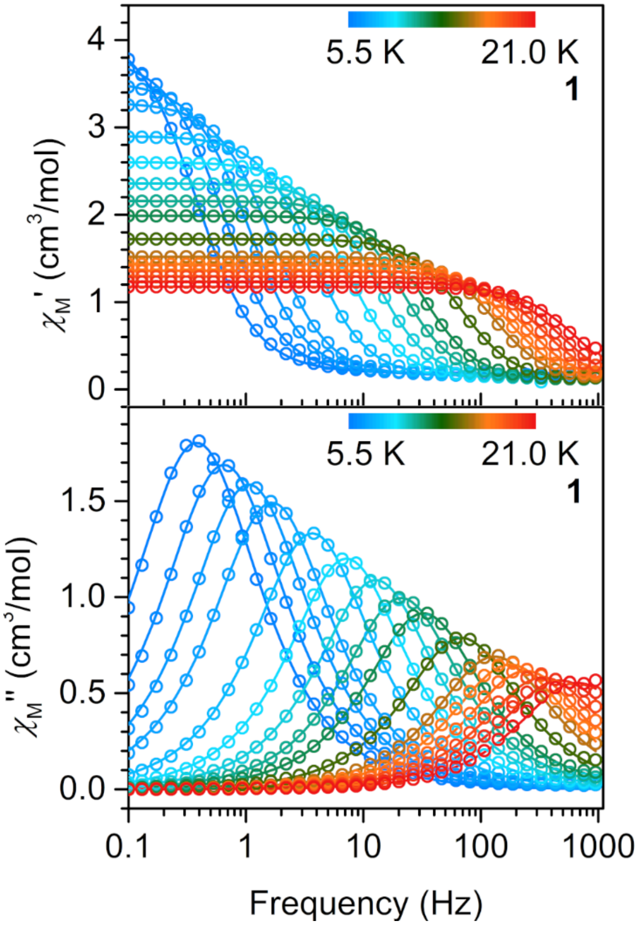

Figure S15. Variable-temperature, variable-frequency in-phase (χ_M', top) and out-of-phase (χ_M'', bottom) ac magnetic susceptibility data collected under a zero Oe applied dc field for **1**, from 5.5 to 21.0 K. Solid lines indicate the fits to a generalised Debye model.

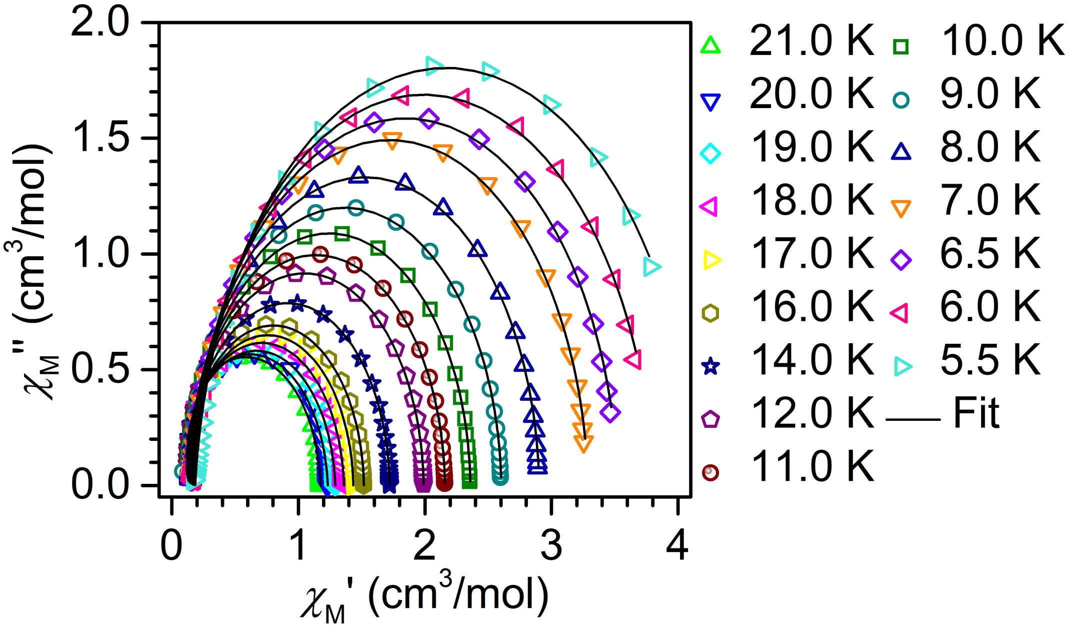

Figure S16. Cole-Cole (Argand) plots for ac susceptibility collected from 5.5 to 21.0 K under zero applied dc field for $[(Cp^*{}_2Dy)_2(\mu\text{-tan})]$, **1**. Symbols represent the experimental data points and the points representing the fits are connected by black solid lines.

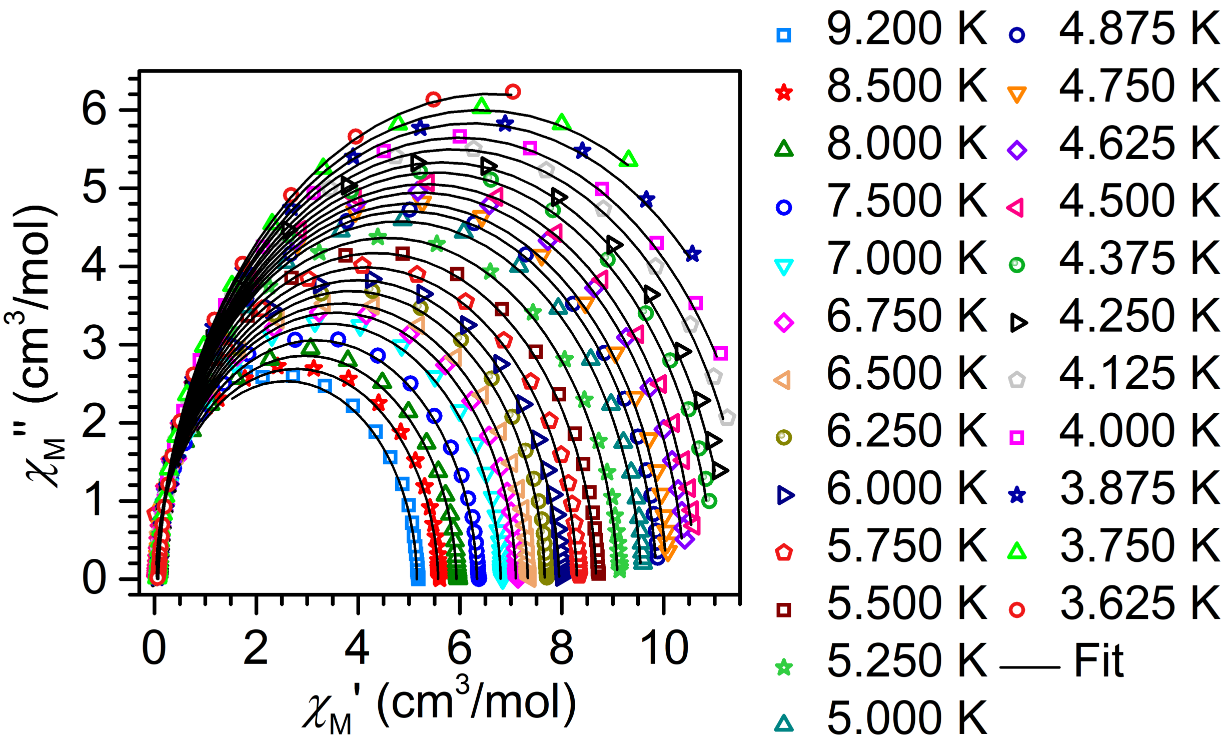

Figure S17. Cole-Cole (Argand) plots for ac susceptibility collected from 3.625 to 9.200 K under zero applied dc field for $[(Cp^*{}_2Dy)_2(\mu\text{-tan}^\bullet)][BArF_{20}]$, **2**. Symbols represent the experimental data points and the points representing the fits are connected by black solid lines.

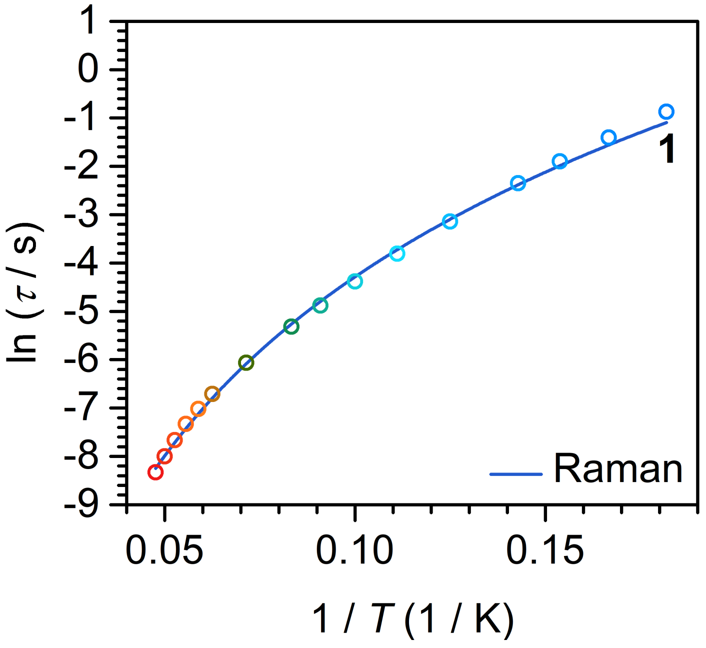

Figure S18. Arrhenius plot of the natural log of the relaxation time, τ, versus the inverse temperature obtained from ac measurements, for $[(Cp^*{}_2Dy)_2(\mu\text{-tan})]$, **1**, at 0 Oe from 5.5 to 21.0 K (blue to red circles). The dark blue line represents a fit to a Raman relaxation process yielding $C = 3.3(5) \times 10^{-4}$ s^{-1}K^{-n} and $n = 5.34(6)$.

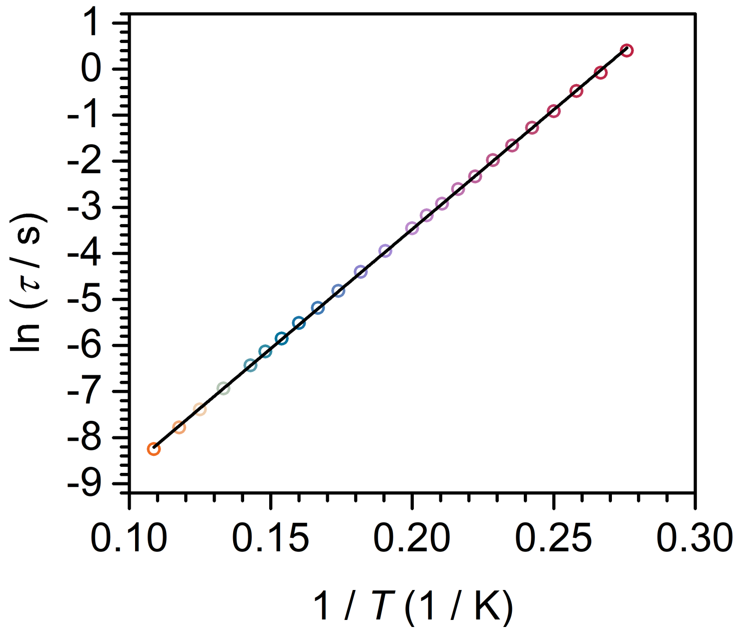

Figure S19. Arrhenius plot of the natural log of the relaxation time, τ, versus the inverse temperature obtained from ac measurements, for [(Cp*$_2$Dy)$_2$(μ-tan$^{\bullet}$)][BArF$_{20}$], **2**, at 0 Oe from 3.6 to 9.2 K (red to orange circles). The black line represents a fit to an Orbach relaxation process yielding U_{eff} = 36.00(8) cm^{-1} and τ_0 = 9.8(2) x 10^{-7} s.

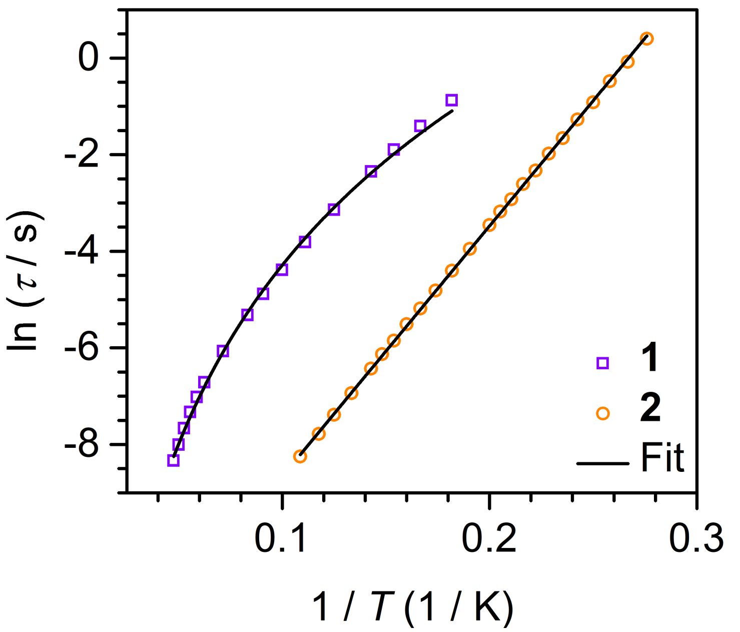

Figure S20. Arrhenius plot of the natural log of the relaxation time, τ, versus the inverse temperature obtained from ac measurements at zero field, for [(Cp*$_2$Dy)$_2$(μ-tan)], **1**, from 5.5 to 21.0 K (purple squares), and for [(Cp*$_2$Dy)$_2$(μ-tan$^{\bullet}$)][BArF$_{20}$], **2**, from 3.6 to 9.2 K (orange circles). See Figures S18 and S19 for fitting details.

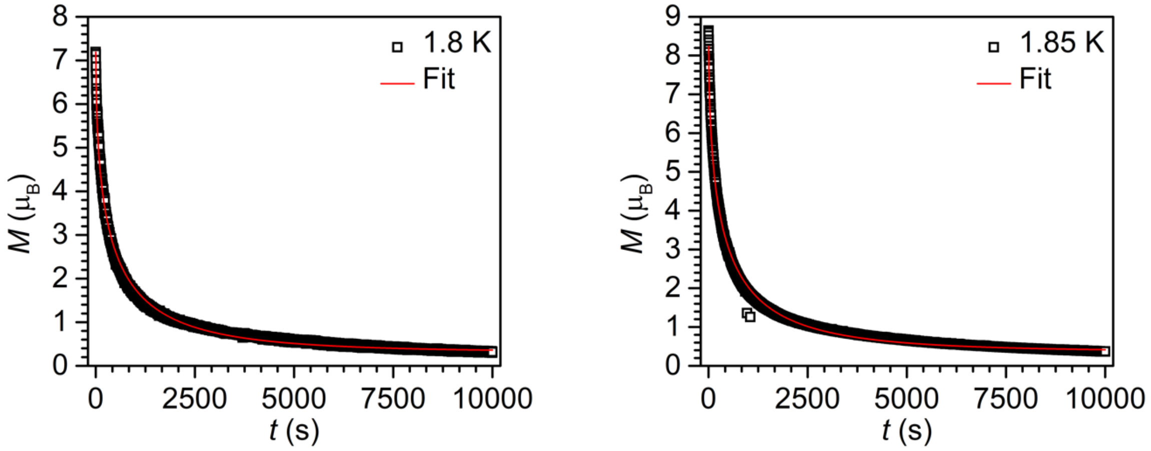

Figure S21. Plot of magnetisation vs. time used to derive relaxation times for $[(Cp^*{}_2Dy)_2(\mu\text{-}tan^{\bullet})][BArF_{20}]$, **2**, at 1.8 K (left) and 1.85 K (right). The data (black squares) were fit (red line) to a function of the form $M(t) = M_{eq} + (M_0 - M_{eq}) \exp\left(-\left(\frac{t}{\tau*}\right)^{\beta}\right)$ where β is a stretch factor. Decays of the magnetisation vs. time were obtained by the application of a magnetic field of 7 T to the sample at each temperature for 5 min, and then quick removal of the magnetic field.

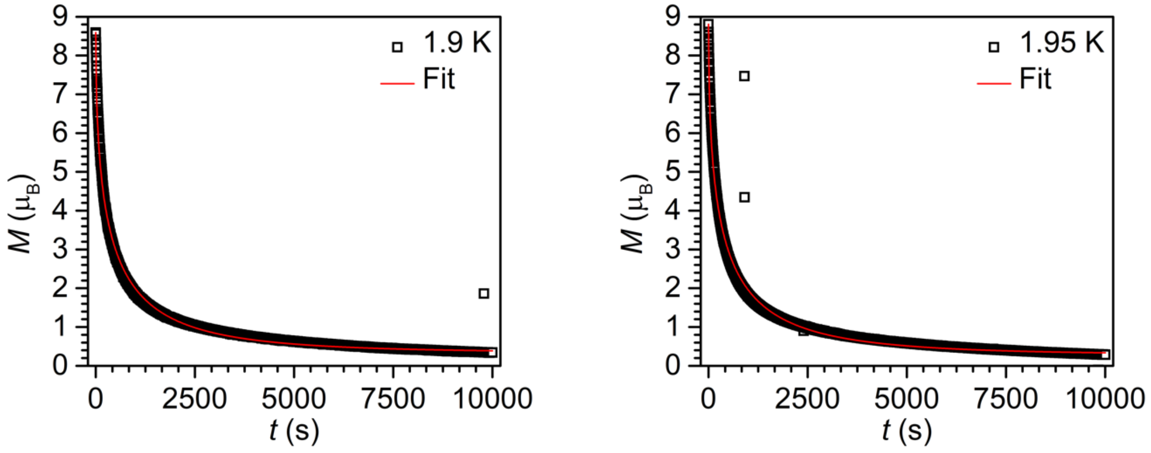

Figure S22. Plot of magnetisation vs. time used to derive relaxation times for $[(Cp^*{}_2Dy)_2(\mu\text{-}tan^{\bullet})][BArF_{20}]$, **2**, at 1.9 K (left) and 1.95 K (right). The data (black squares) were fit (red line) to a function of the form $M(t) = M_{eq} + (M_0 - M_{eq}) \exp\left(-\left(\frac{t}{\tau*}\right)^{\beta}\right)$ where β is a stretch factor. Decays of the magnetisation vs. time were obtained by the application of a magnetic field of 7 T to the sample at each temperature for 5 min, and then quick removal of the magnetic field.

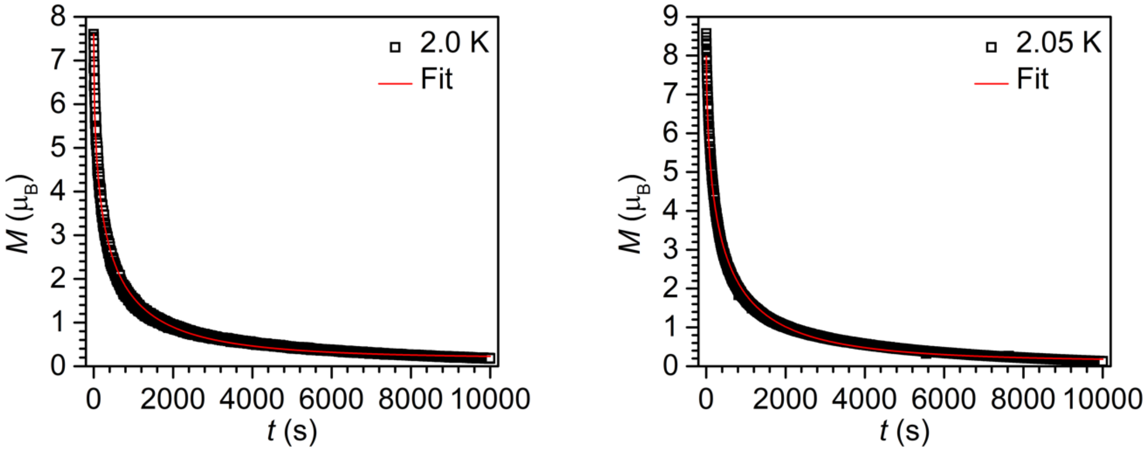

Figure S23. Plot of magnetisation vs. time used to derive relaxation times for [(Cp*$_2$Dy)$_2$(μ-tan$^•$)][BArF$_{20}$], **2**, at 2.0 K (left) and 2.05 K (right). The data (black squares) were fit (red line) to a function of the form $M(t) = M_{eq} + \left(M_0 - M_{eq}\right)\exp\left(-\left(\frac{t}{\tau*}\right)^{\beta}\right)$ where β is a stretch factor. Decays of the magnetisation vs. time were obtained by the application of a magnetic field of 7 T to the sample at each temperature for 5 min, and then quick removal of the magnetic field.

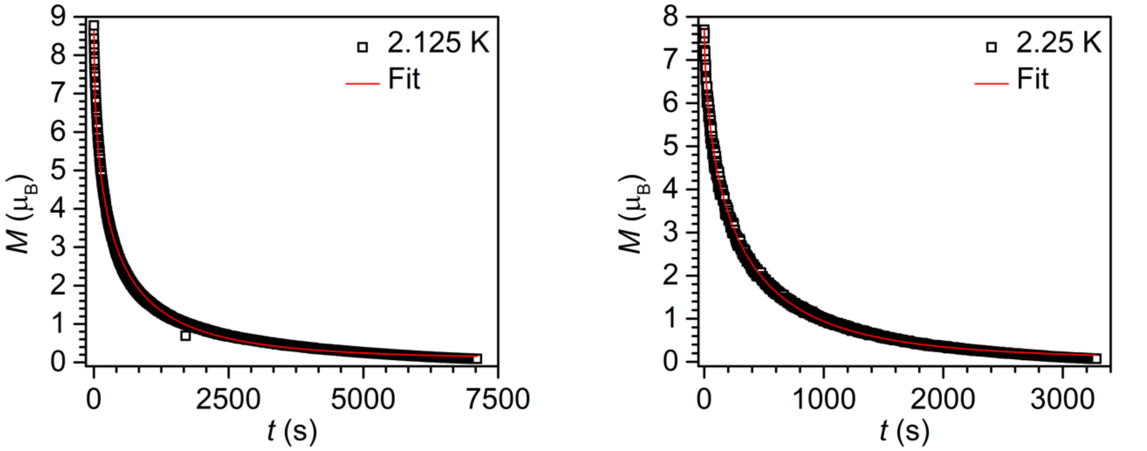

Figure S24. Plot of magnetisation vs. time used to derive relaxation times for [(Cp*$_2$Dy)$_2$(μ-tan$^•$)][BArF$_{20}$], **2**, at 2.125 K (left) and 2.25 K (right). The data (black squares) were fit (red line) to a function of the form $M(t) = M_{eq} + \left(M_0 - M_{eq}\right)\exp\left(-\left(\frac{t}{\tau*}\right)^{\beta}\right)$ where β is a stretch factor. Decays of the magnetisation vs. time were obtained by the application of a magnetic field of 7 T to the sample at each temperature for 5 min, and then quick removal of the magnetic field.

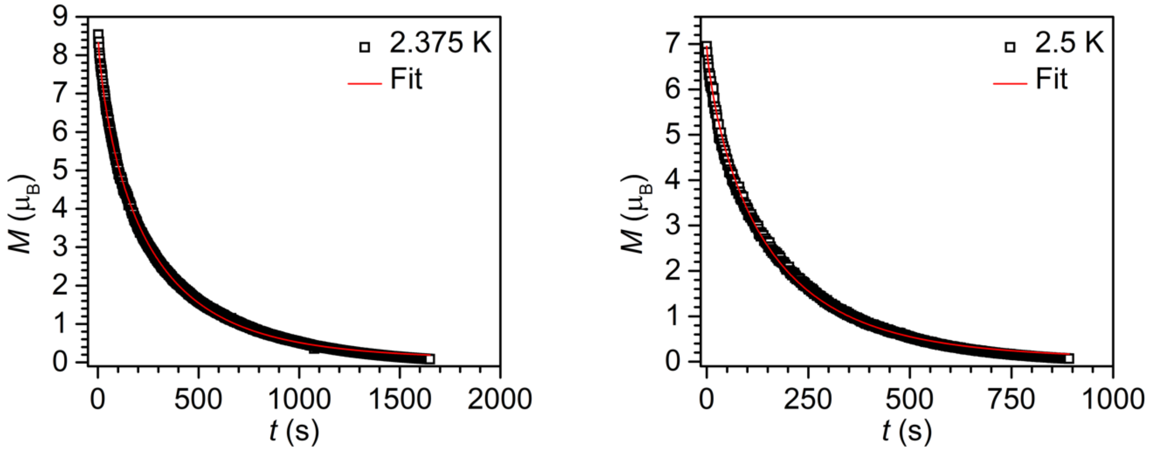

Figure S25. Plot of magnetisation vs. time used to derive relaxation times for [(Cp*$_2$Dy)$_2$(μ-tan$^•$)][BArF$_{20}$], **2**, at 2.375 K (left) and 2.5 K (right). The data (black squares) were fit (red line) to a function of the form $M(t) = M_{eq} + (M_0 - M_{eq})\exp\left(-\left(\frac{t}{\tau*}\right)^{\beta}\right)$ where β is a stretch factor. Decays of the magnetisation vs. time were obtained by the application of a magnetic field of 7 T to the sample at each temperature for 5 min, and then quick removal of the magnetic field.

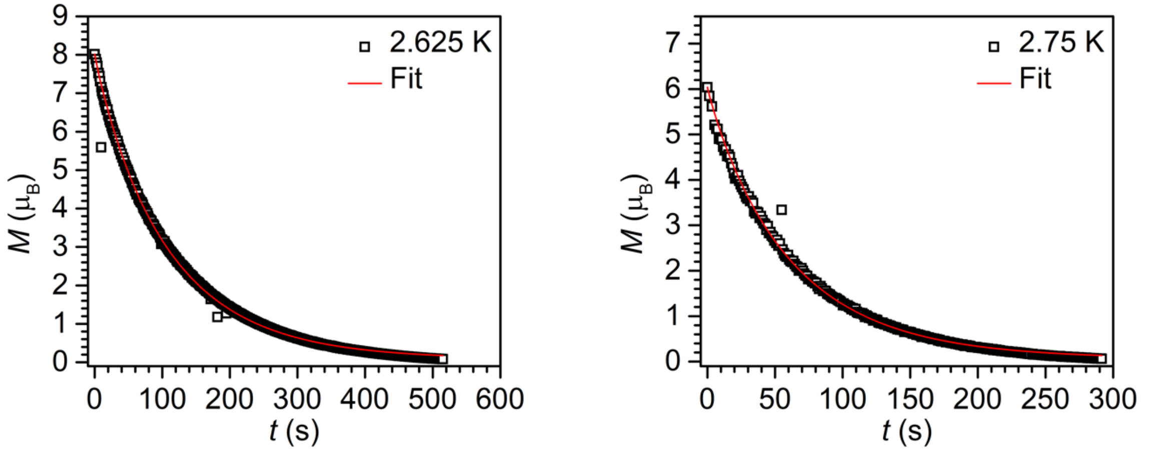

Figure S26. Plot of magnetisation vs. time used to derive relaxation times for [(Cp*$_2$Dy)$_2$(μ-tan$^•$)][BArF$_{20}$], **2**, at 2.625 K (left) and 2.75 K (right). The data (black squares) were fit (red line) to a function of the form $M(t) = M_{eq} + (M_0 - M_{eq})\exp\left(-\left(\frac{t}{\tau*}\right)^{\beta}\right)$ where β is a stretch factor. Decays of the magnetisation vs. time were obtained by the application of a magnetic field of 7 T to the sample at each temperature for 5 min, and then quick removal of the magnetic field.

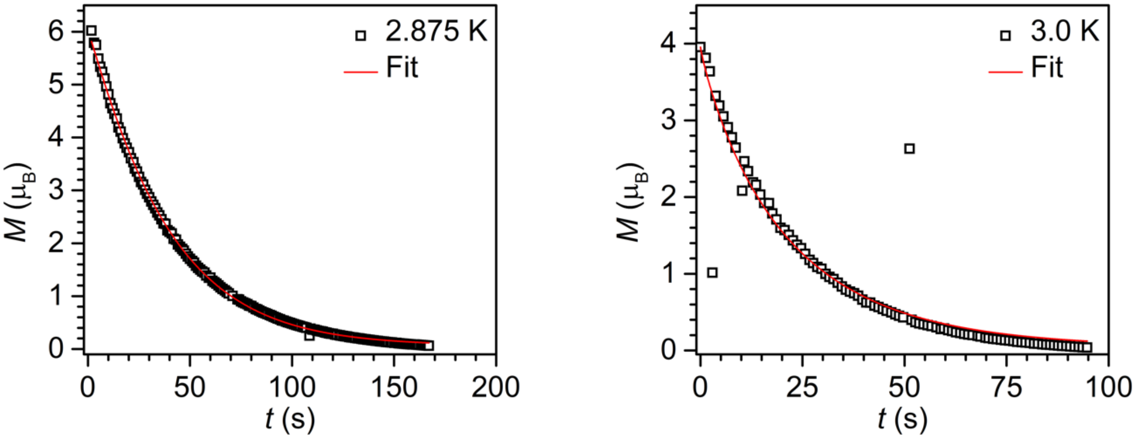

Figure S27. Plot of magnetisation vs. time used to derive relaxation times for [(Cp*$_2$Dy)$_2$(μ-tan$^•$)][BArF$_{20}$], **2**, at 2.875 K (left) and 3.0 K (right). The data (black squares) were fit (red line) to a function of the form $M(t) = M_{eq} + (M_0 - M_{eq}) \exp\left(-\left(\frac{t}{\tau*}\right)^\beta\right)$ where β is a stretch factor. Decays of the magnetisation vs. time were obtained by the application of a magnetic field of 7 T to the sample at each temperature for 5 min, and then quick removal of the magnetic field.

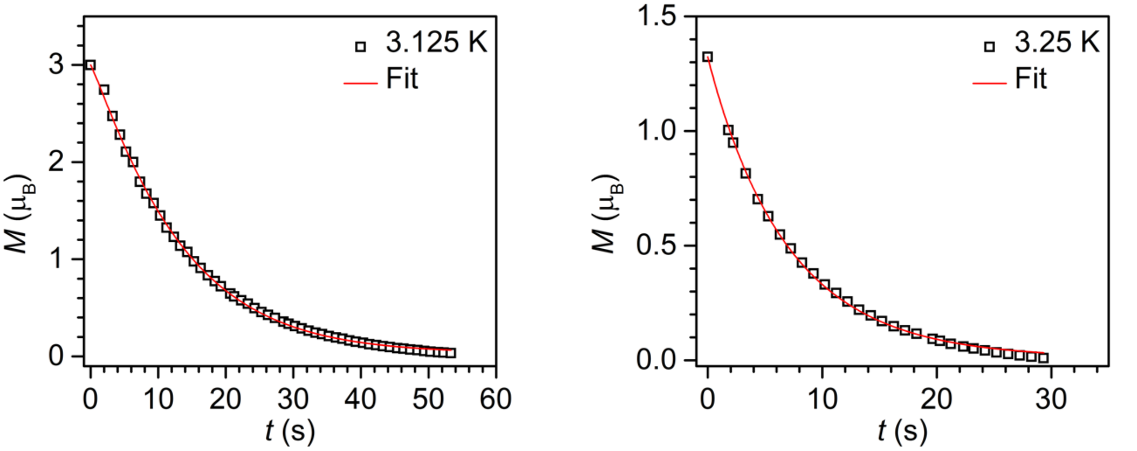

Figure S28. Plot of magnetisation vs. time used to derive relaxation times for [(Cp*$_2$Dy)$_2$(μ-tan$^•$)][BArF$_{20}$], **2**, at 3.125 K (left) and 3.25 K (right). The data (black squares) were fit (red line) to a function of the form $M(t) = M_{eq} + (M_0 - M_{eq}) \exp\left(-\left(\frac{t}{\tau*}\right)^\beta\right)$ where β is a stretch factor. Decays of the magnetisation vs. time were obtained by the application of a magnetic field of 7 T to the sample at each temperature for 5 min, and then quick removal of the magnetic field.

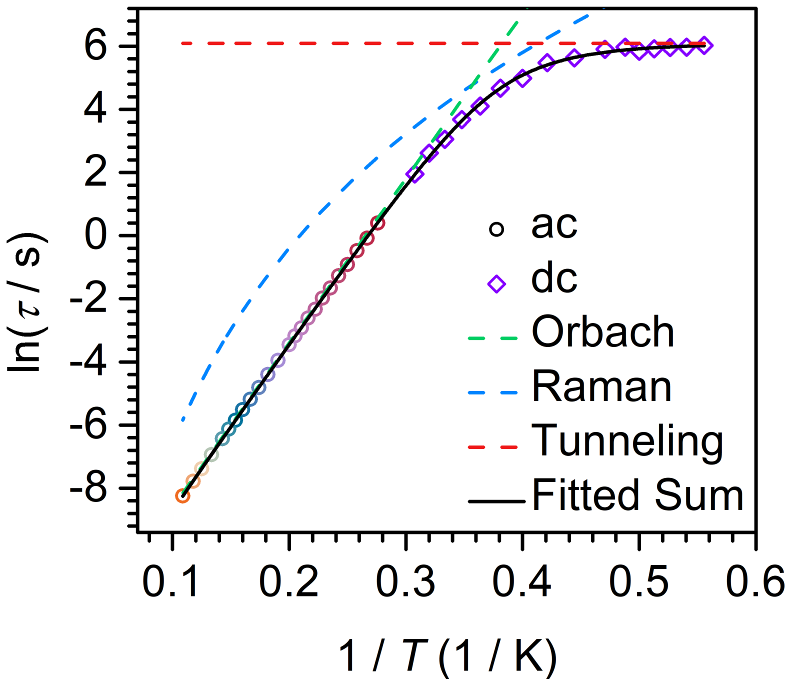

Figure S29. Individual contributions of the multiple magnetic relaxation pathways to the Arrhenius plot of $[(Cp^*{}_2Dy)_2(\mu\text{-tan}^{\bullet})][BArF_{20}]$, **2**, at 0 Oe dc field from 1.8 to 9.2 K. Individual parameters used to calculate the contributions are given in Table 3. Red to orange circles represent data extracted from ac magnetic susceptibility measurements, and purple diamonds represent data extracted from dc relaxation experiments. The black line represents a fit to an Orbach relaxation process, a Raman relaxation process, and a quantum tunnelling pathway. The Orbach parameters were freely refined.

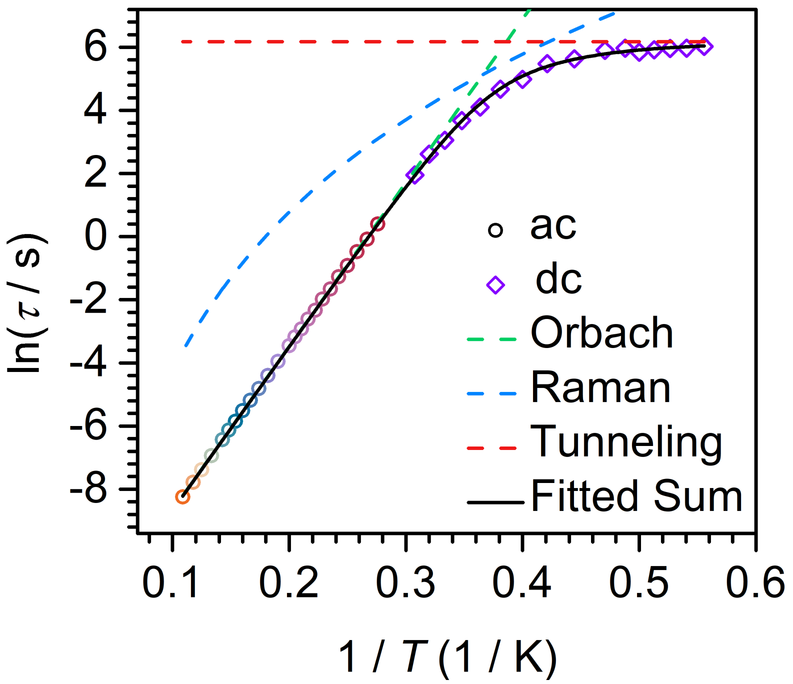

Figure S30. Individual contributions of the multiple magnetic relaxation pathways to the Arrhenius plot of $[(Cp^*{}_2Dy)_2(\mu\text{-tan}^{\bullet})][BArF_{20}]$, **2**, at 0 Oe dc field from 1.8 to 9.2 K. Individual parameters used to calculate the contributions are given in Table 3. Red to orange circles represent data extracted from ac magnetic susceptibility measurements, and purple diamonds represent data extracted from dc relaxation experiments. The black line represents a fit to an Orbach relaxation process, a Raman relaxation process, and a quantum tunnelling pathway. The Orbach parameters were fixed to the values obtained from fitting ac magnetic susceptibility data alone.

Table S2. Relaxation times of **1** and **2** at various temperatures. Data in blue and green were extracted from ac susceptibility measurements and dc relaxation experiments, respectively.

**1**				**2**			
T **(K)**	***τ*** **(s)**	***α***	***β***	***T*** **(K)**	***τ*** **(s)**	***α***	***β***
5.500	4.182E-01	0.060	-	1.800	4.152E+02	-	0.509
6.000	2.451E-01	0.046	-	1.850	3.927E+02	-	0.505
6.500	1.502E-01	0.037	-	1.900	3.882E+02	-	0.503
7.000	9.559E-02	0.030	-	1.950	3.729E+02	-	0.494
8.000	4.326E-02	0.022	-	2.000	3.438E+02	-	0.482
9.000	2.217E-02	0.019	-	2.050	3.930E+02	-	0.498
10.000	1.250E-02	0.016	-	2.125	3.669E+02	-	0.530
11.000	7.590E-03	0.016	-	2.250	2.802E+02	-	0.609
12.000	4.900E-03	0.015	-	2.375	2.395E+02	-	0.760
14.000	2.320E-03	0.018	-	2.500	1.465E+02	-	0.793
16.000	1.220E-03	0.021	-	2.625	1.064E+02	-	0.943
17.000	8.954E-04	0.025	-	2.750	6.054E+01	-	0.934
18.000	6.562E-04	0.031	-	2.875	3.962E+01	-	1.073
19.000	4.700E-04	0.042	-	3.000	2.116E+01	-	0.896
20.000	3.351E-04	0.047	-	3.125	1.366E+01	-	1.107
21.000	2.407E-04	0.037	-	3.250	7.046E+00	-	0.977
				3.625	1.493E+00	0.035	-
				3.750	9.253E-01	0.035	-
				3.875	6.199E-01	0.036	-
				4.000	4.002E-01	0.032	-
				4.125	2.800E-01	0.034	-
				4.250	1.899E-01	0.031	-
				4.375	1.382E-01	0.031	-
				4.500	9.731E-02	0.029	-
				4.625	7.366E-02	0.029	-
				4.750	5.377E-02	0.026	-
				4.875	4.169E-02	0.026	-
				5.000	3.152E-02	0.024	-
				5.250	1.924E-02	0.023	-
				5.500	1.226E-02	0.022	-
				5.750	8.127E-03	0.023	-
				6.000	5.601E-03	0.021	-
				6.250	4.045E-03	0.022	-
				6.500	2.876E-03	0.022	-
				6.750	2.171E-03	0.023	-
				7.000	1.609E-03	0.024	-
				7.500	9.718E-04	0.023	-
				8.000	6.188E-04	0.023	-
				8.500	4.180E-04	0.022	-
				9.200	2.613E-04	0.011	-

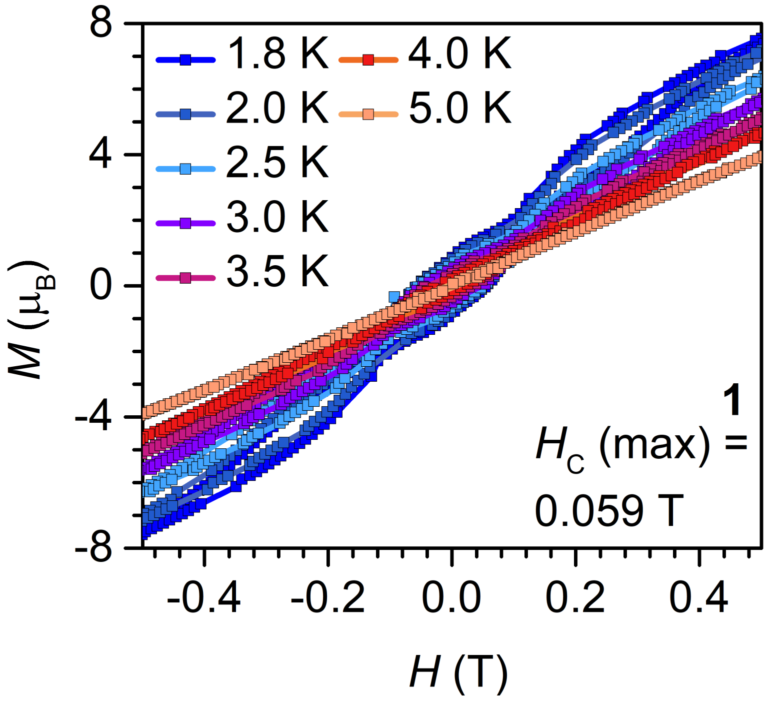

Figure S31. Plot of magnetisation (*M*) vs dc magnetic field (*H*) at an average sweep rate of 0.01 T/s for [(Cp*$_2$Dy)$_2$(μ-tan), **1**, from 1.8 to 5.0 K between ±0.45 T.

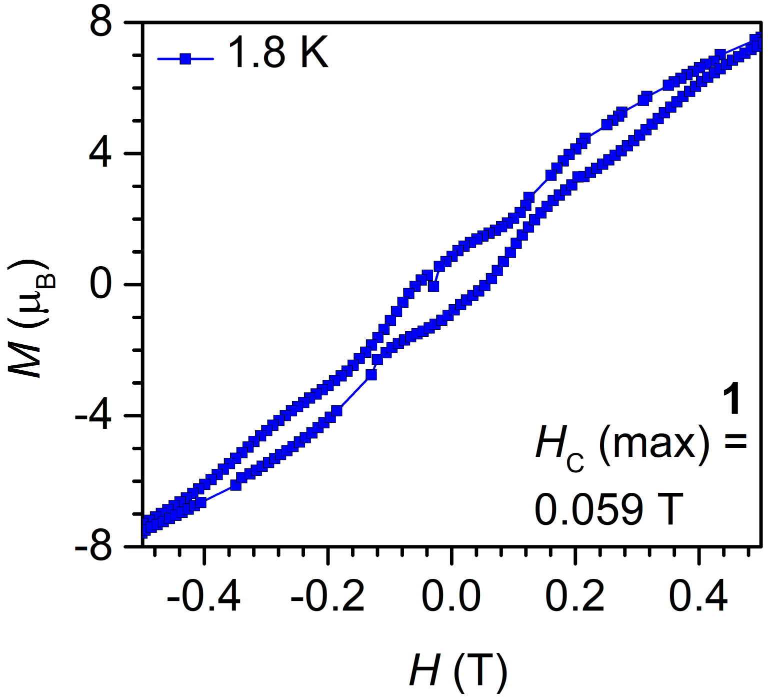

Figure S32. Plot of magnetisation (*M*) vs dc magnetic field (*H*) at an average sweep rate of 0.01 T/s for [(Cp*$_2$Dy)$_2$(μ-tan$^•$)], **1**, at 1.8 K between ±0.45 T.

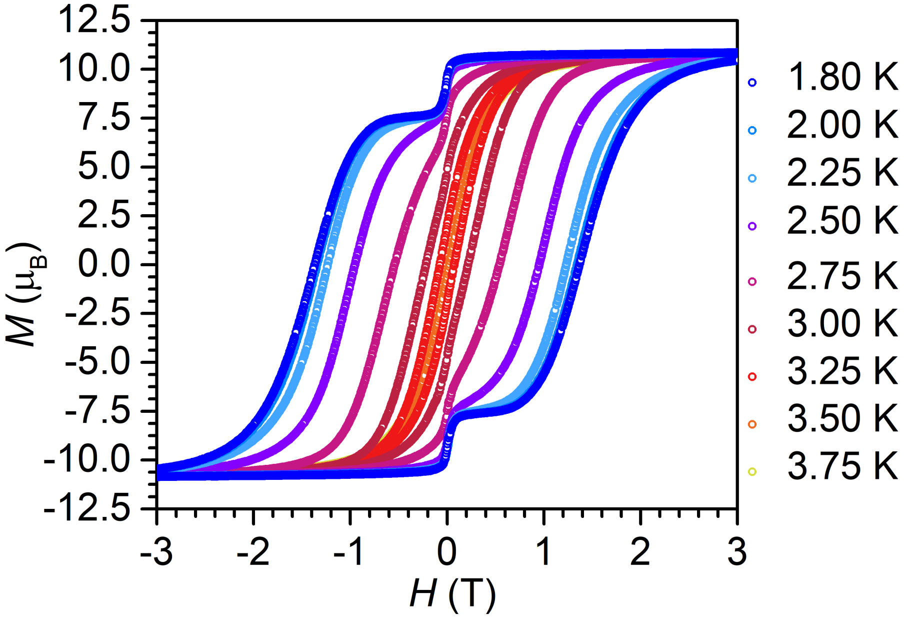

Figure S33. Plot of magnetisation (*M*) vs dc magnetic field (*H*) at an average sweep rate of 0.01 T/s for [$(Cp^*{}_2Dy)_2$(μ-tan$^•$)][$BArF_{20}$], **2**, from 1.8 to 3.75 K between ±3 T.

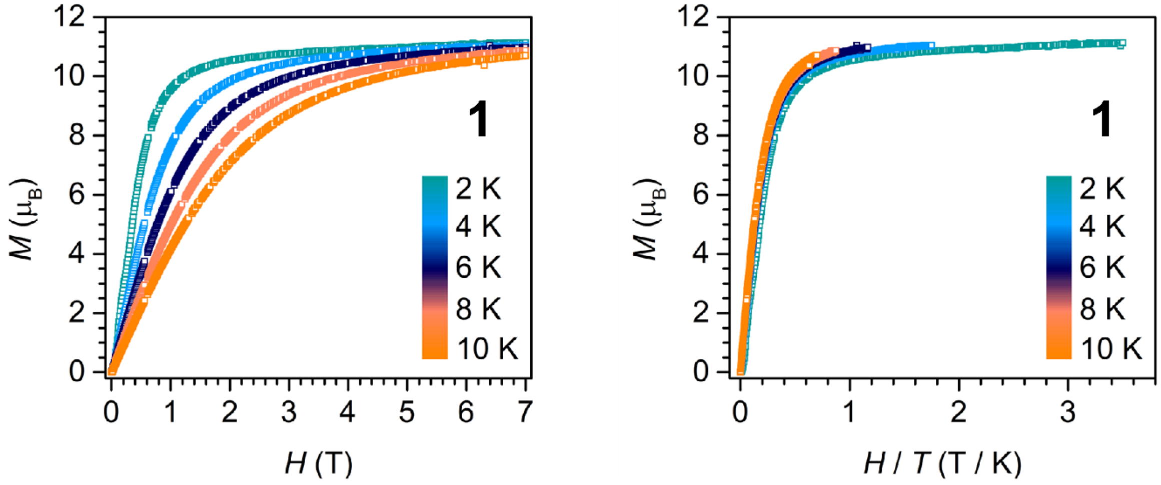

Figure S34. Variable-temperature field-dependent magnetisation curves (left) and reduced magnetisation data (right) for **1**, collected from 0 to 7 T at 2, 4, 6, 8, and 10 K.

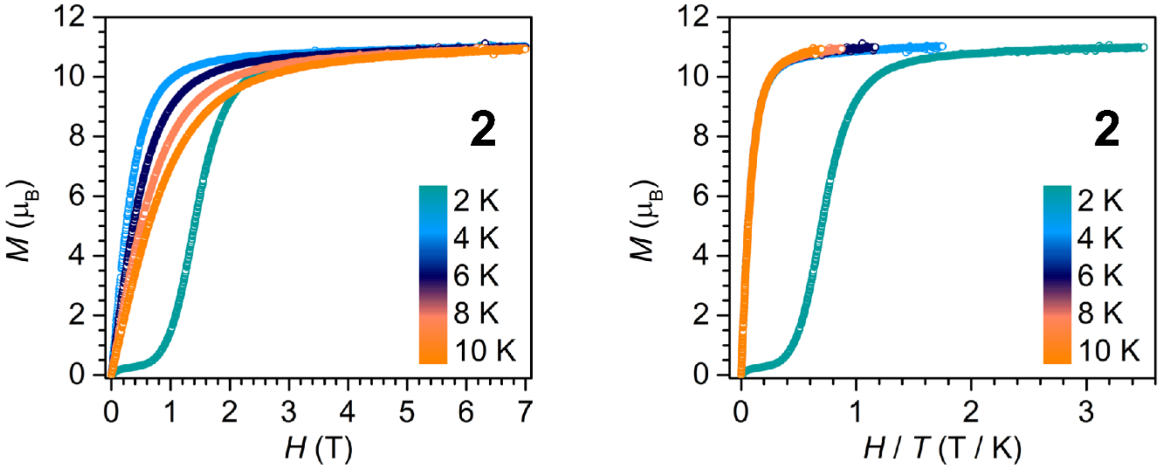

Figure S35. Variable-temperature field-dependent magnetisation curves (left) and reduced magnetisation data (right) for **2**, collected from 0 to 7 T at 2, 4, 6, 8, and 10 K.

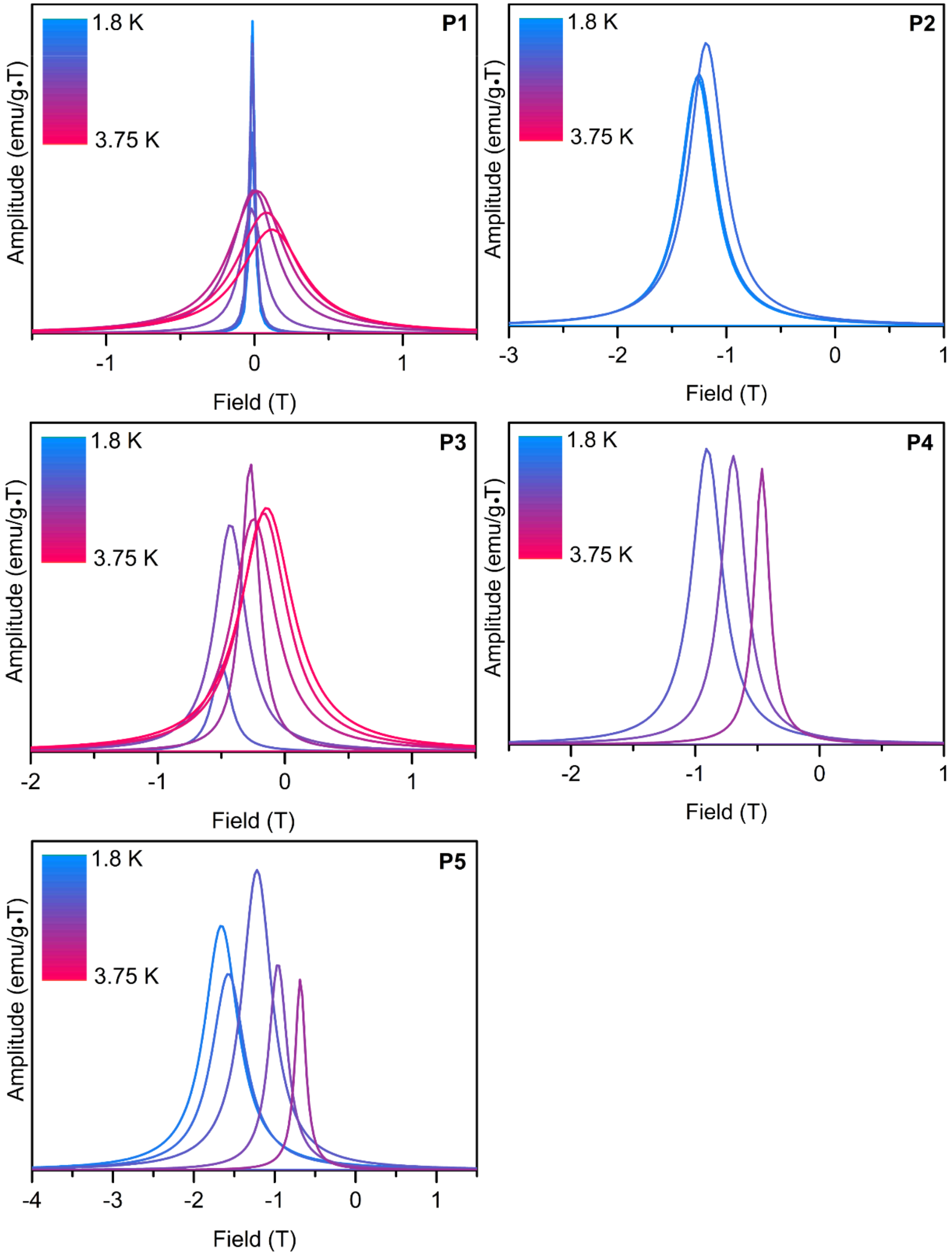

Figure S36. Temperature dependence of the four demagnetisation processes identified in the first derivative of the magnetic hysteresis of $[(Cp^*{}_2Dy)_2(\mu\text{-tan}^{\bullet})][BArF_{20}]$, **2**, through Cauchy probability distribution function analysis. The five processes are labelled as P1, P2, P3, P4, and P5 and their variation in peak position on the field and peak amplitude are plotted from 1.8 K to 3.75 K. Only P1 is present in the full temperature range, while P2 contribution disappears after 2.25 K. P3 is observed above 2.5 K, and P4 appears only at temperatures between 2.5 K and 3 K. The P5 contribution is not observed beyond 3.25 K.

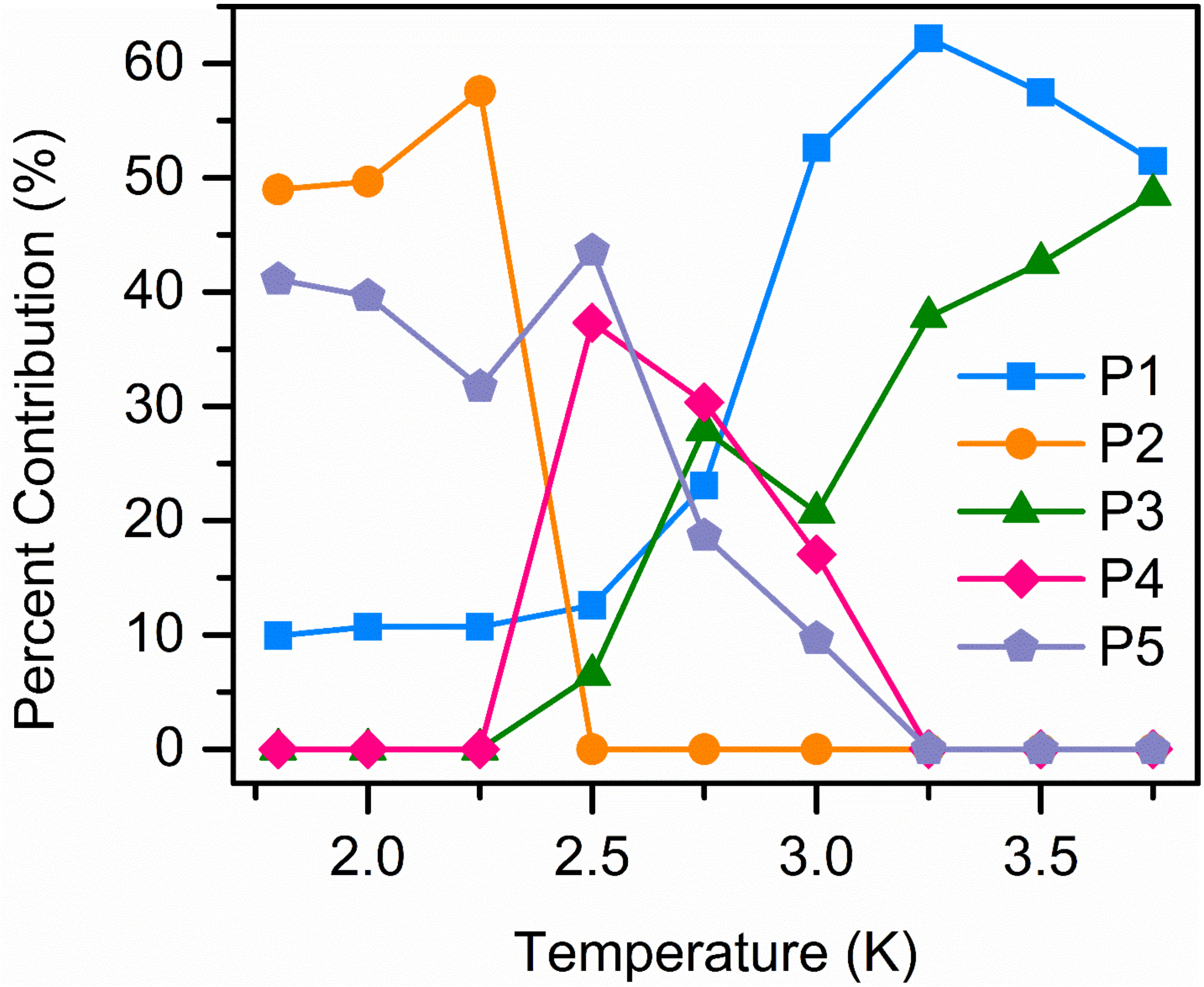

Figure S37. Temperature dependence of the percent contributions of the five demagnetisation processes identified in the first derivative of the magnetic hysteresis of $[(Cp^*{}_2Dy)_2(\mu\text{-tan}^{\bullet})][BArF_{20}]$, **2**, through Cauchy probability distribution function analysis.

Table S3. Cauchy probability distribution function analysis data of the five distinct demagnetisation processes determined in the first derivative of magnetic hysteresis of $[(Cp^*{}_2Dy)_2(\mu\text{-tan}^\bullet)][BArF_{20}]$, **2**, from 1.8 K to 3.75 K. (γ = half-width at half-maximum, PC = percent contribution, F = field position). Fit parameters are for the arctan function fitting of the experimental magnetic hysteresis data.

Temp. (K)	Demagnetisation Processes					Fit Parameters	
	**P1**	**P2**	**P3**	**P4**	**P5**	R^2	**Reduced χ^2**
**1.8**	PC = 9.94% F = –0.014 T γ = 0.016 T	PC = 48.97% F = –1.26 T γ = 0.19 T	PC = 0% F = NA γ = NA	PC = 0% F = NA γ = NA	PC = 41.09% F = –1.69 T γ = 0.26 T	0.999	0.0853
**2.0**	PC = 10.70% F = –0.013 T γ = 0.017 T	PC = 49.66% F = –1.25 T γ = 0.19 T	PC = 0% F = NA γ = NA	PC = 0% F = NA γ = NA	PC = 39.64% F = –1.66 T γ = 0.26 T	0.998	0.2584
**2.25**	PC = 10.71% F = –0.018 T γ = 0.012 T	PC = 57.58% F = –1.18 T γ = 0.19 T	PC = 0% F = NA γ = NA	PC = 0% F = NA γ = NA	PC = 31.70% F = –1.57 T γ = 0.25 T	0.999	0.0715
**2.5**	PC = 12.59% F = –0.018 T γ = 0.020 T	PC = 0% F = NA γ = NA	PC = 6.51% F = –0.50 T γ = 0.089 T	PC = 37.32% F = –0.91 T γ = 0.15 T	PC = 43.59% F = –1.22 T γ = 0.23 T	0.999	0.0563
**2.75**	PC = 23.07% F = –0.019 T γ = 0.089 T	PC = 0% F = NA γ = NA	PC = 27.94% F = –0.43 T γ = 0.15 T	PC = 30.35% F = –0.69 T γ = 0.12 T	PC = 18.65% F = –0.96 T γ = 0.14 T	0.999	0.0147
**3.0**	PC = 52.6% F = –0.004 T γ = 0.18 T	PC = 0% F = NA γ = NA	PC = 20.7% F = –0.27 T γ = 0.085 T	PC = 17.03% F = –0.46 T γ = 0.072 T	PC = 9.67% F = –0.68 T γ = 0.079 T	0.999	0.0231
**3.25**	PC = 62.19% F = 0.018 T γ = 0.22 T	PC = 0% F = NA γ = NA	PC = 37.81% F = –0.25 T γ = 0.20 T	PC = 0% F = NA γ = NA	PC = 0% F = NA γ = NA	0.999	0.0106

**3.5**	PC = 57.45%<br>F = 0.079 T<br>γ = 0.24 T	PC = 0%<br>F = NA<br>γ = NA	PC = 42.55%<br>F = –0.17 T<br>γ = 0.23 T	PC = 0%<br>F = NA<br>γ = NA	PC = 0%<br>F = NA<br>γ = NA	0.999	0.0107
**3.75**	PC = 51.47%<br>F = 0.117 T<br>γ = 0.26 T	PC = 0%<br>F = NA<br>γ = NA	PC = 48.53%<br>F = –0.14 T<br>γ = 0.25 T	PC = 0%<br>F = NA<br>γ = NA	PC = 0%<br>F = NA<br>γ = NA	0.999	0.0119

6 DFT Calculations

Table S4. Results of the broken-symmetry DFT calculations performed on the crystal coordinates of $[(Cp^*_2Dy)_2(\mu\text{-tan}^\bullet)]^+$ of $[(Cp^*_2Dy)_2(\mu\text{-tan}^\bullet)][BArF_{20}]$, **2**, substituting Dy with Gd. SARC2-DKH-QZVP basis was used for Gd atoms, ZORA-def2-TZVPP was employed for all tan atoms, and DKH-def2-TZVP basis was used for all atoms. The calculation employed D3BJ dispersion correction and SARC/J auxiliary basis set. Spin on all tan atoms were flipped with the spinflip function. The calculation was conducted using TPSS0 functional. The exchange coupling constant (*J*) was calculated for the different molecular units in the unit cell and the two disordered parts. M1 is the edge-centred molecular unit in the unit cell and M2A and M2B are the two disordered parts of the face-centred molecular unit.

Molecular Unit/Disorder	J (cm^{-1})
M1	–17.71
M2A	–16.56
M2B	–16.67

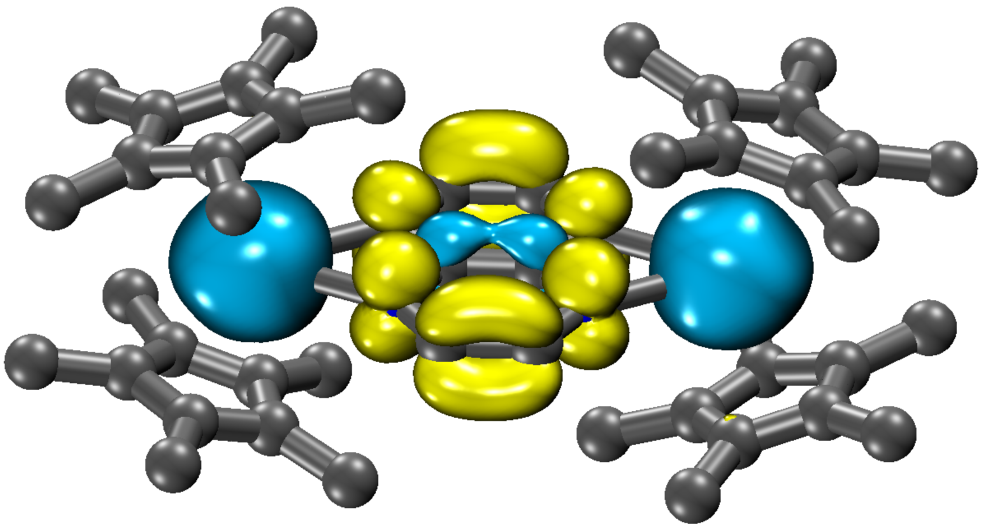

Figure S38. Spin density plot generated for the model system of $[(Cp^*_2Gd)_2(\mu\text{-tan}^\bullet)]^+$ in $\mathbf{2^{Gd}}$. This system is obtained by substituting the Dy atoms of $[(Cp^*_2Dy)_2(\mu\text{-tan}^\bullet)][BArF_{20}]$, **2**, with Gd. Grey, blue, and orange spheres represent, C, N, and Gd atoms. N and Gd atoms are covered by yellow and light-blue spin density surfaces. Isovalue for all surfaces is set to 0.003. H atoms are omitted for clarity. Molecular unit M2B is shown here.

Table S5. Majority contributions of the TD-DFT–calculated transition states for [(Cp*$_2$Dy)$_2$(μ-tan)], **1**, at the def2-TZVP level using the uTPSS0 functional with D3BJ dispersion correction and DCM implicit solvent model. For Dy^{III}, pseudopotential ECP55MWB and the associated ECP55MWB-II basis set were used. The calculated excitation energies were empirically shifted by 0.34 eV. Isovalue for all depictions is 0.03. Oscillator strength cutoff used is 0.05 and contributions higher than 15% are shown. (HOMO = 194, LUMO = 195)

λ (nm)	ν (cm^{-1})	Oscillator Strength	Occupied	Virtual	Weight (%)
499.6	20016	0.33476	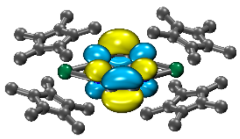 194α	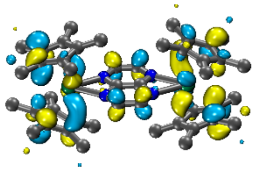 207α	29.4
			194β	207β	29.4
550.8	18155	0.26607	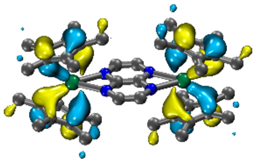 191α	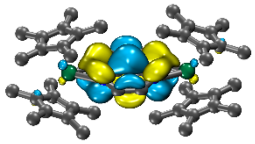 195α	49.4
			191β	195β	49.4
692.8	14434	0.14988	194α	195α	47.2
			109β	116β	47.2
482.3	20734	0.10908	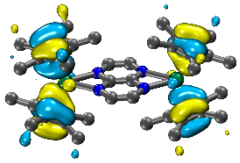 193α	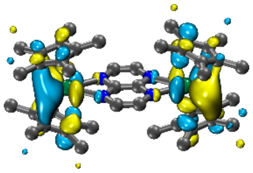 201α	19.6
			193β	201β	19.6
			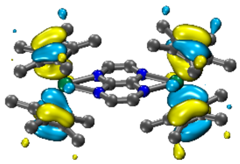 192α	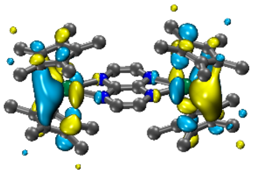 202α	15.8
			192β	202β	15.8

480.0	20833	0.10908	194α	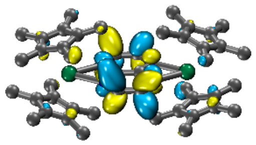 215α	39.9
			194β	215β	39.9
544.3	18372	0.10347	193α	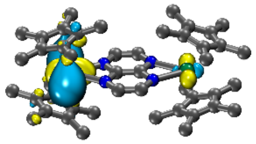 197α	20.2
			193β	197β	20.2
541.1	18481	0.06575	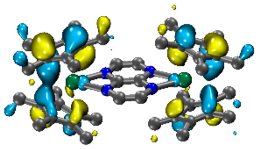 189α	195α	46.8
			189β	195β	46.8
499.4	20024	0.06053	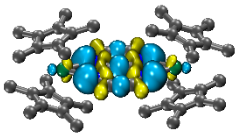 185α	195α	32.9
			185β	195β	32.9

Table S6. Majority contributions of the TD-DFT–calculated transition states for $[(Cp^*_2Dy)_2(\mu\text{-}tan^{\bullet})][BArF_{20}]$, **2**, at the def2-TZVP level using the uTPSS0 functional with D3BJ dispersion correction and DCM implicit solvent model. For Dy^{III}, pseudopotential ECP55MWB and the associated ECP55MWB-II basis set were used. The calculated excitation energies were empirically shifted by 0.34 eV. Isovalue for all depictions is 0.03. Oscillator strength cutoff used is 0.05 and contributions higher than 15% are shown. (HOMO = 193, SOMO = 194, LUMO = 195)

λ (nm)	ν (cm^{-1})	Oscillator Strength	Occupied	Virtual	Weight (%)
505.8	19771	0.14528	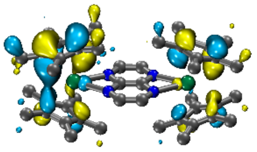 191β	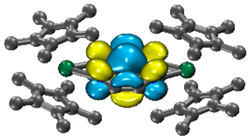 195β	34.7
			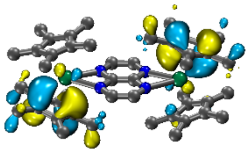 189β	195β	24.3
498.4	20064	0.14304	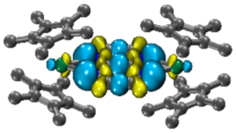 185β	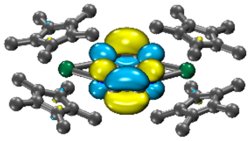 194β	68.5
405.7	24649	0.13659	194α	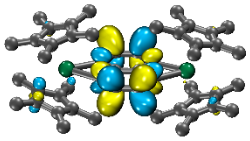 203α	28.2
			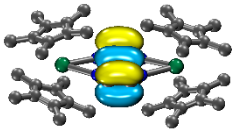 184α	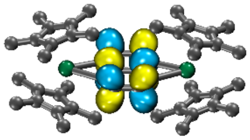 196α	16.6
487.1	20529	0.12214	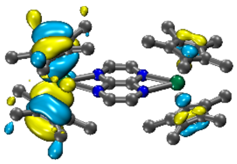 193α	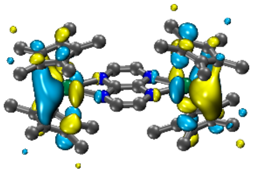 197α	25.5
			193β	197β	24.3

415.7	24056	0.09435	191α	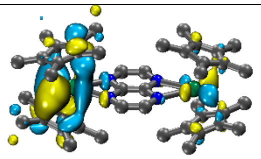 202α	19.8
			191β	202β	19.6
565.1	17696	0.07393	191α	195α	44.4
			189β	197β	20.2
693.1	14428	0.06575	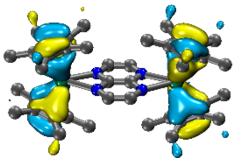 187β	194β	99.4
417.7	23941	0.05577	No individual contributions above 15%		
462.8	21608	0.05321	194α	203α	34.9
			185β	196β	30.9

7 Python script used for fitting the first derivative of the magnetic hysteresis loops to arctangents

```
import pandas as pd
import numpy as np
from scipy.optimize import curve_fit
import matplotlib.pyplot as plt
import os

# Step 1: Define the file path and load the CSV file
file_path = "xxxxx/xxxx.csv"
try:
    data = pd.read_csv(file_path)
except FileNotFoundError:
    print(f"Error: The file {file_path} was not found.")
    exit()

# Step 2: Extract the magnetic field and moment per gram columns
H = data['Field_True'].values  # Magnetic field in Tesla
M_g = data['Moment_emu_g'].values  # Magnetic moment per gram in emu/g

# Step 3: Check for invalid data
if not (np.all(np.isfinite(H)) and np.all(np.isfinite(M_g))):
    print("Error: Data contains NaN or infinite values.")
    exit()

# Step 4: Split into descending and ascending branches
k = H.argmin()  # Index of minimum field
H_desc = H[:k+1]  # Descending branch
M_g_desc = M_g[:k+1]
H_asc = H[k:]     # Ascending branch
M_g_asc = M_g[k:]

# Step 5: Define the scaled arctangent model with four components and baseline
def arctan_model(H, Ms1, a1, Hc1, Ms2, a2, Hc2, Ms3, a3, Hc3, Ms4, a4, Hc4, baseline):
    """
    Scaled Model: M(H) = Ms1 * (2/pi) * arctan(a1 * (H - Hc1)) + Ms2 * (2/pi) * arctan(a2 * (H - Hc2))
+ Ms3 * (2/pi) * arctan(a3 * (H - Hc3)) + Ms4 * (2/pi) * arctan(a4 * (H - Hc4)) + baseline
    Parameters:
        Ms1, Ms2, Ms3, Ms4: Saturation magnetizations per gram (emu/g)
        a1, a2, a3, a4: Steepness factors
        Hc1, Hc2, Hc3, Hc4: Coercive fields (Tesla)
        baseline: Constant offset (emu/g)
    """
    return (Ms1 * (2 / np.pi) * np.arctan(a1 * (H - Hc1)) +
            Ms2 * (2 / np.pi) * np.arctan(a2 * (H - Hc2)) +
            Ms3 * (2 / np.pi) * np.arctan(a3 * (H - Hc3)) +
            Ms4 * (2 / np.pi) * np.arctan(a4 * (H - Hc4)) +
```

```
            baseline)

# Step 6: Set initial guesses and bounds
p0 = [15, 15, 0.05, 15, 15, -0.5, 4, 50, 0.03, 2, 60, 0.01, 0.0]  # [Ms1, a1, Hc1, Ms2, a2, Hc2, Ms3,
a3, Hc3, Ms4, a4, Hc4, baseline]
bounds = (
    [0, 1, -2.5, 0, 1, -2.5, 0, 1, -0.7, 0, 1, -0.5, -10],  # Lower bounds
    [50, 50, 2.5, 50, 50, 2.5, 50, 50, 0.7, 50, 100, 0.5, 10]  # Upper bounds
)

# Step 7: Fit the descending branch
try:
    popt_desc, _ = curve_fit(
        arctan_model, H_desc, M_g_desc, p0=p0, bounds=bounds, maxfev=40000
    )
    print("Descending branch fit parameters [Ms1, a1, Hc1, Ms2, a2, Hc2, Ms3, a3, Hc3, Ms4, a4,
Hc4, baseline]:", popt_desc)
except RuntimeError as e:
    print("Error fitting descending branch:", e)

# Step 8: Fit the ascending branch
p0_asc = [15, 15, -0.05, 15, 15, 0.5, 4, 50, -0.03, 2, 60, -0.01, 0.0]
try:
    popt_asc, _ = curve_fit(
        arctan_model, H_asc, M_g_asc, p0=p0_asc, bounds=bounds, maxfev=40000
    )
    print("Ascending branch fit parameters [Ms1, a1, Hc1, Ms2, a2, Hc2, Ms3, a3, Hc3, Ms4, a4,
Hc4, baseline]:", popt_asc)
except RuntimeError as e:
    print("Error fitting ascending branch:", e)

# Step 9: Generate fitted curves
H_fit_desc = np.linspace(min(H_desc), max(H_desc), 500)
M_g_fit_desc = arctan_model(H_fit_desc, *popt_desc)
H_fit_asc = np.linspace(min(H_asc), max(H_asc), 500)
M_g_fit_asc = arctan_model(H_fit_asc, *popt_asc)

# Step 10: Compute goodness-of-fit metrics
def goodness_of_fit(y_true, y_pred):
    mask = np.isfinite(y_true) & np.isfinite(y_pred)
    y_true = y_true[mask]
    y_pred = y_pred[mask]

    ss_tot = np.sum((y_true - np.mean(y_true))**2)
    ss_res = np.sum((y_true - y_pred)**2)
    r_squared = 1 - ss_res / ss_tot if ss_tot != 0 else np.nan

    rmse = np.sqrt(np.mean((y_true - y_pred)**2))

    chi_squared = np.sum((y_true - y_pred)**2)
    dof = len(y_true) - len(popt_desc)
```

```
    reduced_chi_squared = chi_squared / dof if dof > 0 else np.nan

    return r_squared, rmse, chi_squared, reduced_chi_squared

# Interpolate the fit to match raw data points
interp_desc = np.interp(H_desc, H_fit_desc, M_g_fit_desc)
interp_asc = np.interp(H_asc, H_fit_asc, M_g_fit_asc)

# Compute metrics
r2_desc, rmse_desc, chi2_desc, reduced_chi2_desc = goodness_of_fit(M_g_desc, interp_desc)
r2_asc, rmse_asc, chi2_asc, reduced_chi2_asc = goodness_of_fit(M_g_asc, interp_asc)

# Step 11: Export fit lines to CSV
output_dir = os.path.dirname(file_path)
output_file = os.path.join(output_dir, "HysFit_SArcTan_FitLines.csv")
fit_data = {
    'Field_T': np.concatenate([H_fit_desc, H_fit_asc]),
    'Moment_emu_g_Descending_Fit': np.concatenate([M_g_fit_desc, np.full_like(M_g_fit_asc,
np.nan)]),
    'Moment_emu_g_Ascending_Fit': np.concatenate([np.full_like(M_g_fit_desc, np.nan),
M_g_fit_asc])
}
fit_df = pd.DataFrame(fit_data)
fit_df.to_csv(output_file, index=False)
print(f"Fit lines exported to {output_file}")

# Step 12: Calculate the first derivatives of the fit lines
def arctan_derivative(H, Ms1, a1, Hc1, Ms2, a2, Hc2, Ms3, a3, Hc3, Ms4, a4, Hc4, baseline):
    """
    First derivative of the scaled arctangent model with respect to H.
    dM/dH = (2/pi) * [ Ms1 * a1 / (1 + (a1*(H-Hc1))^2) + Ms2 * a2 / (1 + (a2*(H-Hc2))^2) + Ms3 * a3
/ (1 + (a3*(H-Hc3))^2) + Ms4 * a4 / (1 + (a4*(H-Hc4))^2) ]
    (Baseline does not contribute to the derivative)
    """
    term1 = Ms1 * (2 / np.pi) * a1 / (1 + (a1 * (H - Hc1))**2)
    term2 = Ms2 * (2 / np.pi) * a2 / (1 + (a2 * (H - Hc2))**2)
    term3 = Ms3 * (2 / np.pi) * a3 / (1 + (a3 * (H - Hc3))**2)
    term4 = Ms4 * (2 / np.pi) * a4 / (1 + (a4 * (H - Hc4))**2)
    return term1 + term2 + term3 + term4

# Compute derivatives
dM_dH_desc = arctan_derivative(H_fit_desc, *popt_desc)
dM_dH_asc = arctan_derivative(H_fit_asc, *popt_asc)

# Step 13: Export derivatives to CSV
output_deriv_file = os.path.join(output_dir, "HysFit_SArcTan_FitLinesDeriv.csv")
deriv_data = {
    'Field_T': np.concatenate([H_fit_desc, H_fit_asc]),
    'dM_dH_Descending_Fit': np.concatenate([dM_dH_desc, np.full_like(dM_dH_asc, np.nan)]),
    'dM_dH_Ascending_Fit': np.concatenate([np.full_like(dM_dH_desc, np.nan), dM_dH_asc])
}
```

```
deriv_df = pd.DataFrame(deriv_data)
deriv_df.to_csv(output_deriv_file, index=False)
print(f"Derivatives exported to {output_deriv_file}")

# Step 14: Export parameters with goodness-of-fit metrics
output_params_desc_file = os.path.join(output_dir,
"HysFit_SArcTan_Parameters_Descending_X.XK.txt")
with open(output_params_desc_file, 'w') as f:
    f.write("Scaled Arctangent Fit Parameters for Descending Branch (X.X K):\n")
    f.write("================================================\n\n")
    f.write(f"  Ms1 (Saturation Mag 1) = {popt_desc[0]:.4f} emu/g\n")
    f.write(f"  a1 (Steepness 1)       = {popt_desc[1]:.4f} T^-1\n")
    f.write(f"  Hc1 (Coercive Field 1) = {popt_desc[2]:.4f} T\n")
    f.write(f"  Ms2 (Saturation Mag 2) = {popt_desc[3]:.4f} emu/g\n")
    f.write(f"  a2 (Steepness 2)       = {popt_desc[4]:.4f} T^-1\n")
    f.write(f"  Hc2 (Coercive Field 2) = {popt_desc[5]:.4f} T\n")
    f.write(f"  Ms3 (Saturation Mag 3) = {popt_desc[6]:.4f} emu/g\n")
    f.write(f"  a3 (Steepness 3)       = {popt_desc[7]:.4f} T^-1\n")
    f.write(f"  Hc3 (Coercive Field 3) = {popt_desc[8]:.4f} T\n")
    f.write(f"  Ms4 (Saturation Mag 4) = {popt_desc[9]:.4f} emu/g\n")
    f.write(f"  a4 (Steepness 4)       = {popt_desc[10]:.4f} T^-1\n")
    f.write(f"  Hc4 (Coercive Field 4) = {popt_desc[11]:.4f} T\n")
    f.write(f"  Baseline               = {popt_desc[12]:.4f} emu/g\n")
    f.write("--------------------------------\n\n")
    f.write("Goodness-of-Fit Metrics (vs. Raw Data):\n")
    f.write("================================================\n")
    f.write(f"  R-squared              = {r2_desc:.4f}\n")
    f.write(f"  RMSE                   = {rmse_desc:.4f} emu/g\n")
    f.write(f"  Chi-squared            = {chi2_desc:.4f}\n")
    f.write(f"  Reduced Chi-squared  = {reduced_chi2_desc:.4f}\n")
print(f"Descending parameters exported to {output_params_desc_file}")

output_params_asc_file = os.path.join(output_dir,
"HysFit_SArcTan_Parameters_Ascending_X.XK.txt")
with open(output_params_asc_file, 'w') as f:
    f.write("Scaled Arctangent Fit Parameters for Ascending Branch (X.X K):\n")
    f.write("================================================\n\n")
    f.write(f"  Ms1 (Saturation Mag 1) = {popt_asc[0]:.4f} emu/g\n")
    f.write(f"  a1 (Steepness 1)       = {popt_asc[1]:.4f} T^-1\n")
    f.write(f"  Hc1 (Coercive Field 1) = {popt_asc[2]:.4f} T\n")
    f.write(f"  Ms2 (Saturation Mag 2) = {popt_asc[3]:.4f} emu/g\n")
    f.write(f"  a2 (Steepness 2)       = {popt_asc[4]:.4f} T^-1\n")
    f.write(f"  Hc2 (Coercive Field 2) = {popt_asc[5]:.4f} T\n")
    f.write(f"  Ms3 (Saturation Mag 3) = {popt_asc[6]:.4f} emu/g\n")
    f.write(f"  a3 (Steepness 3)       = {popt_asc[7]:.4f} T^-1\n")
    f.write(f"  Hc3 (Coercive Field 3) = {popt_asc[8]:.4f} T\n")
    f.write(f"  Ms4 (Saturation Mag 4) = {popt_asc[9]:.4f} emu/g\n")
    f.write(f"  a4 (Steepness 4)       = {popt_asc[10]:.4f} T^-1\n")
    f.write(f"  Hc4 (Coercive Field 4) = {popt_asc[11]:.4f} T\n")
    f.write(f"  Baseline               = {popt_asc[12]:.4f} emu/g\n")
    f.write("--------------------------------\n\n")
```

```
    f.write("Goodness-of-Fit Metrics (vs. Raw Data):\n")
    f.write("==========================================\n")
    f.write(f"  R-squared            = {r2_asc:.4f}\n")
    f.write(f"  RMSE                 = {rmse_asc:.4f} emu/g\n")
    f.write(f"  Chi-squared          = {chi2_asc:.4f}\n")
    f.write(f"  Reduced Chi-squared  = {reduced_chi2_asc:.4f}\n")
print(f"Ascending parameters exported to {output_params_asc_file}")

# Step 15: Plot the original hysteresis loop
plt.figure(figsize=(10, 6))
plt.plot(H_desc, M_g_desc, 'c.', label='Descending Data')
plt.plot(H_fit_desc, M_g_fit_desc, 'darkgreen', label='Descending Fit')
plt.plot(H_asc, M_g_asc, 'm.', label='Ascending Data')
plt.plot(H_fit_asc, M_g_fit_asc, 'purple', label='Ascending Fit')
plt.xlabel('Magnetic Field (T)')
plt.ylabel('Magnetic Moment per Gram (emu/g)')
plt.title('Hysteresis Loop with Scaled Arctangent Fits (X.X K)')
plt.legend()
plt.grid(True)
plt.show()

# Step 16: Plot the derivatives
plt.figure(figsize=(10, 6))
plt.plot(H_fit_desc, dM_dH_desc, 'darkgreen', label='Descending Fit Derivative')
plt.plot(H_fit_asc, dM_dH_asc, 'purple', label='Ascending Fit Derivative')
plt.xlabel('Magnetic Field (T)')
plt.ylabel('dM/dH (emu/g/T)')
plt.title('First Derivative of Hysteresis Loop Fits (X.X K)')
plt.legend()
plt.grid(True)
plt.show()
```

8 Python script used for deconvoluting the first derivative of the magnetic hysteresis loops using the Cauchy probability distribution function

```
import pandas as pd
import numpy as np
from scipy.optimize import curve_fit
import matplotlib.pyplot as plt
import os

# Step 1: Define the file path for the derivative data
input_file = "xxxx/HysFit_SArcTan_FitLinesDeriv.csv"
try:
    deriv_data = pd.read_csv(input_file)
except FileNotFoundError:
    print(f"Error: The file {input_file} was not found.")
    exit()

# Step 2: Extract descending and ascending branch derivatives
H_desc = deriv_data['Field_T'][~pd.isna(deriv_data['dM_dH_Descending_Fit'])].values
dM_dH_desc =
deriv_data['dM_dH_Descending_Fit'][~pd.isna(deriv_data['dM_dH_Descending_Fit'])].values
H_asc = deriv_data['Field_T'][~pd.isna(deriv_data['dM_dH_Ascending_Fit'])].values
dM_dH_asc =
deriv_data['dM_dH_Ascending_Fit'][~pd.isna(deriv_data['dM_dH_Ascending_Fit'])].values

# Step 3: Define the Cauchy PDF model with variable number of components
def cauchy_model(H, *params):
    """
    Sum of N Cauchy PDFs to fit dM/dH.
    Parameters: [P1, gamma1, HP1, P2, gamma2, HP2, ...] for N components.
    """
    n_components = len(params) // 3
    result = np.zeros_like(H, dtype=float)
    for i in range(n_components):
        P = params[3 * i]
        gamma = params[3 * i + 1]
        HP = params[3 * i + 2]
        result += P / (np.pi * gamma * (1 + ((H - HP) / gamma)**2))
    return result

# Step 4: Determine number of components based on observed peaks
from scipy.signal import find_peaks
peaks_desc, properties_desc = find_peaks(dM_dH_desc, height=0.03)
peaks_asc, properties_asc = find_peaks(dM_dH_asc, height=0.03)
n_components_desc = max(len(peaks_desc), 1)
n_components_asc = max(len(peaks_asc), 1)
```

```
if n_components_desc < 1:
    n_components_desc = 2
if n_components_asc < 1:
    n_components_asc = 2
print(f"Detected {n_components_desc} components for descending branch.")
print(f"Detected {n_components_asc} components for ascending branch.")

# Step 5: Set initial guesses and bounds for descending branch using peak positions
initial_guesses_desc = []
bounds_lower_desc = []
bounds_upper_desc = []
peak_heights_desc = properties_desc['peak_heights'] if len(peaks_desc) > 0 else
[max(dM_dH_desc)]
peak_positions_desc = H_desc[peaks_desc] if len(peaks_desc) > 0 else [0.0]
for i in range(n_components_desc):
    P_guess = peak_heights_desc[min(i, len(peak_heights_desc)-1)] * 3
    HP_guess = peak_positions_desc[min(i, len(peak_positions_desc)-1)]
    gamma_guess = 0.01 if abs(HP_guess) < 0.01 else 0.03
    initial_guesses_desc.extend([P_guess, gamma_guess, HP_guess])
    bounds_lower_desc.extend([0, 0.01, -7])
    bounds_upper_desc.extend([P_guess * 5, 1, 7])
p0_desc = np.array(initial_guesses_desc)
bounds_desc = (np.array(bounds_lower_desc), np.array(bounds_upper_desc))

# Step 6: Set initial guesses and bounds for ascending branch using peak positions
initial_guesses_asc = []
bounds_lower_asc = []
bounds_upper_asc = []
peak_heights_asc = properties_asc['peak_heights'] if len(peaks_asc) > 0 else [max(dM_dH_asc)]
peak_positions_asc = H_asc[peaks_asc] if len(peaks_asc) > 0 else [0.0]
for i in range(n_components_asc):
    P_guess = peak_heights_asc[min(i, len(peak_heights_asc)-1)] * 3
    HP_guess = peak_positions_asc[min(i, len(peak_positions_asc)-1)]
    gamma_guess = 0.1 if abs(HP_guess) < 0.01 else 0.03
    initial_guesses_asc.extend([P_guess, gamma_guess, HP_guess])
    bounds_lower_asc.extend([0, 0.01, -7])
    bounds_upper_asc.extend([P_guess * 5, 1, 7])
p0_asc = np.array(initial_guesses_asc)
bounds_asc = (np.array(bounds_lower_asc), np.array(bounds_upper_asc))

# Step 7: Fit the derivatives with the Cauchy model
try:
    popt_cauchy_desc, _ = curve_fit(
        cauchy_model, H_desc, dM_dH_desc, p0=p0_desc, bounds=bounds_desc, maxfev=60000
    )
    print("Cauchy fit parameters for descending branch:", popt_cauchy_desc)
except RuntimeError as e:
    print("Error fitting Cauchy model to descending branch:", e)

try:
    popt_cauchy_asc, _ = curve_fit(
```

```
        cauchy_model, H_asc, dM_dH_asc, p0=p0_asc, bounds=bounds_asc, maxfev=60000
    )
    print("Cauchy fit parameters for ascending branch:", popt_cauchy_asc)
except RuntimeError as e:
    print("Error fitting Cauchy model to ascending branch:", e)

# Step 8: Calculate individual Cauchy components and sort by HP with reversal for descending
def compute_components(H, params, is_descending=False):
    n_components = len(params) // 3
    components = []
    hp_indices = []
    for i in range(n_components):
        P = params[3 * i]
        gamma = params[3 * i + 1]
        HP = params[3 * i + 2]
        component = P / (np.pi * gamma * (1 + ((H - HP) / gamma)**2))
        components.append(component)
        hp_indices.append((i, HP))
    # Sort components by HP
    sorted_indices = [i[0] for i in sorted(hp_indices, key=lambda x: x[1])]
    if is_descending:
        sorted_indices = sorted_indices[::-1]  # Reverse order for descending branch
    sorted_components = [components[i] for i in sorted_indices]
    return sorted_components, sorted_indices

# Descending branch (reverse order)
components_desc, sorted_indices_desc = compute_components(H_desc, popt_cauchy_desc,
is_descending=True)
total_P_desc = sum(popt_cauchy_desc[::3])
percent_contrib_desc = [(popt_cauchy_desc[3 * sorted_indices_desc[i]] / total_P_desc * 100) for
i in range(n_components_desc)]
Ms_desc = [(popt_cauchy_desc[3 * sorted_indices_desc[i]] * popt_cauchy_desc[3 *
sorted_indices_desc[i] + 1]) / 2 for i in range(n_components_desc)]

# Ascending branch (normal order)
components_asc, sorted_indices_asc = compute_components(H_asc, popt_cauchy_asc,
is_descending=False)
total_P_asc = sum(popt_cauchy_asc[::3])
percent_contrib_asc = [(popt_cauchy_asc[3 * sorted_indices_asc[i]] / total_P_asc * 100) for i in
range(n_components_asc)]
Ms_asc = [(popt_cauchy_asc[3 * sorted_indices_asc[i]] * popt_cauchy_asc[3 *
sorted_indices_asc[i] + 1]) / 2 for i in range(n_components_asc)]

# Step 9: Compute goodness-of-fit metrics
def goodness_of_fit(y_true, y_pred, n_params):
    mask = np.isfinite(y_true) & np.isfinite(y_pred)
    y_true = y_true[mask]
    y_pred = y_pred[mask]

    ss_tot = np.sum((y_true - np.mean(y_true))**2)
    ss_res = np.sum((y_true - y_pred)**2)
```

```
    r_squared = 1 - ss_res / ss_tot if ss_tot != 0 else np.nan

    rmse = np.sqrt(np.mean((y_true - y_pred)**2))

    chi_squared = np.sum((y_true - y_pred)**2)
    dof = len(y_true) - n_params
    reduced_chi_squared = chi_squared / dof if dof > 0 else np.nan

    return r_squared, rmse, chi_squared, reduced_chi_squared

# Compute metrics for descending branch
cauchy_fit_desc = cauchy_model(H_desc, *popt_cauchy_desc)
r2_desc, rmse_desc, chi2_desc, reduced_chi2_desc = goodness_of_fit(dM_dH_desc,
cauchy_fit_desc, len(popt_cauchy_desc))

# Compute metrics for ascending branch
cauchy_fit_asc = cauchy_model(H_asc, *popt_cauchy_asc)
r2_asc, rmse_asc, chi2_asc, reduced_chi2_asc = goodness_of_fit(dM_dH_asc, cauchy_fit_asc,
len(popt_cauchy_asc))

# Step 10: Export fits and parameters
output_dir = os.path.dirname(input_file)

# Export descending fit to CSV
output_fit_desc_file = os.path.join(output_dir,
"HysFit_SArcTan_CauchyFit_Descending_X.XK.csv")
fit_desc_data = {
    'Field_T': H_desc,
    'dM_dH_Descending_Fit': dM_dH_desc,
    'Cauchy_Fit': cauchy_model(H_desc, *popt_cauchy_desc)
}
# Add individual components to the dictionary
for i, comp in enumerate(components_desc):
    fit_desc_data[f'Descending_P_{i+1}'] = comp
fit_desc_df = pd.DataFrame(fit_desc_data)
fit_desc_df.to_csv(output_fit_desc_file, index=False)
print(f"Descending fit exported to {output_fit_desc_file}")

# Export ascending fit to CSV
output_fit_asc_file = os.path.join(output_dir,
"HysFit_SArcTan_CauchyFit_Ascending_X.XK.csv")
fit_asc_data = {
    'Field_T': H_asc,
    'dM_dH_Ascending_Fit': dM_dH_asc,
    'Cauchy_Fit': cauchy_model(H_asc, *popt_cauchy_asc)
}
fit_asc_df = pd.DataFrame(fit_asc_data)
fit_asc_df.to_csv(output_fit_asc_file, index=False)
print(f"Ascending fit exported to {output_fit_asc_file}")

# Export total Cauchy fit for descending branch
```

```
output_total_desc_file = os.path.join(output_dir,
"HysFit_SArcTan_TotalCauchyFit_Descending_X.XK.csv")
total_desc_data = {
    'Field_T': H_desc,
    'Total_Cauchy_Fit': cauchy_model(H_desc, *popt_cauchy_desc)
}
# Add individual components to the dictionary
for i, comp in enumerate(components_asc):
    fit_asc_data[f'Ascending_P_{i+1}'] = comp
total_desc_df = pd.DataFrame(total_desc_data)
total_desc_df.to_csv(output_total_desc_file, index=False)
print(f"Total Cauchy fit for descending branch exported to {output_total_desc_file}")

# Export total Cauchy fit for ascending branch
output_total_asc_file = os.path.join(output_dir,
"HysFit_SArcTan_TotalCauchyFit_Ascending_X.XK.csv")
total_asc_data = {
    'Field_T': H_asc,
    'Total_Cauchy_Fit': cauchy_model(H_asc, *popt_cauchy_asc)
}
total_asc_df = pd.DataFrame(total_asc_data)
total_asc_df.to_csv(output_total_asc_file, index=False)
print(f"Total Cauchy fit for ascending branch exported to {output_total_asc_file}")

# Export descending parameters to text file with percent contribution, Ms, and goodness-of-fit
output_params_desc_file = os.path.join(output_dir,
"HysFit_SArcTan_CauchyFit_Parameters_Descending_X.XK.txt")
with open(output_params_desc_file, 'w') as f:
    f.write("Cauchy Fit Parameters for Descending Branch (X.X K):\n")
    f.write("=============================================\n\n")
    for i in range(n_components_desc):
        idx = sorted_indices_desc[i]
        P = popt_cauchy_desc[3 * idx]
        gamma = popt_cauchy_desc[3 * idx + 1]
        HP = popt_cauchy_desc[3 * idx + 2]
        perc = percent_contrib_desc[i]
        Ms = Ms_desc[i]
        f.write(f"Component P_{i+1}:\n")
        f.write(f"  Amplitude (P)        = {P:.4f} emu/g/T\n")
        f.write(f"  Half-Width (gamma)    = {gamma:.4f} T\n")
        f.write(f"  Peak Field (HP)       = {HP:.4f} T\n")
        f.write(f"  Percent Contribution  = {perc:.2f}%\n")
        f.write(f"  Saturation Mag (Ms)   = {Ms:.4f} emu/g\n")
        f.write("-------------------------------\n\n")
    f.write("Goodness-of-Fit Metrics (vs. Derivative Data):\n")
    f.write("=============================================\n")
    f.write(f"  R-squared           = {r2_desc:.4f}\n")
    f.write(f"  RMSE                = {rmse_desc:.4f} emu/g/T\n")
    f.write(f"  Chi-squared         = {chi2_desc:.4f}\n")
    f.write(f"  Reduced Chi-squared  = {reduced_chi2_desc:.4f}\n")
print(f"Descending parameters exported to {output_params_desc_file}")
```

```
# Export ascending parameters to text file with percent contribution, Ms, and goodness-of-fit
output_params_asc_file = os.path.join(output_dir,
"HysFit_SArcTan_CauchyFit_Parameters_Ascending_X.XK.txt")
with open(output_params_asc_file, 'w') as f:
    f.write("Cauchy Fit Parameters for Ascending Branch (X.X K):\n")
    f.write("===============================================\n\n")
    for i in range(n_components_asc):
        idx = sorted_indices_asc[i]
        P = popt_cauchy_asc[3 * idx]
        gamma = popt_cauchy_asc[3 * idx + 1]
        HP = popt_cauchy_asc[3 * idx + 2]
        perc = percent_contrib_asc[i]
        Ms = Ms_asc[i]
        f.write(f"Component P_{i+1}:\n")
        f.write(f"  Amplitude (P)        = {P:.4f} emu/g/T\n")
        f.write(f"  Half-Width (gamma)    = {gamma:.4f} T\n")
        f.write(f"  Peak Field (HP)       = {HP:.4f} T\n")
        f.write(f"  Percent Contribution  = {perc:.2f}%\n")
        f.write(f"  Saturation Mag (Ms)   = {Ms:.4f} emu/g\n")
        f.write("------------------------------\n\n")
    f.write("Goodness-of-Fit Metrics (vs. Derivative Data):\n")
    f.write("===============================================\n")
    f.write(f"  R-squared           = {r2_asc:.4f}\n")
    f.write(f"  RMSE                = {rmse_asc:.4f} emu/g/T\n")
    f.write(f"  Chi-squared         = {chi2_asc:.4f}\n")
    f.write(f"  Reduced Chi-squared  = {reduced_chi2_asc:.4f}\n")
print(f"Ascending parameters exported to {output_params_asc_file}")

# Step 11: Plot descending branch with total fit
plt.figure(figsize=(10, 6))
plt.plot(H_desc, dM_dH_desc, 'darkgreen', label='Descending Fit Derivative')
plt.plot(H_desc, cauchy_model(H_desc, *popt_cauchy_desc), 'k-', label='Total Cauchy Fit',
alpha=0.7)
for i, comp in enumerate(components_desc):
    plt.plot(H_desc, comp, ['g--', 'y--', 'b--'][i % 3], label=f'Descending P_{i+1}')
plt.xlabel('Magnetic Field (T)')
plt.ylabel('dM/dH (emu/g/T)')
plt.title('Descending Branch Derivative with Cauchy Components (X.X K)')
plt.legend()
plt.grid(True)
plt.show()

# Step 12: Plot ascending branch with total fit
plt.figure(figsize=(10, 6))
plt.plot(H_asc, dM_dH_asc, 'purple', label='Ascending Fit Derivative')
plt.plot(H_asc, cauchy_model(H_asc, *popt_cauchy_asc), 'k-', label='Total Cauchy Fit',
alpha=0.7)
for i, comp in enumerate(components_asc):
    plt.plot(H_asc, comp, ['g-.', 'y-.', 'b-.'][i % 3], label=f'Ascending P_{i+1}')
plt.xlabel('Magnetic Field (T)')
```

```
plt.ylabel('dM/dH (emu/g/T)')
plt.title('Ascending Branch Derivative with Cauchy Components (X.X K)')
plt.legend()
plt.grid(True)
plt.show()

# Step 13: Plot percent contributions as bar charts
fig, (ax1, ax2) = plt.subplots(1, 2, figsize=(12, 6))
n_comp_desc = len(percent_contrib_desc)
ax1.bar(range(n_comp_desc), percent_contrib_desc, color=['green', 'yellow',
'blue'][:n_comp_desc])
ax1.set_xticks(range(n_comp_desc))
ax1.set_xticklabels([f'P_{i+1}' for i in range(n_comp_desc)])
ax1.set_title('Descending Branch Percent Contributions (X.X K)')
ax1.set_ylabel('Percent Contribution (%)')
n_comp_asc = len(percent_contrib_asc)
ax2.bar(range(n_comp_asc), percent_contrib_asc, color=['green', 'yellow', 'blue'][:n_comp_asc])
ax2.set_xticks(range(n_comp_asc))
ax2.set_xticklabels([f'P_{i+1}' for i in range(n_comp_asc)])
ax2.set_title('Ascending Branch Percent Contributions (X.X K)')
ax2.set_ylabel('Percent Contribution (%)')
plt.tight_layout()
plt.show()
```
